# Supplementary figures and images for: CRISPR Screen Reveals that EHEC’s T3SS and Shiga Toxin Rely on Shared Host Factors for Infection
Source: mBio. 2018 Jun 19;9(3):e01003-18. doi: 10.1128/mBio.01003-18 (PMC6016243; doi:10.1128/mBio.01003-18)

Fig. S1

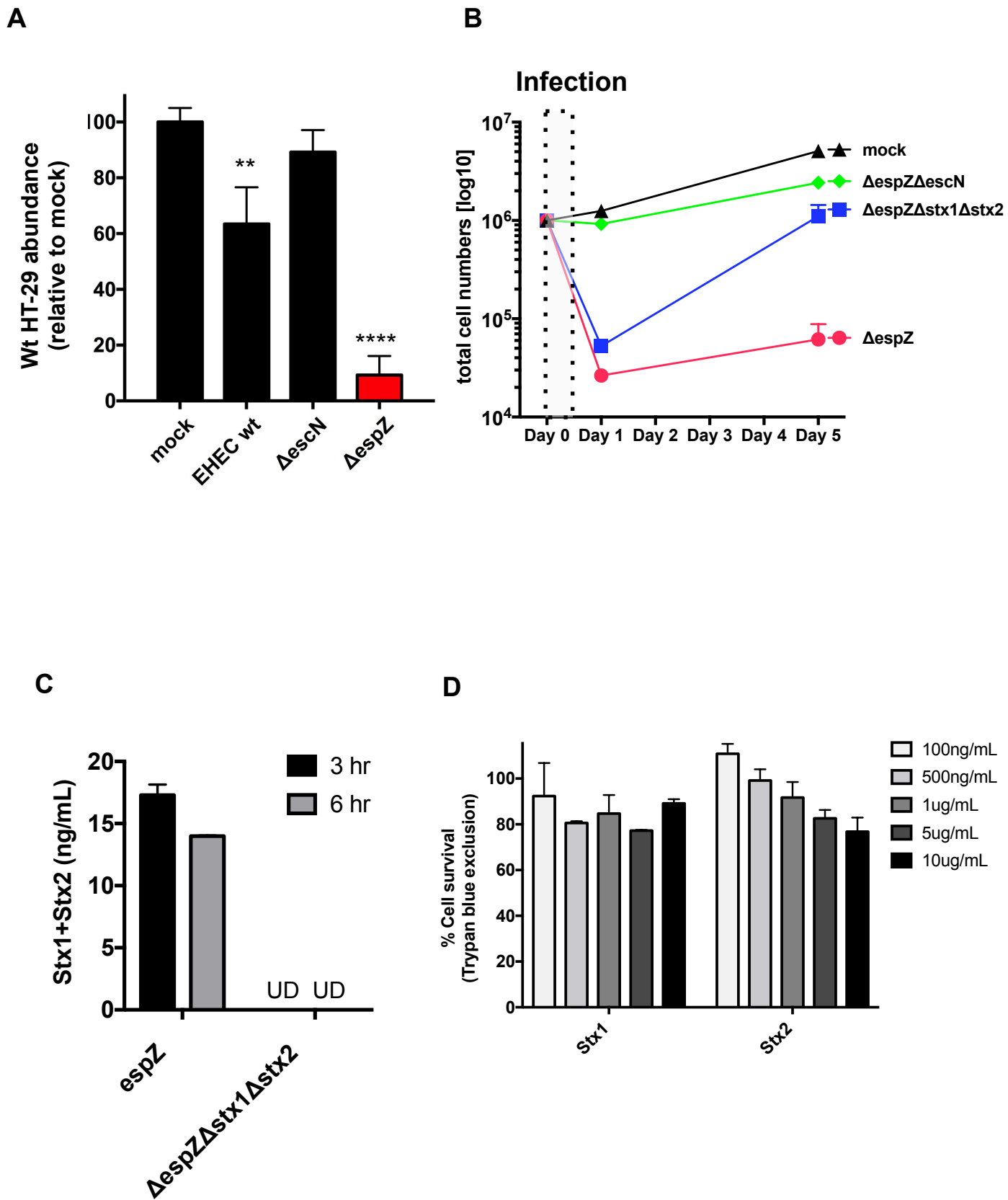

Supplement: FIG S1 [file mbo003183919sf1.pdf]

**Fig. S2**

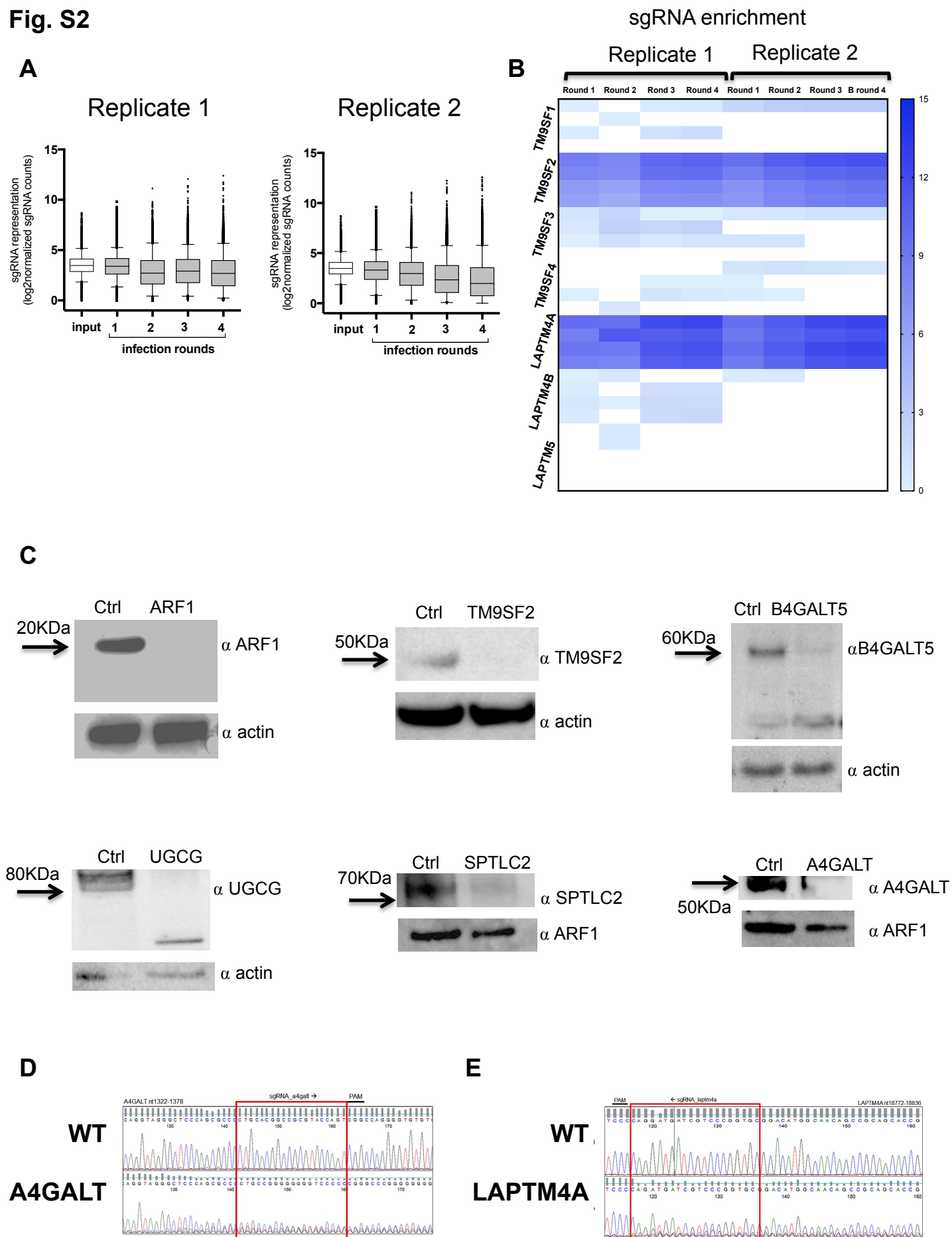

Supplement: FIG S2 [file mbo003183919sf2.pdf]

Fig. S3

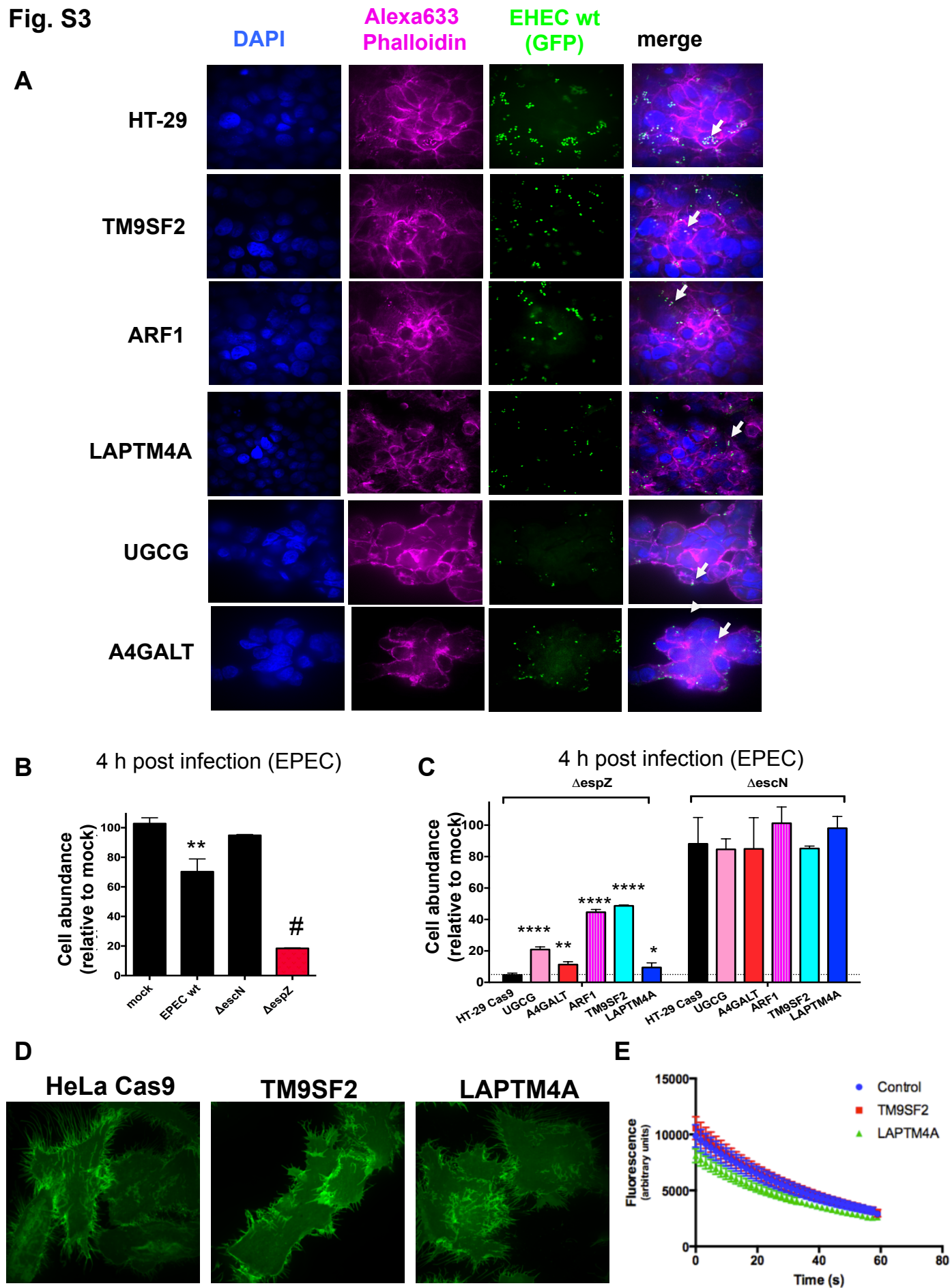

Supplement: FIG S3 [file mbo003183919sf3.pdf]

**Fig. S4**

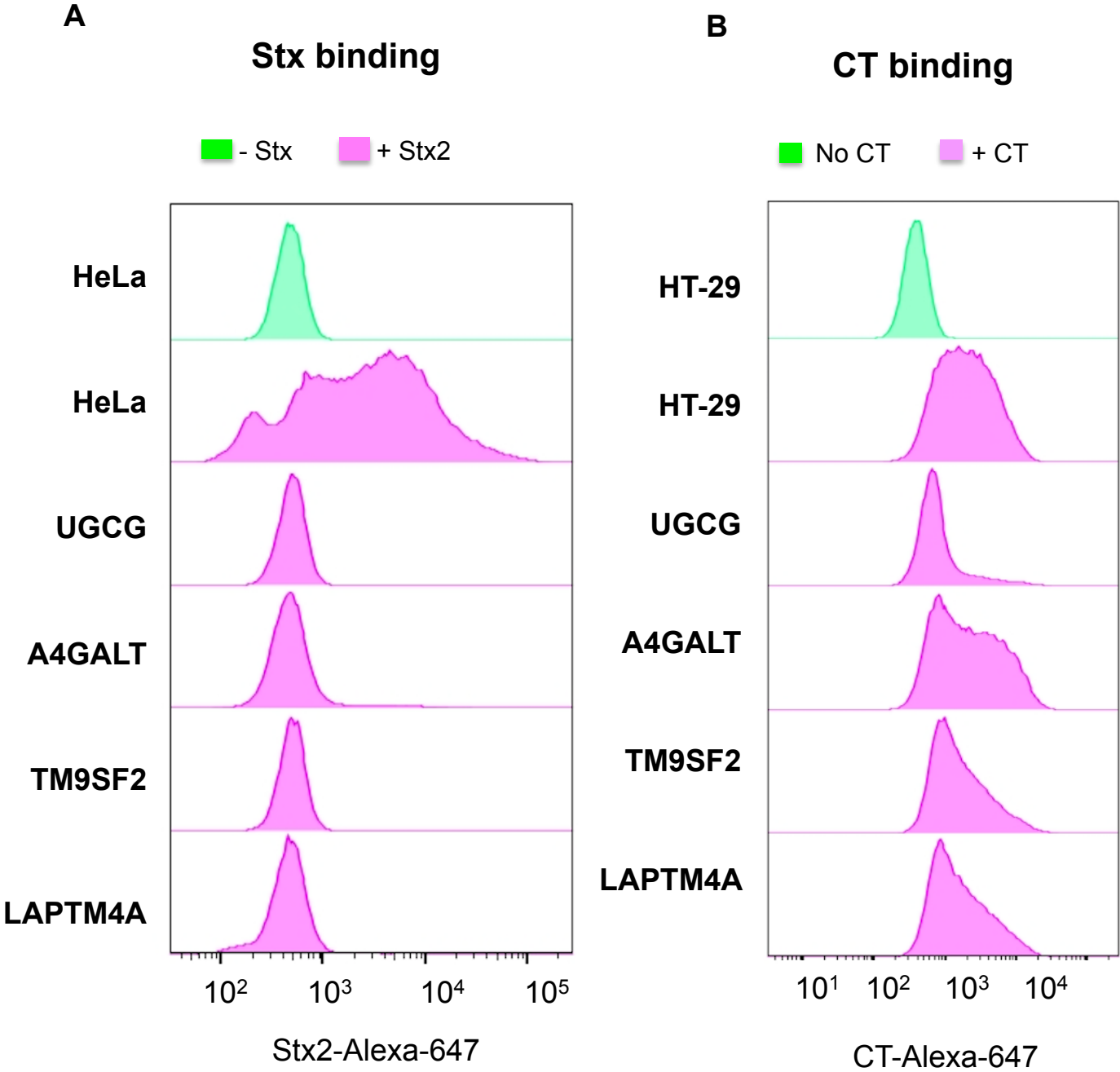

Supplement: FIG S4 [file mbo003183919sf4.pdf]

**Fig. S5**

**A**

Cis-medial  
Golgi  
(GM130)

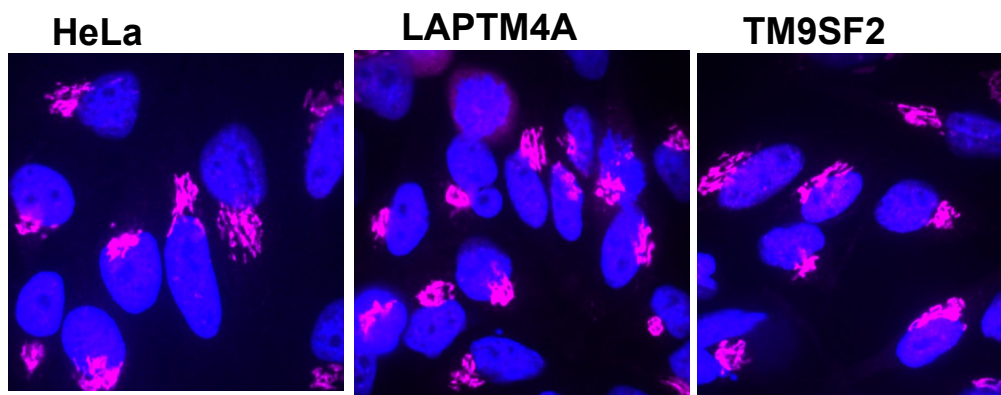

**B**

Trans-Golgi  
network

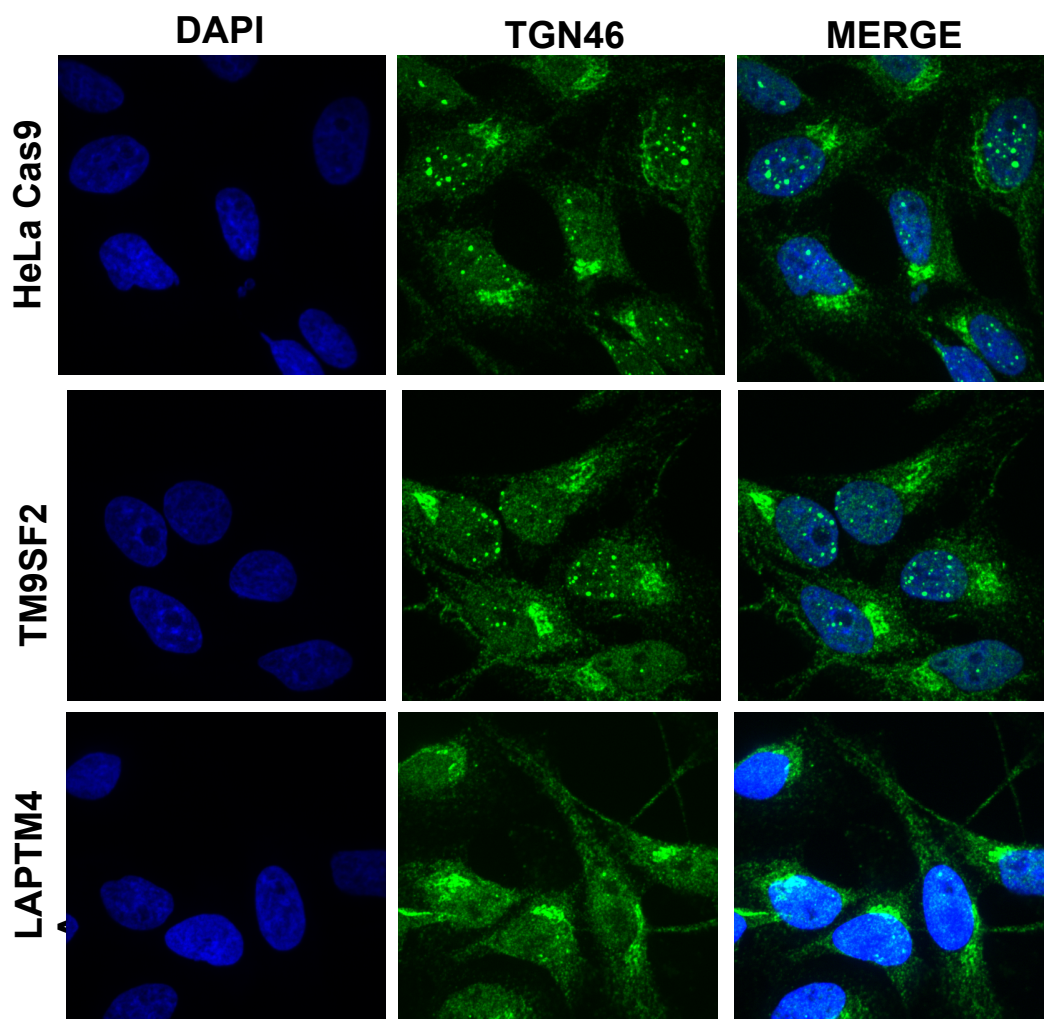

**C**

TGN  
measure-  
ments

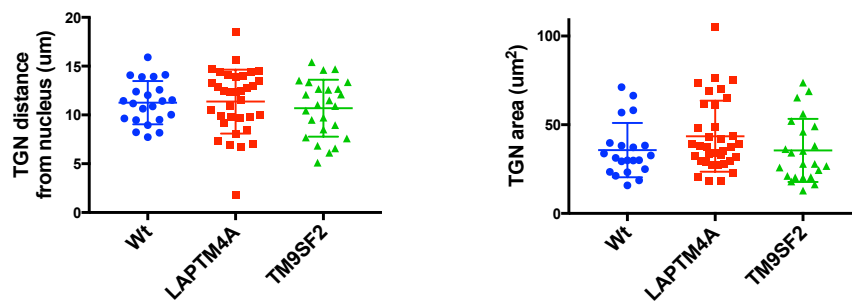

Supplement: FIG S5 [file mbo003183919sf5.pdf]
