# Supplementary material for: CRISPR Screen Reveals that EHEC’s T3SS and Shiga Toxin Rely on Shared Host Factors for Infection
Source: mBio. 2018 Jun 19;9(3):e01003-18. doi: 10.1128/mBio.01003-18 (PMC6016243; doi:10.1128/mBio.01003-18)
Supplement: TABLE S1 [file mbo003183919st1.pdf]

Supplementary Table S1. Primers used to construct EHEC EDL933 mutants.

**Generation of espZ by allelic exchange**	
**EspZ-Afor;**	**AGTACGCGTCACTAGTGGGGCCCTTCTAGAAAAACATCGATATTGGTTAACAAAC**
**EspZ-Arev;**	**GGCATATTTCATCGCTAATGCACCGCCTCTAGACCAGAAGGACTTAAATTTGCTGCTTCC**
**EspZ-Bfor;**	**GGAAGCAGCAAATTTAAGTCCTTCTGGTCTAGAGGCGGTGCATTAGCGATGAAATATGCC**
**EspZ-Brev;**	**TAACAATTTGTGGAATTCCCGGGAGAGCTCTTTTCATTTTTATTGCATCGAAAC**
**Generation of stx1, stx2 and escN mutants by Lambda-red recombination**	
**Stx2AB-F**	**ATGAAGTGTATATTATTTAAATGGGTACTGTGCCTGGTGTAGGCTGGAGCTGCTTCG**
**Stx2AB-Re**	**TAAGGAGGATATTCATATGTTGCTGAAGTGCAGTTTAATAATGACTGAGGCATAA**
**Stx1AB-F**	**TCGTATGGTGCTCAAGGAGTATTGTGTAATATGAAAATAATTATTTTTAGAGTGCTGTGTAGGCTGGAGCTGCTTCG**
**Stx1AB-Re**	**GCCTGCTATTTTCACTGAGCTATTCTAAGTCAACGAAAAATAACTTCGCTGAATCCATATGAATATCCTCCTTA**
**EscN-F**	**ATGATTTCAGAGCATGATTCTGTATTGGAAAAATACGTGTAGGCTGGAGCTGCTTCG**
**EscN-Rev**	**GGCAACACTTTGAATAGGCTTTCAATCGTTTTTTCCATATGAATATCCTCCTTA**
