# Supplementary material for: CRISPR Screen Reveals that EHEC’s T3SS and Shiga Toxin Rely on Shared Host Factors for Infection
Source: mBio. 2018 Jun 19;9(3):e01003-18. doi: 10.1128/mBio.01003-18 (PMC6016243; doi:10.1128/mBio.01003-18)
Supplement: TABLE S2 [file mbo003183919st2.pdf]

Table S2. STARS analysis of Library B round 4

p-value=0 means <1.03950212016e-06, FDR=0 means <0.000156816319841

Gene Symb	Number of p	Ranks of perturbations	Perturbations	Most enrich	STARS Sco	Average Sc	p-value	FDR	q-value
A4GALT	4	2;3;4;5	AGTGAGATGCCCAGGTGCCG;ACTGC	4	16.6971	10.223235	0	0	0
LAPTM4A	4	1;6;7;8	TTACCATGTACCAGGTCCCC;GCACC	4	15.88062	9.7615876	0	0	0
UGCG	4	11;14;20;25	CCTTACGTAGCAGACAGACA;TCTTCT	4	13.90122	8.4805378	0	0	0
TM9SF2	4	10;12;21;37	TGGATAATATGCCTGTAACG;AAACAA	4	13.220173	8.338197	0	0	0
DUSP6	4	39;94;111;151	CATCGAGTCGGCCATCAACG;GTGCG	4	10.777072	6.5908506	0	0	0
SPTLC2	4	42;181;315;325	GCTCCAGGCACACTACAGAT;ACAACT	4	9.4454466	5.7684458	0	0	0
B4GALT5	4	22;30;49;396	GAGAGAAAAGAAGAAGAGCG;TCACA	4	9.1021993	6.7482906	0	0	0
SPTSSA	4	43;54;106;2970	GTTCTACTACCAGTACCTGC;CTACCA	3	7.9423743	5.4217437	1.04E-06	0.0001372	0.0001372
TFAP4	4	114;177;421;1334	AGATCGCCAACAGCAACGAG;GCTTC	4	6.9923567	4.957591	5.20E-06	0.0006098	0.0006098
KLF5	4	307;1123;2207;2209	GTACCAGCTACTGAATACAC;GTCTTG	4	6.1161972	3.696411	3.95E-05	0.0041713	0.0041713
KDSR	4	1130;1283;1732;2378	TTAATGAGCATCAATTACCT;TCACCAC	4	5.9881327	3.5763149	5.30E-05	0.0050894	0.0050894
TMEM165	4	178;432;611;2780	ATAGACCCTGGGGATGACTG;TGCCA	4	5.7168009	4.2765509	9.15E-05	0.0080499	0.0080499
NEK9	4	639;1032;1215;3260	TTCCAGCTCAATCAGCAGCG;GATAAC	4	5.4401097	3.6602808	0.0001767	0.0143547	0.0135646
ADNP	4	364;365;730;30179	GTAAATAGCGATTACAGTAG;GGATGC	3	5.4319722	3.6654765	0.0001798	0.0135646	0.0135646
MLLT3	4	1185;1401;1884;3595	GGAATAAGTCATAATCAAAG;GTCGTG	4	5.2701845	3.3459818	0.0002723	0.0191734	0.0191734
HFE	4	532;767;897;52394	CGGTCTGCAAAAGCATCAGG;ACTAG	3	5.1645452	3.3089488	0.000341	0.0225031	0.0225031
TADA1	4	85;340;3484;4200	GCAAAACCTGGAAAACCCAA;GGCCA	4	4.9999829	3.666191	0.0005239	0.032544	0.0310409
ZNF217	4	236;740;1029;5187	AAGAACTGCTGGTTCACAGA;GCGCA	3	4.986454	3.6901725	0.0005291	0.0310409	0.0310409
SPPL3	4	416;614;1071;14945	CAGGCTCTGTTCCTTCCAAT;TTAAATA	3	4.9345794	3.3310402	0.0006102	0.0339136	0.0339136
ARF1	4	780;993;1257;5734	GCTGATGTTCTTGTACTCCA;CCCGCA	3	4.7270407	3.3912562	0.0009792	0.0517023	0.0517023
RALGAPB	4	2048;2718;4034;5120	CTATGGACTGACCCTTCCAT;GTCTTC	4	4.6559002	2.7521504	0.0011549	0.0580743	0.0580743
PARD6B	4	410;2114;3146;5448	TTATAGGTAGTAAGTCTCCA;TCTTCAC	4	4.5480317	3.0249406	0.0014584	0.0700042	0.0700042
USP9X	4	2761;2972;3715;5983	ACAAGTGCTATATCTAACAG;TTTCCC	4	4.385304	2.6629919	0.0021227	0.0974579	0.0974579
HSD17B4	4	550;3467;5952;6070	GATTGACGCTCCCACTCCAA;TGACA	4	4.3602253	2.6400371	0.0022599	0.0994346	0.0994346
C20orf24	4	779;1885;5666;6369	CCGTCTGGAGTAAGGTGCTG;ACTCC	4	4.2766951	2.725508	0.0027453	0.1159625	0.1159625
ST20-MTHF	4	447;1009;1891;33008	CAGGAGACTCACCTGAACCT;CACCT	3	4.198738	2.9332691	0.0032765	0.1330767	0.1306687
KDM6A	4	1730;1915;4870;6693	TGAGGAAAAGAAAATGGCGG;TCAGC	4	4.1904968	2.6662761	0.003341	0.1306687	0.1306687
TADA2B	4	898;2699;5739;6843	GGGCGGCTGGACCAGTCGCG;TCAG	4	4.1519939	2.5998638	0.0036351	0.1370967	0.1369055
SPTLC1	4	149;262;4049;15288	GCTAACTATGGCGACCGCCA;AAAGA	2	4.1347885	3.1517425	0.0037931	0.1381227	0.1369055
GCC1	4	1309;3155;6554;6969	GGTGCTGTCGGTATCCCACG;CCTGG	4	4.1202982	2.4783634	0.0039439	0.1388243	0.1369055
SAMD15	4	110;270;66697;68546	GGAAGAGGACTTCAAAGAGG;TACCA	2	4.108757	3.1702348	0.0040499	0.1379579	0.1369055
SNRPD2	4	52;273;62835;63263	GGAGCTGCAGAAGCGAGAGG;GAGG	2	4.0991942	3.3276417	0.0041487	0.1369055	0.1369055
CCM2L	4	1838;6218;6688;7236	GATGCTGTCCTGCTCCGCCG;GCAGC	4	4.0549858	2.2833394	0.004606	0.1473931	0.1473931
PSMA6	4	774;1601;2268;62298	GCATACTTACAACAACCAAG;GTATGG	3	3.9641384	2.6461776	0.0055135	0.1712434	0.1712434
FAM184A	4	223;329;16685;56929	TTGAGGAGCTAAGACTTGAA;GGAGG	2	3.9377816	2.9322789	0.0057412	0.1732193	0.1732193
AK5	4	271;397;23289;24931	CTGAAGCGTGCAGAACAGCA;ACTCA	2	3.7753871	2.8091694	0.0075114	0.2203356	0.2203356
SPAG11A	5	282;318;20615;33042;647	AGGAGAAGGCTGGTGACGGA;CCCG	2	3.747195	2.7388962	0.0078971	0.2253874	0.2253874
RNF165	4	231;426;4546;45025	ATAGCTCTCCCGTACGGCGG;GCAGA	2	3.714488	2.8993856	0.0083992	0.2334087	0.2334087
MAP2K4	4	1475;2180;2799;59726	GCATGCTGCTGACGGCCGGG;GACC	3	3.693255	2.3795974	0.0087318	0.2364308	0.2364308
SCUBE1	4	1586;2332;2885;27139	GCTGCAGTGTGAATCCAACG;TGACC	3	3.6543461	2.3378759	0.0094096	0.2484127	0.2484127
LSM8	8	140;216;20518;34958;462	GGATTCGGACGGAGAGCGCG;GTTG	2	3.6379715	2.7338512	0.0097193	0.2503324	0.2503324
ADCY1	4	113;1366;2960;7752	CCGCCGCCGCCTCCGCCGCG;TTCT	3	3.6213623	2.8516374	0.0100187	0.2518993	0.2518993
EZH2	4	1567;3038;3108;15566	TTATGATGGGAAAGTACACG;TTATCA	3	3.5586914	2.233889	0.0113628	0.2790492	0.2790492
POLR3C	4	242;536;30978;59575	CCCAGGGTCTGAATTCTCAG;TTGGT	2	3.5162652	2.7039317	0.0123784	0.2970814	0.293358
TAF5L	4	213;1358;3227;8043	TGTCCTGCTACCTCAAACGC;AAACAC	3	3.5104602	2.7251548	0.0125011	0.293358	0.293358
SLC39A10	4	219;2363;3272;13278	TCTGATGTTAAACTACCGAA;CCATTAC	3	3.4926907	2.5588647	0.0129844	0.2980771	0.2980771
HES1	4	1647;2446;3332;24136	CTTCAGCGAGTGCATGAACG;TGTCG	3	3.4693807	2.2577578	0.0136227	0.3060754	0.3060754
CERS2	4	304;2542;3531;53380	TTCTTCATGTCATAGAACCA;GCTGCC	3	3.3950154	2.458902	0.0158223	0.3480898	0.3427781
TNFAIP8L2	4	327;3307;3539;21888	GCGTGTACTCCTTGGACACA;GATCA	3	3.3921157	2.3744257	0.0159054	0.3427781	0.3427781
C2orf27A	4	277;635;5902;22852	TTGAGCCAGTGCCAGCACCA;CAGCC	2	3.3702078	2.648061	0.0166352	0.3513344	0.3456939
GNAT3	4	2317;3344;3607;49765	TTATGCAATGGCAAATACCC;ACTGGA	3	3.3677342	2.0915487	0.0166954	0.3456939	0.3456939
ZHX3	4	2862;3567;3636;18474	GGACCATTCCTTCATCAATG;AGATCT	3	3.3574783	2.043047	0.0170769	0.3467933	0.3467933
DUSP15	4	1527;2286;3664;26989	CGTATGTGATGACTGTGACG;CGCGC	3	3.3476547	2.2463673	0.0174366	0.3474162	0.3471216
C1QBP	4	392;659;10068;59856	GCGTGCGCGCAGGTTCCGAG;CCAC	2	3.3382659	2.5115106	0.0177505	0.3471216	0.3471216
FAM65C	4	1195;3553;3729;34528	CCAAGATGTACGGCACGCTG;GCAGG	3	3.3251416	2.1498192	0.0182162	0.3497517	0.3497517
CDK5RAP1	4	656;2831;3801;27210	GCGGACCAGTAACCTCCAAG;GCCTA	3	3.300666	2.2881548	0.0191902	0.3618733	0.3576467
TSPAN33	4	361;696;15187;36572	GACGAAGCCCAGGATCCCAG;AGAGC	2	3.2912525	2.5056216	0.0195832	0.3628042	0.3576467
ZNF410	4	302;1890;3833;22821	TGAGTTAGAATCCAAACCAG;GCACAC	3	3.2899392	2.5080248	0.0196435	0.3576467	0.3576467
ORAOV1	4	1083;3357;3889;71197	CCTCTCATCCGCCATCACGA;AGTAGT	3	3.2713853	2.1611181	0.0204013	0.3651481	0.3651481
SLTM	4	230;757;24473;37293	AACTATGTCTTCAAGCACAG;CCAGAC	2	3.2189956	2.5662356	0.022841	0.4020012	0.3982363
CNBD2	4	450;2950;4063;18919	GAAGCCTTCCTGGAGAACAG;GATATT	3	3.2154273	2.3016573	0.0230042	0.3982363	0.3982363
2-Sep	4	36;781;28887;31754	TGATTTGGTCTCCAAAGCCT;GGAGAA	2	3.1921673	2.953839	0.0242069	0.4122979	0.4122979
C17orf50	4	80;794;46545;48798	GGACAGCGCGACGGAGGGCG;ACGA	2	3.1779812	2.7737363	0.0250364	0.4196579	0.416068
ZNF629	4	1216;3673;4194;7624	GCACTTGTAGGGCTTCTCGC;AGAGA	3	3.1748888	2.0882088	0.0252162	0.416068	0.416068
C2orf27B	5	277;635;22852;42336;487	TTGAGCCAGTGCCAGCACCA;CAGCC	2	3.1520672	2.4451587	0.0263711	0.4284294	0.4252792
LBP	4	2676;3389;4359;29863	CACGCTGCCTGACTTCACCG;ATAGC	3	3.1256312	1.9884665	0.0278233	0.445173	0.4252792
MYOZ2	4	623;846;8427;10524	TTATCCCATCTCAGTAACCG;TCTTAA	2	3.1234929	2.3055517	0.0279855	0.4410845	0.4252792
ANKRD28	4	1093;1832;4390;67567	CTACACATGACTGCTCTCCA;TTAATG	3	3.1165897	2.2774738	0.0284158	0.4412811	0.4252792
SYF2	4	300;854;19974;34514	GATGGGAGAGGAAAAAGAAG;GGTGC	2	3.115412	2.4573599	0.0284834	0.4359198	0.4252792
USP22	4	143;855;5600;13732	CCCAGGTGCGAGCAGCCCGG;GATG	2	3.1144073	2.6799043	0.0285447	0.4306176	0.4252792
DNMT3B	4	2271;3846;4401;44524	GGTCCTCCAATGAGTCTCCA;GGGGA	3	3.1133971	1.9709458	0.0285936	0.4252792	0.4252792
LCN10	4	2866;3496;4488;60621	GCGTGCTCCTCGCCTTCAGA;GGTGC	3	3.0884306	1.9587186	0.0302225	0.4432631	0.4394466

FOXS1	4	2851;3055;4498;8499	TGGCCATCCAGAGCTCACCG;GTAGC	3	3.0855927	1.9956938	0.0303784	0.4394466	0.4394466
NUSAP1	4	390;1353;4518;71387	TCATGATTCTGGAATTCAGT;GGGTCT	3	3.0799361	2.4961667	0.0308056	0.4396049	0.4396049
PODN	4	314;4243;4597;18069	TGAGACAAGGACTCACCAGA;GCTCG	3	3.0578405	2.2004599	0.0323441	0.455405	0.4422316
GFOD1	4	1936;2155;4612;10740	ATCCCGCTGCTGAAAGACGA;GCTGA	3	3.0536891	2.1330206	0.0326528	0.4537026	0.4422316
ATXN7L3	4	523;4016;4618;19975	GCAGCCGAATCGCCAACCGC;CATCG	3	3.0520324	2.1408767	0.032762	0.4493073	0.4422316
LY6G5B	4	601;927;33700;65844	CTGAAATGCATCCTACAGAA;CTACAG	2	3.0450275	2.2739323	0.0332464	0.4501051	0.4422316
CPSF6	4	1116;4443;4657;17991	GCATTTCCACAAGGTGGTAG;GGACC	3	3.0413172	2.0035703	0.0334834	0.4475756	0.4422316
BCAS4	4	1001;3881;4687;12628	GCCGGAGCCCATGCGCAGCG;TGATA	3	3.0331372	2.0527565	0.0340634	0.4496375	0.4422316
XKR7	4	1999;2677;4699;22244	ACGACTACTCGGAGCCCGCA;AGTAA	3	3.0298802	2.0601152	0.0343316	0.4475828	0.4422316
POLR2C	4	670;3308;4700;57065	GCACTGACCGGAATGACCCG;AGTGA	3	3.0296091	2.1516723	0.03434	0.4422316	0.4422316
OCSTAMP	4	598;4038;4810;16836	ATTGCTACCTGACAGACCTG;GGTATT	3	3.0001506	2.1031308	0.0365915	0.4655499	0.4601644
SUN5	4	50;977;5860;61349	CCCACAATCCCAGACCCCGG;GCTGC	2	2.9999869	2.7741809	0.036604	0.4601644	0.4601644
DEFB116	4	992;4264;4853;25725	AATCCATGTGAGCTTTACCA;TTGAGC	3	2.9888223	2.0135607	0.0375302	0.4662574	0.4662574
PRR5L	4	4012;4444;4900;37659	GGGAAGGTCGCTGAGCACGG;TCAC	3	2.9765573	1.8141312	0.0385271	0.4730765	0.4675265
ST20	4	447;1009;30208;36618	CAGGAGACTCACCTGAACCT;CACCT	2	2.9723708	2.3005347	0.038893	0.4720802	0.4675265
ITGA6	4	125;1010;11230;14328	AGAGAGCCAACAGAACGGGA;AATCC	2	2.9715222	2.5739898	0.0389605	0.4675265	0.4675265
NR2E1	4	3316;3482;4994;57349	TGCGCCGGCCTTCTTCACCG;GCACG	3	2.9523853	1.8960919	0.0406144	0.4818966	0.4811891
FSTL5	4	28;1038;37786;51534	TCTCCGTCAGATCCACACAC;GCAGT	2	2.9481004	2.886308	0.0410104	0.4811891	0.4811891
RREB1	4	206;1825;5041;27023	GCACAAGCAGACCCATGTGG;GGAAG	3	2.9404738	2.4562303	0.0417235	0.4841764	0.4841764
MID2	4	999;1058;9027;70624	TGAGGCATTAAGAACCACGG;GCCTG	2	2.9317597	2.110462	0.042552	0.4884232	0.4884232
AFF1	4	1408;4259;5160;11283	CATGGGCCGGACATAAGCCG;TCTGA	3	2.9108175	1.9396397	0.0445666	0.5060463	0.4928511
ZC3H12A	4	326;2913;5166;11863	AACACGGGACAGCCACCGAG;GCGG	3	2.9093409	2.2490737	0.0447215	0.5024028	0.4928511
RMND5B	4	714;1088;7656;35260	GGGAGCTGTTGAGTTCCAGC;GTGGA	2	2.9078272	2.1689147	0.0448753	0.4988245	0.4928511
CHD4	4	417;1092;9115;42909	TGATGTTCCGAAACTATCAG;GCTATG	2	2.9046869	2.2815153	0.0451913	0.4971045	0.4928511
NNAT	4	1741;2219;5188;26639	GATGATGAGCAGTTCAGCCG;TCGGC	3	2.9039416	2.0890934	0.0452714	0.4928511	0.4928511
MCIDAS	4	120;4710;5237;71016	TCAGAACCATTCCCACCAAA;GGGCG	3	2.8920001	2.2549918	0.0465416	0.5015098	0.5015098
CSK	4	1030;1140;7980;27028	GCTCCCGTGTGATCTTGCCG;GTACA	2	2.8678892	2.072165	0.0490635	0.5233436	0.5216448
C20orf144	4	430;1144;11495;20322	CGCTGGGCTGGGCTCCCCGG;GTGG	2	2.8648941	2.2550667	0.0493982	0.5216448	0.5216448
ENOPH1	4	341;5376;5386;49644	ACTGCACTCAAACAGCTGCA;GCAGA	3	2.8563815	2.0576935	0.0503275	0.5261964	0.5261964
TMEM139	4	232;1170;32014;48566	TGTGAGAAAGAAATAAGCAA;ACTCAC	2	2.8456815	2.377716	0.051552	0.5337151	0.5337151
SOBP	4	53;4566;5463;52044	GCCAAAACCAAAATTACCCG;GCTGG	3	2.8383698	2.3634215	0.0524346	0.5375816	0.534099
C7orf61	4	3861;5047;5476;65792	GGTCATGAAGTTCTTCCGAT;GCCAAG	3	2.8353546	1.7373471	0.0528161	0.5362862	0.534099
POU1F1	4	4631;5457;5502;64928	GCAGAGGCGTCAGAATTCAG;GTTGG	3	2.8293464	1.6928626	0.0535416	0.5384759	0.534099
EFNB1	4	4442;5383;5521;66063	TCTGCCCCCGAGCAGAAGCA;ATTGC	3	2.8249742	1.699911	0.0540312	0.5382735	0.534099
ARFGEF2	4	476;1200;13127;53207	CCACACATTGAAATTCATGA;ATGTAG	2	2.8240453	2.2129768	0.0541466	0.5343817	0.534099
TMEM244	4	5145;5498;5542;27074	ATAGTTTATGTTGAGCCATG;TAGTTTA	3	2.8201597	1.675766	0.0546238	0.534099	0.534099
CLCA4	4	3228;4088;5642;35179	TGAGTGGGCTCACCTCCGGT;GGAAC	3	2.7974886	1.8038381	0.0572755	0.5548895	0.5548895
SFPQ	4	485;1256;39863;64066	TCTACCTGCTGATATCACGG;GGAAGT	2	2.7850906	2.1895111	0.0588628	0.5650833	0.5650833
NOL4L	4	533;1634;5743;7756	GCAGAACCTCCACAGTCAAG;TGGCA	3	2.7750071	2.296615	0.0602672	0.573353	0.5663647
TMEM189	4	423;4578;5756;9835	CCAACACCGAGTATGACGAG;ACCAA	3	2.7721431	2.0421058	0.0606591	0.5719287	0.5663647
TENM3	4	939;1282;69669;70207	TCATGGTGTGTGTATCCACG;TCAAAC	2	2.7676015	2.0413026	0.0612755	0.572628	0.5663647
SNTA1	4	1072;4457;5781;15554	TCAAGACACCCTCTTCCTGA;GGTGC	3	2.7666541	1.9167262	0.0613868	0.5686352	0.5663647
CREBBP	4	1158;1290;15764;22542	TGGACCTCTCCACCATCAAG;GCAGC	2	2.7622928	1.9950642	0.0620219	0.5695228	0.5663647
TBPL2	4	272;1295;28350;69777	GGACTGAGTCAGTATCACCA;GGTAC	2	2.7589918	2.3001808	0.0624928	0.5688999	0.5663647
BCOR	4	1671;4180;5829;8411	GTGCAGACTGGAGAATACAG;AGTTAC	3	2.7561835	1.8699616	0.0628618	0.5673681	0.5663647
RNF112	4	287;3640;5842;51869	CCTTCTTGCTGGGGAAAGAA;GATGTT	3	2.753363	2.153739	0.063287	0.5663647	0.5663647
CPTP	4	295;1314;31090;45981	CCACGAGCTGAGCAACCGGC;CCCG	2	2.7465655	2.2765424	0.0642048	0.5697506	0.5697506
UBE2NL	4	627;1320;29612;32273	GATGATCCTGTGGGGCAGCT;ACTTG	2	2.7426794	2.1137904	0.0648119	0.5703449	0.5699439
SYCP2	4	793;2691;5923;18672	TATACCAGATTCACAGGCAG;GCATGC	3	2.7359337	2.0873793	0.0657672	0.5739685	0.5699439
ATP2C1	4	89;4901;5956;47889	GGTCAGATGTGGCAAAGCAA;TGATG	3	2.7289033	2.2329915	0.0667766	0.5780005	0.5699439
MYLK2	4	2490;3579;5957;11325	GCTAGTTGAGGGTTGGGCCA;GGTAC	3	2.7286909	1.8504409	0.0668265	0.5737297	0.5699439
PROCR	4	2088;5586;5961;35654	GGAGCATATGAAGTCTTTGG;GCTCAC	3	2.7278416	1.7526325	0.0669252	0.5699439	0.5699439
CXCL14	4	525;1365;9488;54768	GCACTCACCGTCCACACGCG;GGAGC	2	2.7140949	2.1371557	0.0689834	0.5827721	0.5827721
C20orf173	4	2275;2421;6043;34407	AGGTGTCAGATCCAGATAGG;AGCAG	3	2.7105597	1.9646793	0.0695676	0.5830431	0.5830431
ARNTL2	4	4013;5702;6143;59491	GTCTTGTCCCTGGACTCACG;GAGTTT	3	2.6898091	1.651582	0.0728764	0.6059642	0.6059642
TSC1	4	1360;1453;6206;22452	ACAGTCTCCCAGGTTCTCAG;ACTCC	3	2.6769147	2.1664587	0.0749689	0.6184934	0.6177068
L3MBTL1	4	2016;5741;6219;29346	AGATGGGTCAAGGACCCGTA;GTGCG	3	2.6742708	1.7321508	0.0754585	0.6177068	0.6177068
RELB	4	2143;3739;6256;25698	GCAGGGACAGATGCGCCGGA;CTACC	3	2.666777	1.837436	0.0767267	0.6232568	0.6225869
TLDC2	4	3623;4174;6283;9019	GTCTTCTGCACGTCAAGGGA;GAGTG	3	2.6613374	1.7384752	0.0776415	0.6258731	0.6225869
BPIFB1	4	1276;4961;6288;10266	CGTCATGTGGAACTCCACGA;ACAGA	3	2.6603327	1.8283398	0.0778234	0.6225869	0.6225869
RBM38	4	5401;5738;6344;43989	ACACTACCGACGCCTCGCTC;GTACTT	3	2.6491365	1.6019659	0.0797974	0.6335792	0.6335792
COX4I2	4	864;3987;6392;19670	GCTGACCCACGCCGAAAAGG;AAGGT	3	2.6396209	1.9347165	0.0816134	0.6431622	0.6431622
RAB40C	4	215;1503;45511;60144	TGGTGATGTCATACACCAAG;GGCTG	2	2.6320786	2.2872906	0.0830282	0.6494647	0.6459968
HIST1H1B	4	3188;4072;6435;16783	GCAGTTTCTTGCCACCATGT;AGTTGC	3	2.631159	1.7510267	0.0831966	0.6459968	0.6459968
EPPIN	4	1077;2005;6498;12595	AGAATGTGAATTCCAAGAAA;GTCCTC	3	2.6188666	2.088169	0.0854273	0.6537047	0.6535427
EPPIN-WFI	4	1077;2005;6498;12595	AGAATGTGAATTCCAAGAAA;GTCCTC	3	2.6188666	2.088169	0.0854273	0.6537047	0.6535427
TREX2	4	1115;1532;54004;56577	TCCTGGACCTGGAAGCCACT;GGAGC	2	2.6158233	1.9296654	0.086025	0.6535427	0.6535427
C17orf49	4	4175;4962;6544;28275	ATATACCTTGCGTTTCACAG;GGTCGG	3	2.6099689	1.6573939	0.0871591	0.6574289	0.6574289
SYAP1	4	303;1556;14263;56745	ATCCTCCTGGAGCATGACCA;GGAGC	2	2.6026066	2.1988227	0.088604	0.6635877	0.6615455
MAP1LC3A	4	2551;4310;6594;12261	ACCGGCCTTTCAAGCAGCGG;TCTGC	3	2.6003708	1.7537123	0.0890614	0.6623159	0.6615455
KARS	4	2211;6324;6607;46301	GCTGCCATCCACTTTCACCT;TATATGT	3	2.5978876	1.6689464	0.0895843	0.6615455	0.6615455
TGM2	4	926;5240;6672;16983	AGTTGTAGTTGGTCACGACG;ACGCT	3	2.5855473	1.8330784	0.0920781	0.6752391	0.6752391
IQCJ-SCHI	4	228;1598;8661;33578	GCAAATCTTCATCGTCGCCG;CTCCC	2	2.5799712	2.2486024	0.0932579	0.6791747	0.6753464
FANK1	4	1139;1608;70383;73336	CTGGGCAGCAACCATCAGAG;GGTGC	2	2.5746715	1.9046776	0.0944273	0.6829813	0.6753464
PSG2	4	2655;3690;6733;64875	CAGCCACCAAAAGTTTCCGA;CAGTT	3	2.5740792	1.7822181	0.09455	0.6792163	0.6753464
SHROOM4	4	1474;1610;47299;69909	ACAAGCTGAAGGCTTCAGAA;GCCCA	2	2.5736156	1.8511368	0.0946508	0.6753464	0.6753464

PRC1	4	74;2097;6754;51200	GGAGCTAATTGGGATTCCAG;TTAGCT	3	2.5701561	2.4410931	0.0953941	0.6760815	0.6760815
GALE	4	1192;1625;7158;28650	CCAGATCATGAAGGCCCACG;CCTGC	2	2.565739	2.092888	0.0962974	0.6779337	0.6771806
SPAG4	4	59;6204;6791;23849	AGAAAGCTCTTGAACACCCG;GTTGG	3	2.5632747	2.174319	0.0968317	0.6771806	0.6771806
NCOA3	4	1456;2778;6848;54091	CATGTGATACTCCAGGACAA;GTAGAG	3	2.5527494	1.933407	0.099208	0.6892345	0.6892345
ZSWIM3	4	1603;2173;6883;30425	AATCAAACAGGAAGAGAACG;TCACA	3	2.5463314	1.9869056	0.1006384	0.694602	0.694602
LIMK1	4	5356;6632;6933;44554	TCTCCGGCTTATACTCCCAG;GCACTG	3	2.5372215	1.5274461	0.1027662	0.7046826	0.7003695
TMEM254	4	3549;5785;6949;17197	AGGTGTTGCAGCCATGGCTA;GGCGC	3	2.5343208	1.6108628	0.1034471	0.704775	0.7003695
CDK15	4	1473;1693;39451;58425	AGTTCAAGAGTAAAAGGCCA;CATGA	2	2.5309406	1.8299377	0.1042465	0.7056684	0.7003695
ALKBH6	4	1842;6170;6970;38671	CTCCACTCTGAACGGTTCCA;GGTGG	3	2.530524	1.6772057	0.1043525	0.7018869	0.7003695
PDGFD	4	1395;1698;54521;57520	GTACCATTATTAGAGGACGA;TTCACT	2	2.5284386	1.8398074	0.1047901	0.7003695	0.7003695
RAD17	4	2226;4771;7034;72887	TCATATTCTGATAAATGAGA;TCATAAT	3	2.5190259	1.7168549	0.1070084	0.7106975	0.7106975
CLRN2	4	2937;5627;7052;34346	GCCCATGCTGACCAGAGCCA;TTTACT	3	2.5158117	1.635728	0.1077371	0.711065	0.711065
OGFRL1	4	2953;6027;7079;19031	TCAATGGGCAACCTGCTCGG;ATAAA	3	2.5110062	1.6152355	0.1089357	0.7145097	0.712686
NLRP14	4	1454;1746;35516;52004	TCACCAATAAGGATGCCTCA;GCCTTC	2	2.5047969	1.819516	0.1103889	0.719572	0.712686
VSTM2L	4	1696;2331;7116;7900	CGAGTTGCAGGAAAAGTGCC;GATCT	3	2.5044519	1.9456444	0.1104315	0.7154336	0.712686
GGT7	4	3259;6588;7153;22305	TGACCGTGAGCCCATCCTGG;TGAGC	3	2.4979329	1.5750678	0.1121093	0.7218743	0.712686
MLLT10	4	863;2448;7155;13181	GCCCGTCGCAATAAACCAGC;TGCCC	3	2.4975815	2.0225202	0.1122153	0.7181779	0.712686
ARHGAP36	4	1186;1762;26400;72132	GATTGCGAAACAAATCCCCC;GCCTG	2	2.4970641	1.8575099	0.1123213	0.714526	0.712686
LCE3C	4	483;1765;53150;72527	GGTGAGGGACAACTGGGAGG;TGACT	2	2.4956222	2.0456566	0.112707	0.712686	0.712686
RPL4	4	32;1792;52766;57311	TGTTCACTCTACGATGCCAA;GCTAAT	2	2.4827573	2.6246754	0.1160822	0.7296598	0.7296598
BPIFA3	4	5292;5849;7288;14799	CATTCAGTCTGTCCCCAAAG;CTATGA	3	2.4744415	1.5409221	0.1183089	0.7392553	0.7309142
CHD6	4	2798;2998;7296;27193	GCTAGCCACCTGCAACCCCG;CGATA	3	2.4730637	1.7991364	0.1186872	0.7372572	0.7309142
HOXC10	4	2887;3232;7299;31567	TGTAACTCCGAACTCGTACG;TCGTCC	3	2.4725474	1.7741561	0.1188037	0.7336647	0.7309142
NECAB3	4	1759;3824;7325;37896	CCCCAGGCTGAGAACCCCAT;TTAGA	3	2.4680822	1.7913358	0.1199658	0.7365343	0.7309142
REM2	4	1805;2509;7327;21378	GCTCACGAACCGGAGAACCC;CTGCC	3	2.4677394	1.9044464	0.1200625	0.732867	0.7309142
RAC3	4	1774;1830;7631;52959	TTTCCCGTCCACCATCACGT;GTACTC	2	2.4649841	1.7592678	0.1208463	0.7334119	0.7309142
CXCL10	4	3123;3245;7363;42371	CTTATCTTTCTGACTCTAAG;GATAAG	3	2.4615858	1.7594474	0.1217444	0.7346405	0.7309142
NCOA6	4	3866;4425;7376;8558	GGCAAGACCATCCTTAGCCA;GCACA	3	2.4593713	1.6473682	0.1223463	0.7340777	0.7309142
MLLT1	4	876;2443;7402;21290	GCAAGAAGCCCACCACGGAG;GCCCT	3	2.4549546	2.0067952	0.1235001	0.7368143	0.7309142
RRAGA	4	1669;5343;7414;16589	TTATTACCAGTCGTGTCTGG;TTTGATT	3	2.4529215	1.7027787	0.1241082	0.7362826	0.7309142
AQR	4	3286;5408;7450;54448	TACGGTGTCCCAAATCAATG;GTAACA	3	2.4468429	1.6091783	0.1258265	0.7423063	0.7309142
FAM209B	4	3497;5656;7462;46610	AGAAAACTAGCGAACCCCAG;CCACA	3	2.4448235	1.5888376	0.1264076	0.7415913	0.7309142
KRTAP4-2	5	1198;1489;27664;37446;5	GGCTCTGTGTGCTCTGACCA;GCAGG	2	2.426938	1.7754235	0.1290448	0.7446521	0.7309142
KRTAP4-4	5	1198;1489;37446;61392;6	GGCTCTGTGTGCTCTGACCA;GCAGG	2	2.426938	1.7754235	0.1290448	0.7446521	0.7309142
KRTAP4-12	5	1198;1489;36814;37446;4	GGCTCTGTGTGCTCTGACCA;GCAGG	2	2.426938	1.7754235	0.1290448	0.7446521	0.7309142
IMPA2	4	90;1922;26457;41635	CCTGGAAGCACTCCTCCCAG;AGTGA	2	2.423477	2.3709952	0.1295417	0.7434568	0.7309142
AKR1C1	4	585;1923;22322;54452	CAGTGGATCTCTGTGCCACA;AATGA	2	2.4230371	1.9686558	0.1296051	0.7398001	0.7309142
DRAP1	4	493;1925;48363;69458	GGACGGGGAAGACAACCACA;AGAA	2	2.4221581	2.0045624	0.1297465	0.7366253	0.7309142
TRIM72	4	1519;1928;16315;16426	GCCTCACTCGGCTCGCACCG;ACACT	2	2.4208413	1.76862	0.1299471	0.733819	0.7309142
B3GALT1	4	735;1931;33580;59751	ACTCAAGTACCAGAGAGCGC;ATTCTT	2	2.4195266	1.9186548	0.1301249	0.7309142	0.7309142
OR11H1	5	333;1520;8915;45522;720	ACACCACAGGACAAAAGCAA;ACATG	2	2.4095922	2.0345928	0.1315292	0.7348935	0.734469
GNB2L1	4	1614;1963;7880;69625	CATCATGTGGAAACTGACCA;TTAGTC	2	2.4056327	1.7486892	0.1321488	0.734469	0.734469
SYNGR2	4	1516;1984;16105;41829	GTAGCCCTCACCATAGATGC;GTACAT	2	2.3966408	1.7569224	0.1335448	0.7383421	0.7376185
TATDN2	4	1933;1995;12825;28452	GCCTGATTCGGAACACTCGG;CCACA	2	2.3919698	1.7055437	0.1342195	0.7382071	0.7376185
OSCAR	4	770;2003;33453;53976	GCAGCGGTAAATTCCCCCTT;GAAAA	2	2.3885893	1.8933928	0.134811	0.7376185	0.7376185
LATS1	4	1101;2033;12870;39233	AAGAGGAGTGAAAAGCCAGA;TGATG	2	2.376035	1.8123911	0.1367527	0.7443861	0.7443861
ZNF33B	4	394;2049;40105;43651	AGAAGAACCATGGAGACTGG;AGGTA	2	2.3694171	2.0259986	0.137812	0.7463049	0.7463049
HK2	4	1310;2069;24267;50667	CATCAAGGAGAACAAAGGCG;GTCTA	2	2.3612193	1.7690941	0.1392205	0.7500861	0.7495772
MAPK8	4	260;2078;23954;71133	ATTCTGCTGGAATTATTCAT;GCATGAG	2	2.3575568	2.109156	0.1398359	0.7495772	0.7495772
UBXN2A	4	976;2099;35911;59539	TACCTGTTTCTTCTGTTCAG;TCAAAA	2	2.3490743	1.8239738	0.1412527	0.753348	0.753348
SPTY2D1	4	1874;2142;12971;27539	TCGTGGCCCTCACGAAGTGA;TCCCA	2	2.3319749	1.6817493	0.1442029	0.7652171	0.7652171
TFF1	4	356;2160;27901;28417	CACAATTCTGTCTTTCACGG;CCACAA	2	2.3249219	2.025441	0.1455251	0.7683725	0.7683725
STX16	4	1721;2191;8836;12457	TTAGATCCAGAAGCAGCGAT;GCTGGT	2	2.312916	1.6893468	0.1476758	0.7758491	0.7737744
SLC39A6	4	1970;2196;18837;74541	GTAATGAATAGGTCTTTGGA;AAGGGT	2	2.310996	1.661271	0.1480137	0.7737744	0.7737744
ZMYND12	4	1579;2222;18182;19332	CATAGAATATATTAGCCAGG;CATGCC	2	2.3010841	1.7008637	0.1499212	0.7798854	0.7744241
CCDC102B	4	733;2224;35781;44149	GTATTACAGGTATCCCTGGG;GCAGTC	2	2.3003266	1.8596289	0.1500636	0.7767996	0.7744241
TECPR1	4	884;2240;28340;50165	TGATCTGAACCACCTCCCCG;ACGATT	2	2.294292	1.8172689	0.1512153	0.7789434	0.7744241
LRRC37A3	4	1633;2245;23792;27833	CGCTAGTGCCGGGCACTGAG;TGGGC	2	2.2924153	1.6897086	0.1515677	0.7769685	0.7744241
TSPAN18	4	1555;2248;23214;52538	CACTGGCCCGAGCAGCTGGG;ACGAT	2	2.2912913	1.6990793	0.1518047	0.7744241	0.7744241
GPR158	4	299;2282;39645;61794	CTTGGCGCAGAAACTCGCCG;GCAGC	2	2.2786604	2.0397003	0.1544284	0.7840213	0.7820631
HPSE2	4	1502;2305;22442;46333	AGCCAAGTAGAGAGCCCCCG;GCAGC	2	2.2702258	1.6956048	0.1561623	0.7890307	0.7820631
ELMO2	4	823;2320;14587;48903	TCTGTACCAGAAGATAGCCG;AGGAC	2	2.2647717	1.8174967	0.1573952	0.7914729	0.7820631
TOX3	4	153;2328;29538;36516	GGATCGCTGAGGGCTTGAAA;ACAAG	2	2.2618777	2.1755215	0.1579877	0.7906872	0.7820631
BAX	4	104;2330;7604;25040	TCTGACGGCAACTTCAACTG;AACTCA	2	2.2611559	2.2585615	0.1581343	0.7876876	0.7820631
MLN	4	1810;2344;29386;29590	TCTGCCCCTAGGAAAAGGAA;TGCCAT	2	2.2561206	1.6507953	0.159233	0.7894369	0.7820631
AMPD3	4	1127;2349;24199;45163	GGTGGACACACACATCCATG;GGAGG	2	2.2543298	1.7467004	0.1595875	0.7874971	0.7820631
ABHD16B	4	856;2352;18829;21789	CGGCGCGCACGTCATCCCAG;CAGCA	2	2.2532572	1.8034936	0.1598027	0.7848912	0.7820631
SUPT20H	4	187;2371;11659;31593	AGGAGATCAACCAAAATAGG;TTAGTG	2	2.2464969	2.1245533	0.1612528	0.7883469	0.7820631
SPATA31A6	4	1542;2376;15400;19426	TGAGGAAACAACTAGAAGGT;GTGGA	2	2.2447272	1.6775045	0.1616187	0.7864945	0.7820631
UFSP1	4	1521;2384;15034;62912	GCTCACTTCGGAGGGCCCCA;GGGG	2	2.2419037	1.6788833	0.1622247	0.7858224	0.7820631
ZBP1	4	480;2401;11041;18127	GCAGGATTCTTTGTTCAAGG;AGTCCC	2	2.2359361	1.9171402	0.1635293	0.7885247	0.7820631
FGD4	4	891;2407;22682;41262	GCTCCCCCTAGAAGAAAGAG;ACTATC	2	2.2338403	1.7853921	0.1640064	0.7872308	0.7820631
GAP43	4	953;2431;32402;56180	TCTTCTCATACAGCACAGCA;ACTCCT	2	2.2255111	1.7671674	0.1659388	0.7929023	0.7820631
PEX11A	4	100;2432;39757;40439	TATTTGTTAGAGCCCAAAGC;TCTTCTC	2	2.2251659	2.2490483	0.1660178	0.7897065	0.7820631
ERAP2	4	1900;2437;22123;56276	GGATGCATAGATGGACACCT;AGTTAG	2	2.223442	1.6247235	0.1663973	0.7879619	0.7820631
ZKSCAN8	4	1250;2439;26459;41604	AGTTGGATCAGAGCTTCTCG;GGAGG	2	2.2227536	1.7095094	0.1665584	0.7852038	0.7820631

HOXD9	4	1440;2445;29595;35009	TCTGGGGGCAAAACTACACG;GTTCT	2	2.2206916	1.6794398	0.1670553	0.7840461	0.7820631
CXorf23	4	1464;2449;14175;24587	ACAAGCAAAGACATTCACAC;TTAGAG	2	2.2193198	1.6753781	0.1673734	0.7820631	0.7820631
SERPIND1	4	932;2463;20338;53604	TTTCCTTGTGGAAGTCGGCA;TCTCC	2	2.2145369	1.7663333	0.1684825	0.7837776	0.7829099
EIF4A1	4	1972;2473;22756;61020	TGAGGTGCAGAAACTGCAGA;GGTTA	2	2.2111378	1.6111395	0.1693058	0.7841531	0.7829099
TMEM145	4	1950;2478;41120;53804	GTGCTCACCAAACTCATCAG;TGCTTC	2	2.2094435	1.6125314	0.1697788	0.7829099	0.7829099
ZNF823	4	1319;2494;8108;35012	AAGACATGAAAGAATCCACA;CCACAT	2	2.2040457	1.6891005	0.1710875	0.7855148	0.7852411
MYL1	4	2054;2502;58627;70114	CACAAATCCCACCAATGCAG;GATGG	2	2.2013603	1.598139	0.1717715	0.7852411	0.7852411
GDF5	4	2520;2536;23942;24595	CTTCCTGCTGAAGAAGGCCA;GTGTT	2	2.1900456	1.5522746	0.1746384	0.794906	0.7920499
ADAM17	4	312;2545;21653;32903	GTAGACAGAGAACCACCTGA;CTTTA	2	2.1870769	1.9847807	0.1753536	0.7947357	0.7920499
DLL4	4	1703;2548;11177;52224	GGAAGTGCTTAAGGCAGACG;CTGCC	2	2.1860897	1.6280561	0.1756073	0.792484	0.7920499
ADA	4	1515;2556;8294;9418	TCCCAGCTAACACAGCAGAG;ATAGC	2	2.1834632	1.6504679	0.1762611	0.7920499	0.7920499
SLC8B1	4	2081;2584;31158;58317	CGTAGAGAGTGACAGCCAGA;GGATA	2	2.1743371	1.5820338	0.1786769	0.7995034	0.7995034
NCBP2	4	1184;2597;14742;47325	CGTCCCATTTATGTACCGCA;CTTCTC	2	2.1701349	1.694394	0.179732	0.8008312	0.8008312
TMEM51	4	758;2635;17529;30714	TGTGTCTTCTTGCAGCCAGG;TCAGG	2	2.1579755	1.7813909	0.1830033	0.8119811	0.8047969
TMEM53	4	916;2642;34579;67174	GGTGTAGTCCAGCTCTGCCG;TAATC	2	2.1557555	1.7405615	0.1835865	0.8111603	0.8047969
PLAGL2	4	1779;2647;11939;13066	CTTTGCATCTTGAATCCAGG;TGTGCC	2	2.1541735	1.603296	0.1840075	0.8096329	0.8047969
NAP1L4	4	979;2651;21742;50561	TAGAGAAGAGAATTTATCAC;TGGGGT	2	2.1529102	1.7252518	0.1843536	0.8077901	0.8047969
CXADR	4	55;2652;55833;66438	GGACTAAGCGTAAATTTGCA;AGATGA	2	2.1525946	2.3421884	0.1844326	0.8047969	0.8047969
CALU	4	508;2678;18665;44782	ACAAAAGCGCTGGATTTACG;TGAAC	2	2.1444341	1.8593238	0.1866998	0.8113373	0.8049023
C1orf198	4	500;2681;31887;45958	TCAATGCTGAGCGGTCCCGA;GATCA	2	2.1434978	1.8622322	0.1869638	0.8091549	0.8049023
COQ10A	4	135;2687;36291;50710	ACCAGAGACTTCTTACACCA;GCCAA	2	2.1416285	2.1424184	0.1875761	0.8084912	0.8049023
EML4	4	145;2694;36879;64190	ATAGCTGGCGTGGATAAAGA;ACATGC	2	2.1394531	2.125901	0.1882029	0.8078954	0.8049023
RASIP1	4	551;2698;17857;74120	GCGCGCACTGTGCAGTGACG;GCGC	2	2.1382126	1.8389468	0.1885376	0.8060556	0.8049023
CRCP	4	873;2707;34425;55611	GACTGCTGTGGAGATCCAGC;GAGTG	2	2.1354286	1.7404591	0.1893515	0.8062711	0.8049023
LYZL4	4	211;2712;16366;28394	GGAGAAGATGAAGGCATCCG;GGCTA	2	2.1338861	2.0422373	0.1897923	0.8049023	0.8049023
WDR70	4	349;2726;20857;73045	ATACCACTCACAGGGCTGAA;CATGA	2	2.1295827	1.9320224	0.190995	0.8067629	0.8067629
ZNF471	4	729;2753;46390;48271	GATCCTCACCAAGTGATACC;AATTCT	2	2.121348	1.7712925	0.1935449	0.8142766	0.8142766
KCTD15	4	1137;2771;49824;58302	GTGTGAAGCGAGGACCCGCT;GCTAC	2	2.1159047	1.6756581	0.1951561	0.8177971	0.8154202
ZNF540	4	354;2774;38167;38896	CTCTGATTCAGAGTAAAAGA;AGTGCC	2	2.1150011	1.9216865	0.195415	0.8156451	0.8154202
EMB	4	2024;2782;52498;54587	CGAGAGGAAAAGGAACAAAG;TTATT	2	2.1125964	1.556683	0.1961333	0.8154202	0.8154202
TRPC4	4	752;2808;11945;37621	TCGAGTGGATGATATTACCG;CTTCCC	2	2.1048306	1.7564913	0.1985771	0.8223429	0.8201121
NR1H2	4	199;2813;17806;44124	CAGTCACCTGTGGGGCCGCA;CGAG	2	2.1033457	2.0395769	0.1990272	0.8209873	0.8201121
ZKSCAN1	4	2430;2819;11683;62599	GGAAGATTCAGCATCACGCG;CTTCC	2	2.1015675	1.5151208	0.1995917	0.8201121	0.8201121
LY6G6C	4	2641;2838;28918;69177	AGACAGGGTGAGCAGCATAA;AGTGA	2	2.0959626	1.4961423	0.2013713	0.8242175	0.8242175
MLLT6	4	2179;2897;21957;35871	GCAGACCTGTTACATCTGCG;GTGTG	2	2.0788039	1.5251545	0.2069659	0.8438456	0.8327842
HLA-G	4	1018;2898;40310;63198	TAGCCCATGGCGATGAAGCG;ACAGC	2	2.0785162	1.6799171	0.2070636	0.8409969	0.8327842
ACD	4	2422;2901;18524;74575	TCCTCTTCCGAGGAAAGGCG;GCTTC	2	2.0776538	1.5038079	0.2073557	0.8389565	0.8327842
NCEH1	4	2883;2911;24251;68305	CCAGAAATGCAAATTCTGCC;TGGTGT	2	2.0747859	1.4687077	0.208336	0.8397053	0.8327842
ZNF114	4	369;2919;66433;67344	CTTGGGGTGGAACATTCAGT;AGGAG	2	2.0724989	1.8915553	0.2090792	0.8394968	0.8327842
RORB	4	589;2920;36811;54378	ACAATGGGCAGTTAGCACCA;GCTGC	2	2.0722135	1.7917994	0.2091686	0.8366745	0.8327842
RPTOR	4	492;2924;20937;52186	GCAGAACCTGAGTTTGACAG;AGGTG	2	2.0710729	1.829452	0.2095584	0.8350706	0.8327842
EIF4A3	4	1484;2928;13013;48847	GTTGGCTGTGCAGATCCAGA;GGAAT	2	2.0699339	1.5979167	0.2099119	0.8333343	0.8327842
RAD21	4	806;2938;24575;34238	GAGTAGTTCGAATCTATCAC;GGTTAG	2	2.0670935	1.7230401	0.2108588	0.8339586	0.8327842
HOMER2	4	431;2943;20785;28455	GATGCCTGCGAGCAAGCAGG;GACAT	2	2.0656771	1.8549626	0.2113505	0.8327842	0.8327842
TRIM62	4	1850;2974;10870;67452	CTATCGGCGAGGCCTTCGAG;GCTGC	2	2.0569511	1.5468216	0.2144191	0.8417346	0.8417346
CA12	4	981;2988;31804;58167	GTTCCTTTAAGATCACCAGC;TGTTCT	2	2.0530414	1.674892	0.2157819	0.8439471	0.8437712
CELSR2	4	695;3013;37019;50003	GGTGGAATCCTACCAGCTGA;GTACG	2	2.0461073	1.7437441	0.2182622	0.8504977	0.8437712
DNTTIP2	4	2358;3020;12866;63531	GTAGATGGTTCAGTCCCCTT;GATTAA	2	2.0441765	1.4923077	0.2189077	0.849877	0.8437712
ZNF749	4	64;3024;13792;44239	ATGGAACACACCCTGAGCAA;ATTGA	2	2.0430753	2.2545986	0.2192695	0.8481632	0.8437712
KMT2C	4	2721;3031;8709;10978	AGAGGTGCAGGTGTCAACAA;CTAGT	2	2.0411518	1.4629806	0.2199316	0.8476197	0.8437712
OR8H1	4	1380;3043;29989;47979	TTACTATGCTGGGCAATGTG;ATAATA	2	2.0378653	1.5967349	0.2211354	0.8491598	0.8437712
C16orf52	4	2715;3053;27479;29522	CAGTGTGGTGACCCACTCCG;CCCG	2	2.0351368	1.4603982	0.2222009	0.8501598	0.8437712
PXMP4	4	1771;3058;8981;26072	GTTCCGGAAGCCCTTAAGCA;ACGAG	2	2.0337761	1.5440045	0.2226842	0.8489334	0.8437712
RBM41	4	2129;3075;28035;65326	CCAGCTGCTTCCTTCCCAAA;GTTTCA	2	2.029167	1.5049277	0.2244067	0.8524225	0.8437712
TEX261	4	2485;3077;27373;73257	GCTGTCGCTCTTCATCCAGG;GGAGG	2	2.0286265	1.4742862	0.2246052	0.8501187	0.8437712
FAM49A	4	446;3079;35339;39522	CTTCAGCTCATCGAATCGAA;GGATGC	2	2.0280863	1.8288701	0.22481	0.8478549	0.8437712
GRAMD2	4	1887;3082;59819;72598	CACTGGCCAGAAGGCTTGAA;GGTCC	2	2.0272768	1.5280154	0.2251063	0.8459509	0.8437712
RFPL3	4	1748;3086;46928;54550	GACGGTGCATCCACACTCCA;GGAAC	2	2.0261988	1.542849	0.2255023	0.8444342	0.8437712
FCAMR	4	1844;3099;34802;35627	TTTGCAGGTCACCAGCAGGA;GGTGC	2	2.0227052	1.5303504	0.22681	0.8463299	0.8437712
TMA7	4	2174;3100;10295;41840	TTCCGGAACACTTACCTTCG;CCTCAC	2	2.0224371	1.497425	0.2269233	0.8437712	0.8437712
PPIP5K2	4	1391;3122;26813;55978	AGGATTTCCACTGGACAAAG;GATGG	2	2.0165617	1.5844569	0.2292736	0.8495191	0.8445057
RAB21	4	795;3124;22713;36463	AGTAAATTGGACCCAATGCA;GTTCAC	2	2.0160297	1.7003952	0.2294639	0.8472512	0.8445057
CDK10	4	2994;3130;55562;66653	GCTCACAGTAACCCATCACC;AACTTC	2	2.0144359	1.4313369	0.2300844	0.8465825	0.8445057
DFFA	4	1161;3134;13622;28761	GATGTAGATGAAACAGACAG;GCCATC	2	2.0133751	1.6200701	0.2304524	0.8449922	0.8445057
C7orf69	4	867;3154;18712;73836	ACATTTCCACATGACTTGGG;AGGCAC	2	2.0080926	1.6782357	0.2324233	0.84927	0.8445057
DTD2	4	512;3158;22558;27541	GCTGGCTTAAGCCATGGCTG;CTGGG	2	2.0070403	1.7889588	0.232941	0.8482265	0.8445057
SMAD3	4	2195;3168;23729;30674	CCACCAGATGAACCACAGCA;GTTCA	2	2.0044156	1.4865154	0.2338942	0.8487708	0.8445057
ARL8B	4	2394;3171;59387;62288	CTTCCTTCCAGAAGAGCGAA;TTAAAG	2	2.0036299	1.4690685	0.234205	0.846988	0.8445057
CACNG6	4	603;3172;8235;19336	TGTGGAAGGCGTGCACCAAG;CTCAC	2	2.0033682	1.7523988	0.2343183	0.8445057	0.8445057
ANKRD35	4	2162;3198;9565;69289	TGAGCCACGGAGCTGACGCG;GCTG	2	1.9965934	1.4855973	0.237046	0.8514305	0.848935
TAF6L	4	1039;3199;7743;17928	TGAGCCGGACAGACTCCCGA;TCATG	2	1.996334	1.6345778	0.2371551	0.848935	0.848935
TREML2	4	2995;3234;66164;74436	CAAGGGGTACAGGATCCCAG;TAAGG	2	1.9873078	1.4177095	0.2409098	0.859462	0.859462
GNAO1	4	1469;3250;9058;21996	TTACTTCATCTGCTTCACAA;TCTAGG	2	1.9832157	1.5566302	0.2425054	0.8622416	0.8622416
NAA20	4	294;3266;51777;53616	AATGAAATACTCTGGCCAGT;GCAGGT	2	1.9791446	1.8935606	0.2442425	0.8655035	0.8632032
CCT6B	4	2535;3271;31432;43048	ACTGGCTGATGTCTTAACAG;GATAAA	2	1.9778767	1.4450368	0.2447757	0.8644922	0.8632032
PIWIL1	4	2603;3280;51256;70972	TTATCGTTAGCAGAGAGAGG;GTTGA	2	1.9755995	1.4387644	0.2456822	0.8648012	0.8632032

TMIE	4	2329;3285;18502;59104	GCTGCCCGGCAACCCCCGCG;GGTC	2	1.9743373	1.4598141	0.2462487	0.8639157	0.8632032
RWDD3	4	3203;3291;10279;44317	TCAGAGTTAATCGAGATACC;TAACTC	2	1.9728253	1.3977809	0.246863	0.8632032	0.8632032
ADAMTS1	4	2304;3325;28133;59759	CGACACGGCCAGTAGCGCCG;AATG	2	1.9643115	1.4569197	0.2506635	0.8735994	0.8646762
B3GNT4	4	1573;3326;17111;68920	GGGAGCCCAGAAAGGCTGGT;GCAG	2	1.9640625	1.5331261	0.2507601	0.8710615	0.8646762
GOLGA6L6	4	247;3328;17716;66526	ATCAACATCAGGAAGCCCTA;CTGAC	2	1.9635647	1.9231843	0.2509628	0.8689074	0.8646762
CNN2	4	1529;3330;19741;33075	TGGCCTCGAACAGGTCCACA;AGCTC	2	1.9630672	1.5383971	0.2511655	0.8667674	0.8646762
SOX10	4	1694;3345;44407;54861	GCTGGTACTTGTAGTCCGGG;GCACC	2	1.959346	1.5157545	0.2527622	0.8694362	0.8646762
APOL6	4	2009;3356;36581;56656	GAAGACGTGGAGCTACAAGA;GAGG	2	1.9566283	1.4801798	0.2540148	0.8709079	0.8646762
TNIP2	4	3164;3360;23990;49750	GCAGCGCTACAACGCCAGCA;GCATG	2	1.9556423	1.3914944	0.2544608	0.8696135	0.8646762
SLC5A5	4	697;3370;18752;24245	GCAGACAGCGACAGGCCCAC;AATTA	2	1.9531829	1.6966755	0.2556645	0.8709088	0.8646762
GTF2A2	4	2236;3371;15751;54781	CTAGATGAGCTCATACAGGT;TCTGTA	2	1.9529374	1.4571262	0.2557695	0.8684649	0.8646762
APOBR	4	564;3374;55749;63050	TCAGCTGTAGAACAGACCTG;ATTTCT	2	1.9522012	1.7409916	0.2560938	0.8667791	0.8646762
SLC5A2	4	1049;3376;35711;72948	TGTGAGTCCGAGGAGCAGCG;TTGC	2	1.9517109	1.6102748	0.2562913	0.8646762	0.8646762
HEXDC	4	2077;3405;56069;59466	CTGCACCCTGAACCCCCACG;GTGC	2	1.9446348	1.4675645	0.2595034	0.8727248	0.870913
C1orf228	4	2755;3430;16302;23235	CTAGCCCAAGTATTTCCAAG;CTATCC	2	1.9385855	1.4093088	0.262389	0.879628	0.870913
HAO1	4	664;3436;19692;39704	TGTTACGCACATCATCCAGA;TCATTG	2	1.9371406	1.6988963	0.2630866	0.8791753	0.870913
FOXJ3	4	2476;3439;7921;11980	AGATGAATCCTACCTTTCCA;TATTATA	2	1.9364191	1.4288892	0.2634389	0.8775758	0.870913
NBEA	4	2316;3446;22399;63013	ATATTTGGCTATAAGCAGCG;AGTTCT	2	1.9347383	1.441113	0.2642362	0.8774637	0.870913
LOXL3	4	641;3456;44475;73510	CAGCTCGCTGTATCTCCACG;GAGCG	2	1.9323433	1.7039502	0.2653589	0.8784295	0.870913
GSTT1	4	237;3459;22963;65176	TGGCAAAGATGTAAACAGCG;ATGCC	2	1.9316262	1.9161019	0.2657071	0.8768336	0.870913
PDK3	4	2339;3462;49819;57100	ATATGAAAATAAGAGCCCTG;GAACCA	2	1.9309098	1.43726	0.266073	0.8753057	0.870913
MAGEC1	4	1014;3465;39401;50110	ACAACTCTGAGGACTCTCAG;AGGAC	2	1.930194	1.6065755	0.2664088	0.8736885	0.870913
CHFR	4	3163;3472;19292;22286	GGATGAGGTATGCTTCCACG;AAATG	2	1.9285264	1.377996	0.2672207	0.8736378	0.870913
NSL1	4	2541;3473;10508;57094	AAAGTCTTCTCGGGGAGTGG;ACAGT	2	1.9282885	1.4197836	0.2673485	0.8713581	0.870913
NYAP1	4	3379;3478;9873;49187	GCTTCTGCGGAGGAACCTTC;CAGAG	2	1.9270999	1.3649085	0.2680367	0.870913	0.870913
FAM209A	4	2276;3497;14296;16940	CCGTATTGCACCTTCCCCTG;AGAAA	2	1.9225996	1.4384669	0.270203	0.8752587	0.8724234
E2F5	4	2895;3498;25397;68275	ACCTCCAGTTGTGTACCAGA;ATAACT	2	1.9223634	1.3917034	0.2703142	0.8729413	0.8724234
CNRIP1	8	1313;3707;5024;15276;25	GGAGCCGGTGAGCAGCTTGA;TCTGT	3	1.9196123	1.3731211	0.2716822	0.8746841	0.8724234
FAM193B	4	764;3515;8548;68910	GCTCCGCCGGCGCCTCCGGA;GGAA	2	1.9183598	1.6599239	0.2722789	0.8739407	0.8724234
RASL12	4	1648;3518;27049;58925	AGAGGACACCTACAGCTCCG;GGCC	2	1.9176554	1.5004769	0.2726323	0.8724234	0.8724234
PTH1R	4	1513;3532;11711;50544	GGTCTCTTAGGCGGGCTGCA;CCTGG	2	1.9143766	1.5161937	0.2743059	0.875127	0.875127
BCCIP	4	2461;3543;16724;44225	GGAAAAGGATGAAGAGGACG;CGCTC	2	1.9118099	1.4177689	0.2756011	0.8766108	0.8766108
AKAP5	4	3382;3558;23213;48638	TCTTCTCTTGAAGCAAAGCA;GATCAG	2	1.9083235	1.355355	0.2773849	0.879635	0.879635
LTBP2	4	1659;3581;23794;44918	GCTGGAGCTGACTACCCAGG;GCAAG	2	1.9030077	1.4918066	0.2800315	0.885369	0.8800181
COBL	4	409;3586;46757;61726	CCAGCCCCATGAAAAGGCAA;TCTCC	2	1.9018568	1.7842365	0.280599	0.8845152	0.8800181
SLC16A11	4	647;3588;21839;59721	GAAGCGCCCAGGACACTGCG;GTGT	2	1.901397	1.6865068	0.2808392	0.8826374	0.8800181
CHIC1	4	646;3589;31266;50796	GATACCGACGACGACGACGA;CGGC	2	1.9011671	1.6867189	0.2809462	0.8803538	0.8800181
ACOT8	4	3290;3604;12536;63690	GCTGCCAGTACTGTACCAAG;AAGTG	2	1.8977277	1.355206	0.2826677	0.8831273	0.8800181
RTCB	4	1889;3606;16926;69467	TGGACTGGTCCTTAAGAGAA;AATGTG	2	1.8972703	1.4628	0.2828974	0.8812379	0.8800181
ABHD6	4	3192;3610;38567;64615	CCCTCCATCCTCATGCTCCA;GTATGC	2	1.8963562	1.3601932	0.2833392	0.8800181	0.8800181
ASPHD1	4	1544;3620;15558;39596	GGAAGGGAAACAGTCCAGCG;GTAG	2	1.8940757	1.5019151	0.2845699	0.8812489	0.8806675
UQCR11	4	1750;3643;23503;55894	TGACCAGCTCCCGGTAGCGT;CTCCT	2	1.8888557	1.473947	0.2872903	0.8870719	0.8806675
CCDC39	4	134;3645;19016;48734	ACTGGAAGTTCTATTCACAG;GTAGTC	2	1.8884034	2.0174116	0.2875294	0.8852217	0.8806675
SFTA3	4	3596;3675;35193;68613	CAGGGCAGACTTACACAAAG;AGATA	2	1.8816506	1.3306536	0.2909826	0.893249	0.8806675
BIRC7	4	2493;3676;11868;49043	GGTGGCACCTCAGCAGTCAG;CCAG	2	1.8814265	1.4000605	0.291097	0.8910099	0.8806675
FCRLB	4	3070;3679;43282;74242	GGACTCACCCAGGAGCAGAA;TCAAT	2	1.8807547	1.359744	0.2914556	0.8895292	0.8806675
PCDH9	4	3008;3680;21707;22476	GGTCCCATTTCATTTGAAGG;CCCTCC	2	1.8805308	1.3634986	0.2915544	0.8872663	0.8806675
OCRL	4	2243;3684;50482;51598	TGGTACAAAGAGCTTCCGCA;TTATTC	2	1.8796362	1.4198599	0.2920658	0.8862686	0.8806675
LRRC34	4	2022;3686;27267;29819	CTCCTCTCACCCACTGGCCG;CTGAG	2	1.8791892	1.4401762	0.2924223	0.884808	0.8806675
MSTO1	4	1801;3687;71758;73478	TGAATCATACAGATGCTCCG;AGATCC	2	1.8789658	1.4632199	0.2925502	0.8826657	0.8806675
TMEM102	4	886;3693;60246;67445	CTTCGTGGTGGGATTCGCGA;ACGTG	2	1.8776269	1.6084633	0.2931957	0.8820931	0.8806675
XAGE2	4	544;3701;25712;53786	ATAGCCCCAATCAGCTCAGG;TCTCTC	2	1.8758452	1.710478	0.2941147	0.882344	0.8806675
CDK9	4	1920;3709;38760;43868	CGAGTCGTACTGCTTTGCCA;CACGC	2	1.8740676	1.4479415	0.2951022	0.8827986	0.8806675
BRWD1	4	2783;3718;14008;66246	TATCGCCCGGTACCTATCGG;GTCTTA	2	1.8720727	1.3741102	0.2961822	0.8835266	0.8806675
CYB561	4	973;3730;31896;34849	GCGTGGCTCGGGCTGTACCG;GAAG	2	1.8694207	1.584789	0.2975512	0.8851102	0.8806675
C8orf88	4	2013;3740;13800;49225	TGTATGCACTGAGTGTTGCA;GGAGA	2	1.8672175	1.4350783	0.2988433	0.8864567	0.8806675
PDS5B	4	2470;3747;26281;39344	CAAATTTCACACATTCCAGG;TATCCA	2	1.8656791	1.3939919	0.2996625	0.8863966	0.8806675
CYP2C19	4	789;3756;42574;47712	TGGCCTTACCTGGATCCAGG;GCATG	2	1.8637055	1.6258252	0.3007259	0.8870574	0.8806675
TMEM106B	4	975;3766;41732;57709	GGAACAGGAAGAATTCCTAG;CTGAA	2	1.8615185	1.5804097	0.3019192	0.8880967	0.8806675
NRBP1	4	982;3775;36809;73086	ACCATCTTCATCCAGCACAA;GGATAC	2	1.8595554	1.5779366	0.3030253	0.8888741	0.8806675
CTGF	4	2869;3779;29226;48231	CGAGCCCAAGGACCAAACCG;GGAG	2	1.8586846	1.3615871	0.3034962	0.8877893	0.8806675
IGFBP3	4	1545;3780;64752;67974	TAGCGTTGACGCAGAGCCCG;ACTCT	2	1.858467	1.483979	0.3036011	0.8856431	0.8806675
ZNF155	4	3658;3787;11002;65018	ACAGGACAGAAACCTTCCCA;TGTTC	2	1.8569457	1.3151575	0.3044504	0.885674	0.8806675
SPATA9	4	679;3790;61708;68815	GAAAACATCCAAAATCAGGA;TGCCA	2	1.8562947	1.6537545	0.3048932	0.8845255	0.8806675
PSPH	4	2951;3791;30630;32208	TGGTCTCCCACTCAGAGCTG;GATGA	2	1.8560778	1.3549086	0.3049962	0.8823998	0.8806675
SIGMAR1	4	3663;3810;10922;20511	GCTGCTCCTGGCTGTCGCAG;GCTCC	2	1.8519682	1.312418	0.3072685	0.8865452	0.8806675
MDGA2	4	2356;3815;26766;27659	GCATCATCTGAGGTGATGGC;TGTCA	2	1.8508903	1.3958309	0.3079057	0.8859631	0.8806675
TTLL7	4	1171;3823;38776;59523	GGAACAGCAGAGACACCAGT;AATTC	2	1.8491689	1.5361932	0.30886	0.8862939	0.8806675
MAPK7	4	1068;3835;23044;62646	TCAAAGGCAAAGTCAAAGGG;GCTGG	2	1.8465938	1.5539865	0.3103704	0.8882144	0.8806675
C7orf55-LU	6	615;3910;7411;20742;275	CCATGACCTGGCTTTAAGAG;GCTCC	3	1.8452292	1.5496198	0.3112207	0.8882407	0.8806675
PDCD4	4	1372;3848;35067;63446	AATCTTGGTGAAATGAAAAG;GCAGTA	2	1.8438138	1.5009006	0.3120606	0.8882372	0.8806675
CAV3	4	284;3857;34127;44595	TCAAAGCTGTAGGTGCCCAC;CCAGC	2	1.841895	1.8323627	0.3131812	0.8890305	0.8806675
CLDN19	4	1053;3858;32099;38943	TAGGTCACATCCAATCAGCG;TAGTTG	2	1.8416821	1.5544693	0.3133215	0.8870443	0.8806675
RPS17L	4	486;3859;51093;73315	CACCAAAACCGTGAAGAAGG;AGCTC	2	1.8414693	1.7172619	0.3134442	0.8826588	0.8806675
RPS17	4	486;3859;51093;73315	CACCAAAACCGTGAAGAAGG;AGCTC	2	1.8414693	1.7172619	0.3134442	0.8826588	0.8806675
EVL	4	711;3860;51444;56945	ACAGAGTATCTGCCAAGCCC;GGTGT	2	1.8412565	1.6365172	0.313571	0.8806675	0.8806675

NSUN7	4	3650;3868;34580;73804	TTTCCCACCGGACAGAGGCA;AGACT	2	1.8395563	1.3068648	0.3145377	0.8810394	0.8810394
GPR82	4	2567;3884;16164;34624	TCAATCTGCTCAATGCAGAG;TTTGCA	2	1.836167	1.371747	0.3164962	0.8841798	0.8812777
DEFB129	4	1549;3885;16620;18352	TCACCTGGTACTGTAGCATG;CTACAG	2	1.8359557	1.4721976	0.3166178	0.8821857	0.8812777
ALX1	4	488;3896;36315;65098	GCTGCTGTCCTGACAGGGCG;TATCC	2	1.8336348	1.7124705	0.3180721	0.8839055	0.8812777
UTP23	4	2999;3899;40102;47496	GTCTCCCCCATGAGGTAGCG;GCTGC	2	1.8330031	1.3403036	0.3184359	0.8825939	0.8812777
KLC4	4	556;3902;17826;31302	ATTGAGTGTCTGCAGCAGGG;GGCTA	2	1.8323718	1.6841088	0.3187955	0.8812777	0.8812777
GADD45G	4	3908;3917;25392;42019	TGGCTCCTCCGCAGGATGCA;GCTGC	2	1.8292233	1.2892378	0.3206812	0.8841759	0.8826475
ZDHHC24	4	2381;3920;32377;58686	GAGCAGTGTCCGCTGCGTGG;AGTTG	2	1.8285951	1.3826164	0.3210409	0.8828624	0.8826475
MTX1	4	265;3939;10450;52749	GAAGAAGCCTCGCCTCCCCG;ACGTG	2	1.8246285	1.8385993	0.3235502	0.8874521	0.8826475
CCDC37	4	3952;3953;12153;22328	TCAAGCGGAGAGAGATCCAG;TCTTG	2	1.8217187	1.2834594	0.3253423	0.8900557	0.8826475
MDH2	4	454;3966;13425;42438	TCTCCTGAAGAACAGCCCCT;TGTGTG	2	1.8190266	1.7205499	0.3270076	0.8922999	0.8826475
ZBTB46	4	3222;3969;9490;20895	GCTGGACATCAGCATCAAGT;CGTTCC	2	1.8184067	1.3194605	0.3274203	0.8911233	0.8826475
GPR75-ASI	4	3279;3975;20471;24554	GCGGCCTCTCACCTGCCGGG;TAGTC	2	1.8171683	1.3155532	0.3282062	0.8909658	0.8826475
PWP2	4	710;3981;30926;57361	TTAACCTCATCCACTCCCTG;GCAGCT	2	1.8159319	1.6241517	0.3289785	0.8907726	0.8826475
STK38	4	3781;3986;9908;45567	GCTTGTTCAGAAGAAAGATA;GGAACT	2	1.8149031	1.2880838	0.3296136	0.8902098	0.8826475
TMEM263	4	3480;3990;9635;26353	GGTATCTTCAGTGTTACAAA;GATGCA	2	1.814081	1.3029269	0.3301376	0.8893502	0.8826475
ASCC3	4	3624;3992;60186;62849	TTCATTATAATTATCTTGCT;TTGCTTGC	2	1.8136703	1.2952358	0.3303943	0.8877771	0.8826475
DEFB121	4	1494;3998;15967;66281	CTGTTTACCTGGGGTGACCT;CTCCTC	2	1.8124396	1.4678002	0.3311615	0.88758	0.8826475
ATP4B	4	3311;4002;15955;29639	GTACTACGTGGCCTTCTACG;GCACG	2	1.8116201	1.3109622	0.3317831	0.8869948	0.8826475
CXXC4	4	226;4004;22921;45646	ACCACCGAAACGACTCCCAG;GCTGC	2	1.8112107	1.8661179	0.3320274	0.8854063	0.8826475
UBTD2	4	1650;4006;50802;56488	GTTCTCGTTGAGGCTGCCCG;TGGAC	2	1.8108015	1.4468044	0.3322924	0.8838812	0.8826475
PCDHGB2	4	3428;4009;48614;73929	CCTGTTGTCTTTGTTCCCCG;GCTCCT	2	1.8101882	1.3037741	0.3326833	0.8826974	0.8826475
FAM127B	5	871;3139;9377;31278;528	GTTCTCGTCCACGAACATGT;GTTCCT	2	1.8088499	1.5317424	0.3335003	0.8826475	0.8826475
MSMO1	4	2997;4032;16329;16739	TATTCAGCTTCCATTCCAAA;ATTGGG	2	1.805502	1.3266798	0.3357072	0.886267	0.8857842
SLC29A3	4	848;4037;13922;37672	GCAGTGATAAAGAAGTTCCA;GCTGC	2	1.804487	1.5810769	0.3363631	0.8857842	0.8857842
PLAC1	4	2966;4052;12349;68094	TGATGCCACATTCAGTAACA;TGAACC	2	1.80145	1.32663	0.3382498	0.8885369	0.8884431
TAAR1	4	1686;4057;45448;72225	GATCACACACAGCATAGTAG;TTCCAA	2	1.8004404	1.4372582	0.3390554	0.8884431	0.8884431
LIMD1	4	1971;4086;45231;50741	CGAGTTTGAGGAAACTCGCA;GTAGA	2	1.7946104	1.4029769	0.3428018	0.8960364	0.8910281
PINK1	4	2744;4106;21885;64676	CCCAGCCTGGACGCTCCCCG;CCTC	2	1.7906153	1.3360928	0.3455679	0.9010364	0.8910281
PSMA8	4	2629;4107;25560;51083	AGTTGAATATGCCCAGGAAG;ATTAAG	2	1.7904161	1.3442495	0.3456916	0.8991388	0.8910281
PCGF1	4	2150;4108;25809;61369	GCTCCAGTCAGTGTACAAGA;ATTGTT	2	1.7902169	1.3835099	0.3458309	0.897291	0.8910281
SUN2	4	1466;4109;32636;63781	AGGCACGTCAGCAAGAGCAG;TAGCC	2	1.7900178	1.4604485	0.3459484	0.8953958	0.8910281
PRKAR1A	4	4041;4118;21676;56240	GGAGAGACTGTGATTCAGCA;GCGTG	2	1.7882282	1.2626983	0.3471802	0.896387	0.8910281
SS18L1	4	1868;4123;12883;24072	CCTGGAGCCCTGACTCAGAG;GTACC	2	1.7872358	1.4100224	0.3478985	0.8960507	0.8910281
DLGAP4	4	3090;4126;19003;32927	CAGCTCTGAAAAGTGAACAA;GTCGG	2	1.7866409	1.311459	0.34828	0.8948508	0.8910281
SCAI	4	2133;4133;11866;47224	GGAAGTTCCAGCAGCAGCAT;GGAGC	2	1.7852547	1.3825999	0.3492478	0.8951593	0.8910281
SLC12A9	4	3639;4135;18507;39574	GCTGGCCTACCGGCTCCTGG;ACCTC	2	1.7848591	1.2800708	0.3495076	0.8936563	0.8910281
SGK223	4	2563;4142;13736;15278	TCACACCTTCATCTTCCAGG;TCTATG	2	1.7834761	1.3457041	0.3503891	0.8937462	0.8910281
DNAJC27	4	2137;4149;8890;27538	AGTCAATTCCAATTGTTGCC;CCTTTG	2	1.7820957	1.3806495	0.3513496	0.8940367	0.8910281
HOXA11	4	3493;4150;17972;64183	CAGTGGGCCAGATTGCCGCG;GCCAC	2	1.7818986	1.286145	0.3514785	0.8922147	0.8910281
CCDC30	4	1550;4153;9245;22777	ACTAAAATACAACCAGCAAG;ATGAGC	2	1.7813079	1.4447424	0.3518548	0.8910281	0.8910281
ABCA5	4	3346;4173;34784;67118	GAAATAGATACCTTTCCCCG;ATACAAT	2	1.7773813	1.2918789	0.3546251	0.895895	0.8926896
CDH19	4	665;4176;31338;31903	CGAGCATTATTACCACTTGA;GTACCA	2	1.7767941	1.618405	0.3550295	0.894776	0.8926896
DCAF8	4	1248;4189;10664;48372	ACCCAGCTCCCGCTCCCGAA;CCTCT	2	1.7742545	1.4855899	0.3568372	0.8971906	0.8926896
OR7G1	4	1122;4196;8820;58263	AAAGAGAAGGAAGTACATGG;TTAAAG	2	1.7728905	1.506902	0.3577862	0.8974401	0.8926896
PNMAL2	4	1096;4202;14317;70618	GCTGGTCACCGGCATCCCGG;CGACA	2	1.7717233	1.5111794	0.3586095	0.8973736	0.8926896
ROBO2	4	344;4204;58832;73492	AGGATGCTCCACAATCCGCG;GTAATT	2	1.7713346	1.755988	0.3588663	0.8958931	0.8926896
NUP54	4	3746;4206;17869;43979	AGGGGGATCACTCACCCGCG;GTATT	2	1.7709462	1.2678033	0.3591158	0.8944015	0.8926896
PHIP	4	1523;4207;19897;27801	AGGAAGTTACTCCCATCTGA;ATATGG	2	1.770752	1.4430399	0.3592738	0.8926896	0.8926896
KRBOX4	4	2609;4225;10784;23908	GCTTGAGAACTACAGCCACC;TGCCC	2	1.7672655	1.3341516	0.3618237	0.896915	0.8968171
TSGA13	4	2283;4231;16822;35061	GATGGACTGTGTAATGCCGA;ACAGT	2	1.7661068	1.3596166	0.3626334	0.8968171	0.8968171
PSKH1	4	162;4248;9405;32672	TCACTCTTAGTGCCACTGAA;GTACAA	2	1.7628334	1.9136663	0.3649536	0.9004463	0.9004463
FYN	4	2187;4255;30829;64446	CCGTGCTTCATAGTCATAAA;TTGTCC	2	1.7614896	1.3657735	0.3658954	0.9006656	0.9006656
KRTAP4-11	4	128;4299;28266;57166	GCCACAACAGGAGTTTACCA;GTGTG	2	1.7530967	1.9596533	0.3721688	0.9139773	0.9095068
COX6B2	4	2746;4300;30332;41932	GGGAAGCGCGGGTCGAAGGG;GGAA	2	1.752907	1.3170986	0.3722998	0.9121776	0.9095068
TNNC2	4	2220;4302;57542;70169	GCTGGACGCCATCATCGAGG;GCAGA	2	1.7525278	1.358337	0.3725482	0.9106734	0.9095068
TMX3	4	2946;4307;33017;65988	ACCTGTGGCGCAGAGCCGCA;CTACA	2	1.7515805	1.3029828	0.3732592	0.9103043	0.9095068
RPL13	4	167;4311;35058;40125	TCGTGTGGTACCGAACCGTG;ACTTC	2	1.7508236	1.9011043	0.3737935	0.9095068	0.9095068
IQCK	4	1201;4324;18480;48385	TTATATCCTTCTGGAAAGGG;GCGAGC	2	1.7483687	1.480566	0.3757021	0.9120491	0.9120491
PPP1R3D	4	2511;4348;16126;41785	ACAGGTCAAGGTGTTCAACG;CCAGG	2	1.7438574	1.3298761	0.3790274	0.9180114	0.9180114
ZNF616	4	631;4375;10803;69917	AAGAGATCAACATAGTCAAG;GATCAA	2	1.7388138	1.6105119	0.3829848	0.9254736	0.9180443
PMPCA	4	2062;4376;42970;46380	TGAATCATCTCGGTGAGAAG;CTTACG	2	1.7386276	1.3660003	0.3831324	0.9237165	0.9180443
PGLYRP2	4	2890;4380;50036;66058	GGTATCTCCAGTCTCCCAAG;AATCCT	2	1.7378835	1.2997934	0.3836854	0.9229426	0.9180443
EEPD1	4	1040;4383;14405;29958	GGAGAAGGCCAACAACCCCG;GAGAT	2	1.7373258	1.5048737	0.384148	0.9219552	0.9180443
EEF1A2	4	3674;4385;9079;32332	GGTGGTCTCGAACTTCCAGA;TGTTCT	2	1.7369543	1.2543608	0.3844183	0.9205118	0.9180443
BHLHE23	4	933;4393;7802;11832	ACTAAGCCACGGCTACGCGG;GCGTA	2	1.7354699	1.5265758	0.3855825	0.9212107	0.9180443
IGFLR1	4	2314;4396;21127;36714	GCAGTGCAACCCCGACGGCG;TATGA	2	1.734914	1.3413705	0.3860014	0.9201298	0.9180443
RPS6KA6	4	610;4400;20195;35728	AAGCAAGGGCCAGTTCTGCG;CCAGA	2	1.7341734	1.6153568	0.3865971	0.9194741	0.9180443
FAM46C	4	3565;4402;17028;34861	GGATAGGTACAACTTCAGTG;CCTCTG	2	1.7338034	1.258326	0.3868653	0.9180443	0.9180443
CD40	4	3976;4431;13623;15965	AGGAATTCGCTTTCACCGCA;GAAAC	2	1.7284585	1.2357356	0.3912447	0.9263551	0.9226051
MAN2A2	4	1281;4447;11702;52085	AGAAGGAGACCTCTGCCCAG;CATCT	2	1.7255255	1.4558509	0.3934807	0.929565	0.9226051
PMEPA1	4	2664;4464;16829;38530	CCTGACACTGTGCTCTCCGA;TCTGA	2	1.7224217	1.307697	0.3958798	0.9331453	0.9226051
UST	4	1430;4474;35653;36223	AAGAAGCAGCAGCATCCCGG;AAGCC	2	1.7206018	1.4308192	0.397543	0.9349787	0.9226051
DMRTC2	4	941;4489;33844;51770	GCCAGTGTCCAGGGACCCAA;CTTGT	2	1.7178801	1.5159976	0.3997665	0.9381188	0.9226051
CYBA	4	1364;4495;8665;49839	GGCCCACTCGATCTGCCCCA;ACAGA	2	1.7167942	1.4385896	0.4006387	0.9380808	0.9226051
REM1	4	1783;4497;19020;36901	GACCTCCATGAACAGCTGGG;GATCT	2	1.7164326	1.3839736	0.4009328	0.9366927	0.9226051

OR51D1	4	2188;4499;44211;52292	CCAAAAGGAAGTATGCTGCG;GGCCA	2	1.7160711	1.3429739	0.4012187	0.9352913	0.9226051
C9orf142	4	2574;4505;12728;59340	CTTGGGTCTCACCTGAACCG;GCAGG	2	1.7149877	1.3106293	0.4020909	0.9352598	0.9226051
KIF3B	4	3863;4508;10539;37366	GGACAGAGTCAACAAGTGGT;TGTCT	2	1.7144466	1.2339503	0.402543	0.9342537	0.9226051
USP51	4	906;4509;21935;55410	TCCACATGAGAGCAGCCTGT;GACGG	2	1.7142664	1.5221123	0.4026958	0.9325588	0.9226051
TCF3	4	3874;4517;17877;48015	TCACCATGCTGAAGTCCAGG;TGAAC	2	1.7128257	1.2326236	0.4038424	0.9331676	0.9226051
OSER1	4	3880;4521;13456;49514	GCATCTAAAGACAGTTGGCA;TCCTG	2	1.7121064	1.2319831	0.4044183	0.9324579	0.9226051
WDR81	4	3137;4528;23171;25975	CTTCTGAGTCAGCGTCACCG;GATTG	2	1.7108492	1.2707129	0.4055649	0.9330643	0.9226051
DNAJC6	4	1584;4530;20642;37519	CTTTGCATATAGCAATCGAA;GTAGGT	2	1.7104904	1.4049248	0.4058258	0.9316348	0.9226051
FGF9	4	2513;4533;14986;68310	TAGTTCCCAACTTCACCTAA;AGTCCT	2	1.7099525	1.3127689	0.4062852	0.9306664	0.9226051
TMEM40	4	749;4541;44497;48398	GGAGGATGCTGAAGTCTCCA;CACTC	2	1.70852	1.5591776	0.4074006	0.9312014	0.9226051
ANKRD16	4	3965;4554;27324;49163	TCCGCTCCACACCTACCAGT;GCAGG	2	1.7061979	1.2251056	0.4092593	0.9334293	0.9226051
STARD9	4	3262;4558;13524;16593	CTACTGGTCAGTCAACCCAG;GCAGC	2	1.7054849	1.2606851	0.409831	0.9327188	0.9226051
DUSP7	4	2288;4562;54463;59955	CGAGTCGTCGCACATCGAGA;GCACA	2	1.7047725	1.3285194	0.4104006	0.9320066	0.9226051
ASXL1	4	3977;4581;14294;29775	GACTCCTCTGAAGGTAAACG;TACCT	2	1.7013978	1.2221599	0.4133788	0.9367554	0.9226051
ASB7	4	4306;4585;18456;35424	TGTGGCTGCCCACTACGGCA;CCTCA	2	1.7006893	1.2075845	0.4139464	0.9360329	0.9226051
KIF1C	4	3106;4588;15359;47427	TGATGGGGCGACAGGAGCCA;TGGA	2	1.7001583	1.2672418	0.4144256	0.9351141	0.9226051
SRXN1	4	3702;4593;35364;72618	CAGCGGCACGTTGTGCACCG;TCACC	2	1.6992742	1.2341291	0.4151709	0.9347985	0.9226051
UTY	4	4361;4595;49385;72748	TGATGAGGCAAAGAAAATGG;GTATCT	2	1.6989209	1.2044536	0.4154859	0.9335172	0.9226051
GPC3	4	1876;4602;41094;52298	GGTCACGTCTTGCTCCTCGG;ACATT	2	1.6976854	1.3643908	0.4165534	0.9339287	0.9226051
PPA1	4	2686;4604;62408;73990	GTACTCCAGGGAGAAGGGCG;GAGG	2	1.6973328	1.2935656	0.4168331	0.9325757	0.9226051
POLR3H	4	2575;4609;39867;61562	GCTGCTGCAGTGACTCTGGG;TGTGT	2	1.696452	1.3012861	0.4175909	0.9322959	0.9226051
GLP2R	4	2532;4619;15994;70373	ACTTTCAGAGAGCTCAGGAA;CTACT	2	1.6946934	1.3036753	0.4190742	0.9336338	0.9226051
PKP4	4	4344;4621;43942;74505	TTACTCCTATATATGAGGGG;ACATCT	2	1.6943422	1.2028549	0.4193383	0.9322552	0.9226051
SETD2	4	4377;4622;47862;52553	CGGAGCTGATACTTACTCAG;GTAGAT	2	1.6941667	1.2014295	0.419489	0.9306311	0.9226051
FAIM	4	4111;4632;31223;51466	AGGAAGAGATAAGAAAAGAG;ATAAAT	2	1.6924134	1.2117075	0.4210763	0.9321941	0.9226051
KRTAP9-2	4	1489;4633;31976;66249	GCAGGTGGTCCTGCAGCAGG;GCAG	2	1.6922383	1.408383	0.421225	0.9305723	0.9226051
IRF2BP1	4	2662;4645;20678;45465	CATGGTGTGGGACTTCAGCG;CTTGA	2	1.6901403	1.2917013	0.4230119	0.932569	0.9226051
MPHOSPH	4	1176;4656;12460;61540	GAGCACATAGATGTTCCCAA;GTATCT	2	1.6882222	1.454839	0.424674	0.9342829	0.9226051
THAP3	4	3443;4659;55462;66007	GCTTGGGCTTGAAGTTGCCC;TAGGT	2	1.6876999	1.2417191	0.425147	0.9333789	0.9226051
KRTAP21-1	4	597;4665;8269;59363	TGTGGCTATGGAACTGGCTA;AACTC	2	1.6866565	1.5961617	0.4260701	0.9334648	0.9226051
GNRH1	4	3265;4667;29113;63034	CTACTGACTTGGTGCGTGGA;CGGCA	2	1.686309	1.2509249	0.4263674	0.9321821	0.9226051
CCDC114	4	1689;4670;9388;21279	GGAGCTGCAGGAGCAGACCA;AGCG	2	1.6857881	1.3795728	0.4268331	0.9312722	0.9226051
POLR2A	4	118;4673;28261;35661	GTACAATGCAGACTTTGACG;TTCGGT	2	1.6852675	1.9433153	0.4272884	0.9303434	0.9226051
KRTAP10-9	4	3932;4681;13302;58823	GCTGGAGCGGATGGACATGG;AGGA	2	1.683881	1.2154581	0.4284932	0.9310469	0.9226051
SKOR1	4	3415;4684;12224;25086	GGTAGCCCGAGGGAAAAACG;TGATG	2	1.6833617	1.2410671	0.4289724	0.9301742	0.9226051
SEPW1	4	4007;4688;49802;50824	CAAGACTCACGATGTCCAGG;TTACC	2	1.6826699	1.2114405	0.4297115	0.9298674	0.9226051
TMBIM4	4	3029;4691;35671;52645	GTACCCTCGCTCCTCGATCG;CGTCC	2	1.6821515	1.2629891	0.430173	0.9289626	0.9226051
TECPR2	4	2091;4704;42775;47710	GCAAGCGGAGAAGCTGCCAG;GCTAT	2	1.6799091	1.3338685	0.4321034	0.9312268	0.9226051
MYO1C	4	663;4712;47786;61705	GATGATCTCTGGGGAGAGCG;GACAG	2	1.6785324	1.5699107	0.4332988	0.9319013	0.9226051
LETM1	4	2241;4713;27543;46081	CCTCGGTACACCGTCCCGCG;TCAGC	2	1.6783605	1.3193978	0.4334578	0.9303485	0.9226051
MKNK2	4	2140;4723;25913;34459	GGCCAAAGTCAGAGTCTCCG;AGGG	2	1.6766437	1.3276458	0.4349651	0.93169	0.9226051
GSG1L	4	3517;4726;42607;55710	TGTGGGCGACCATTCCCAGG;CTTGG	2	1.6761294	1.2319931	0.4353996	0.9307328	0.9226051
PAPL	4	3548;4728;30382;67952	GCAGCTTGCGAAGCGTGACT;TATTCT	2	1.6757867	1.2301999	0.4357084	0.9295112	0.9226051
IKBKAP	4	165;4730;17745;45241	CCTGACAGAGAACATGCAGC;AATGG	2	1.6754442	1.8660134	0.4360088	0.9282767	0.9226051
TAS2R30	4	1775;4733;24187;27290	TCTGGGTGTTATTACTACAT;TGAGCA	2	1.6749307	1.3641277	0.4364516	0.9273499	0.9226051
OR2T11	4	101;4747;22068;39542	GACAGTGGTACAGATGAAAA;TCAGG	2	1.6725391	1.9705829	0.4386855	0.9302246	0.9226051
MYH7B	4	1026;4748;10439;21069	CTCACAGCTGTCAATATCCG;GATCAC	2	1.6723686	1.4752142	0.4388601	0.92873	0.9226051
TIGIT	4	3487;4749;12556;57340	GCTGACCGTGAACGATACAG;GTTCA	2	1.6721981	1.2316131	0.4390192	0.9272085	0.9226051
MAFB	4	1583;4756;18937;53957	GAGCTACACGGAGTGCTGAG;AAGCT	2	1.6710056	1.3853107	0.4401096	0.9276562	0.9226051
SLC32A1	4	708;4777;23919;42976	GATGGACATCCTGAAAGCCG;TCATG	2	1.6674397	1.5505006	0.4432988	0.932517	0.9226051
TRHDE	4	3110;4782;11813;63858	TTGCGGGTAGAGGAAAAAC;CAGCT	2	1.6665932	1.2502162	0.44412	0.9323871	0.9226051
AMPH	4	816;4791;10210;53867	AAGCTCTGCAGAGCTCCAAG;AGACT	2	1.6650718	1.5194399	0.4456418	0.9337258	0.9226051
CREM	4	936;4794;37889;57441	GTAAGTTGGCATGTCACCAG;CCTACT	2	1.6645654	1.4904529	0.4461034	0.9328419	0.9226051
CNN3	4	3144;4803;21356;44993	GGGCTGTAACTCACTCGCAG;GACTT	2	1.6630481	1.2463921	0.4474994	0.9339118	0.9226051
CAST	4	1576;4804;11257;19754	CGACTTCCTTAATTGAGCGG;TGTCTT	2	1.6628797	1.3821477	0.4476574	0.9323989	0.9226051
TNMD	4	1600;4809;14161;44694	GGACAATTAGAGTTAGGGCC;GAGCA	2	1.6620383	1.378659	0.4483716	0.932048	0.9226051
FHL2	4	4158;4814;31199;46530	GGAGGAGAGCCCCTACTGCG;GAGG	2	1.6611979	1.1940651	0.4491533	0.9318386	0.9226051
MICALL1	4	3713;4816;37116;63989	TCAGGGATCGACATACCCCT;GGTCG	2	1.6608619	1.2143796	0.4494745	0.9306766	0.9226051
H3F3C	4	2141;4817;48582;67349	GCTTCACCCCGCAGGTAGAG;CAGAC	2	1.660694	1.3195785	0.4496138	0.9291431	0.9226051
TRABD2A	4	4384;4818;24878;70973	AGCTGTCAGATGCTGCCACA;TGGAT	2	1.6605262	1.1843271	0.4497645	0.9276393	0.9226051
ERGIC3	4	607;4826;11237;16773	GGTGCATCCTGAGCTCTACG;GGTGA	2	1.6591846	1.5789065	0.4510254	0.9284266	0.9226051
F9	4	2468;4833;16268;51627	TTTCTAGTGCCATTTCCATG;GTAACAT	2	1.6580127	1.2903166	0.4521502	0.9289311	0.9226051
PKIG	4	1163;4845;11284;31582	CCTGTGACCGGACAGGCCGT;TGTCA	2	1.6560079	1.4410304	0.4541491	0.9312262	0.9226051
PTEN	4	756;4856;7878;17738	GAGTAACTATTCCCAGTCAG;AGAGG	2	1.6541749	1.5300467	0.4558851	0.9329741	0.9226051
C14orf79	4	2696;4857;21901;53986	AAGTGCTGCTGTGTTCCCCA;GGAGA	2	1.6540085	1.271187	0.4560223	0.9314498	0.9226051
COLEC12	4	1405;4862;24418;61537	GCAGAACCTGAAAGACCTGC;TCTCA	2	1.653177	1.4007144	0.4568092	0.9312558	0.9226051
G2E3	4	3837;4871;42754;60508	ATTCTCTCTGAAGTCCACAT;GTAGGT	2	1.6516826	1.2037957	0.4582094	0.9323105	0.9226051
FAM127C	6	871;3139;9832;23867;261	GTTCTCGTCCACGAACATGT;GTTCCT	2	1.6514062	1.4159772	0.458515	0.9311382	0.9226051
GALP	4	2052;4883;42789;63992	GGACGAGGAGGAGGACCAGG;GGCT	2	1.6496947	1.3224998	0.4601263	0.9326168	0.9226051
DDAH2	4	33;4886;48090;64200	GCAAAGCCCTGCAAGACCTG;GCTCA	2	1.6491986	2.2012227	0.4605805	0.9317491	0.9226051
LRRC14	4	3104;4889;56657;67100	ATAAGTCCATACCTGCAGAG;GGTCC	2	1.6487028	1.2416357	0.4610712	0.9309582	0.9226051
STIL	4	3223;4890;38989;39729	TTTCAGCGCTCAGTTCACAA;CAGGT	2	1.6485376	1.2344677	0.4612396	0.929521	0.9226051
FAM160A2	4	747;4891;24914;29462	GGAACTCATGAAGAGTGCAA;GGATC	2	1.6483724	1.5296668	0.4613841	0.9280411	0.9226051
CYSLTR2	4	2503;4892;32893;57496	GTATCAGAAATGGAACCAAA;GGATCC	2	1.6482073	1.2826718	0.4615483	0.9266065	0.9226051
CFAP46	4	3445;4913;36533;62851	ACTTTGCCAAAGGAGAACCG;GATGA	2	1.644748	1.2201353	0.4650244	0.9318136	0.9226051
SH2D7	4	4250;4914;14112;60913	GTAGGACAAGATGTAGCCAG;GCTTG	2	1.6445837	1.1818562	0.4651866	0.9303731	0.9226051

PVALB	4	1708;4925;32795;69442	GAGGACATCAAGAAGGCGGT;CGGCC	2	1.6427785	1.3558085	0.4669568	0.9321482	0.9226051
MYO3B	4	2661;4926;30327;64403	TATTCCCCTTCACATCACGG;GCCCCC	2	1.6426146	1.268011	0.46712	0.9307146	0.9226051
LY6E	4	4019;4930;31316;47621	CACGCAGTAGTTGTCCTGGT;ACCTCC	2	1.6419594	1.1905465	0.4677572	0.9302291	0.9226051
VCX2	5	1856;3865;32878;39735;4	GGCCTTGGCCGGAGGTCCCG;GGGA	2	1.6414215	1.2954168	0.4684225	0.9298011	0.9226051
CDH20	4	2903;4941;38864;42611	GCATGTCCTTGTACTTCTGG;TTACCT	2	1.6401605	1.2500753	0.469515	0.9302212	0.9226051
UXT	4	3700;4945;7671;56113	GCTCATATACCTTGTCTCGA;TGGCGA	2	1.6395074	1.2043447	0.4701585	0.9297516	0.9226051
KDM3A	4	3274;4958;14157;33269	GATACTGCTTGGCTGTACTG;AGAACA	2	1.6373889	1.2259492	0.4723997	0.9324375	0.9226051
MRC1	4	1293;4964;14896;62605	AGATGAGAAAAAAATCCAGA;TTAATG	2	1.6364131	1.4093765	0.4733789	0.932627	0.9226051
CHRNA4	4	1016;4965;9330;21573	GGGCAGGTAGAAGACCAGCA;AGAAC	2	1.6362506	1.4591936	0.473539	0.9312051	0.9226051
KLK9	4	3668;4967;26988;43801	GGGCAGCTGTGAGCAGCCAG;GCTCT	2	1.6359257	1.2041464	0.4738664	0.9301169	0.9226051
IL5RA	4	4742;4970;13715;35158	GTATCTCAGTGGCCCCCAAA;TTAATG	2	1.6354387	1.1580636	0.4743695	0.929377	0.9226051
CNTD1	4	1926;4975;23823;28955	GAGAGAGTCTCAGAATTGGA;GCTGA	2	1.6346276	1.3275977	0.4751793	0.9292395	0.9226051
FAM122C	4	4801;4977;42581;65375	ACTGATCAAGAAACACAGCT;GATCAG	2	1.6343034	1.1553606	0.4755088	0.9281651	0.9226051
PSIP1	4	580;4983;13011;13194	TGAGTTCCAAAAAAGAAAAT;TGAAGA	2	1.6333316	1.575623	0.4764662	0.9283179	0.9226051
PHYHIPL	4	4824;4987;22277;52150	GGGAACAAATCACAAGACAG;CCACG	2	1.6326845	1.1537278	0.4771159	0.9278718	0.9226051
PTGER1	4	3869;4988;9548;14530	CATGGAGAAGATGGGCAGCG;GCTGC	2	1.6325228	1.1927066	0.4772812	0.926487	0.9226051
SSH3	4	1129;4992;19096;56144	GCTCTCTCCGCAGGACCAGG;ACACT	2	1.6318764	1.4351064	0.477934	0.9260519	0.9226051
LRRC37A	5	404;3918;8117;39859;724	AGGAGCTGTAGTCTTCTCCA;GGCATC	2	1.630567	1.6039415	0.479355	0.9271041	0.9226051
PXDNL	8	2169;2383;11443;22974;5	GCATGGCCAGAAATTCCGCT;TGTCAT	2	1.6295801	1.176535	0.4803519	0.9273337	0.9226051
CHD1L	4	4132;5007;33548;47069	ATAAACTGCTGGCCTCTGAG;ATACTG	2	1.6294573	1.1793169	0.480514	0.925954	0.9226051
MYSM1	4	3649;5016;36366;42239	AATGGTAGAGGAAAGCCATG;AAAAAC	2	1.6280097	1.2011418	0.4819246	0.9269807	0.9226051
SOD1	4	147;5017;44323;66660	AAGGCCGTGTGCGTGCTGAA;AATCC	2	1.627849	1.8671418	0.4820805	0.9255946	0.9226051
SLC13A3	4	3506;5021;13003;13691	TTAAAGCAAAGACCACCCTG;GATGCT	2	1.6272066	1.2081113	0.4827541	0.9252058	0.9226051
SGK2	4	3652;5029;14524;52499	GTGTGCTTCTGAAGAACGTG;CCGTC	2	1.6259235	1.1999478	0.4840774	0.9260612	0.9226051
GRM2	4	734;5031;20927;21878	CATCGCGTCACAGAGCCGGG;CCACA	2	1.6256031	1.5219799	0.4844132	0.9250277	0.9226051
GPRC5A	4	4085;5033;67155;72256	AGAAACGGTGGCCACAGCCG;GCCTT	2	1.6252828	1.1792799	0.4847334	0.9239683	0.9226051
RCSD1	4	505;5034;14918;67294	CAGCCAGTAAACCAACCCGA;TGTGC	2	1.6251227	1.6009279	0.4849069	0.9226338	0.9226051
HMGN1	4	640;5039;64328;72915	AGCCCAAGAGGAGATCGGCG;TTTCT	2	1.6243228	1.5502702	0.4857656	0.9226051	0.9226051
PIK3R5	4	2471;5050;23739;44700	GAGCCTCACCTAACACCCCA;GATCCT	2	1.6225659	1.2723565	0.4875733	0.9243759	0.922712
PPP2R3A	4	737;5058;33910;37449	CTGTCTCGATACAATGACCA;ACATAC	2	1.6212908	1.5189644	0.4890067	0.9254321	0.922712
PCSK9	4	3421;5060;13262;40510	GATGCTCTGGGCAAAGACAG;TCTTTC	2	1.6209724	1.2095461	0.4893394	0.924405	0.922712
PLEKHA3	4	2434;5063;33100;72367	TCATTCATCTCCTAGTGCAG;GCATTA	2	1.620495	1.2742634	0.4898435	0.923705	0.922712
TSGA10	4	4454;5065;30411;67859	TGAGCTGGCCCAGATCGCCA;CTAAG	2	1.6201769	1.1613616	0.4901907	0.922712	0.922712
OR4P4	4	1641;5084;18631;61339	TAGAGATCTTCATTCTCACA;GCACTT	2	1.6171618	1.351092	0.4932812	0.9268771	0.9244853
ERMN	4	4254;5086;16806;64211	TCCTGGTTACTGCCACTCAG;TGTTCC	2	1.6168451	1.1678196	0.4936149	0.9258566	0.9244853
KIFC3	4	285;5087;11610;69004	AGGTGCAGCAGAAGACCGAC;GCAGT	2	1.6166869	1.7190041	0.4937781	0.9245206	0.9244853
MYL9	4	1574;5095;8570;33846	AAGCCAAGACCACCAAGAAG;GTCAA	2	1.6154218	1.3586767	0.4951336	0.9254178	0.9244853
C12orf60	4	4768;5102;12242;13292	AAAGGAGTCTTTATGTTCCA;GGATTT	2	1.6143166	1.1465574	0.4962334	0.9258347	0.9244853
ZNF562	4	3794;5107;13864;26184	ACTCTGTGAGAATTGTGGAG;TCAGC	2	1.6135282	1.1867705	0.4972282	0.9260546	0.9244853
SLPI	4	3878;5108;10688;52434	AGAGTGACTGGCAGTGTCCA;GCCTT	2	1.6133707	1.1827088	0.4973976	0.9247392	0.9244853
SIMC1	4	2023;5117;46038;68812	ATCGTGATCTCGGACGACAG;GATCAC	2	1.611954	1.3064601	0.4988508	0.925811	0.9244853
EFNA2	4	1361;5118;22911;49402	GGTTCCAGTAGACGGCGTAG;GCTGT	2	1.6117967	1.3865423	0.499012	0.9244853	0.9244853
ATP5J2	4	96;5127;14478;38870	AGAAGTTTCTTGTCCTTCAC;ACTTCT	2	1.610383	1.9504863	0.5005234	0.9256615	0.9256615
MS4A12	4	2857;5135;46190;60363	GGAGCCATAAAGCTGCTTGG;TCTAAC	2	1.6091287	1.2376104	0.5018373	0.9264689	0.9264689
BARHL2	4	1939;5141;54480;56936	AGAAGACGTGGAAGTCCTGG;GGCCT	2	1.6081893	1.3130342	0.5028467	0.9267122	0.9267122
SDK2	4	2462;5150;63055;63661	CCATGCATGTAAGCACCAGG;ACAGCA	2	1.6067825	1.2651759	0.5043519	0.9278669	0.9268927
RNF128	4	1526;5163;26478;43129	CTTTCTATCAGCGCAATCCA;TTGAATA	2	1.6047551	1.3596408	0.5066637	0.930499	0.9268927
ZNF783	4	1786;5167;19155;39299	GAACTCGTACCTCTACTCCA;CTCCAG	2	1.6041324	1.3274853	0.5072916	0.9300346	0.9268927
HS3ST2	4	5049;5170;40174;62205	CTATAGGGTCCTGGGCCGCG;CACCC	2	1.6036657	1.1314208	0.5077781	0.9293131	0.9268927
ZNF491	4	3936;5181;64656;73917	GTGTGGAGAAACTTTCACCC;ACTGCT	2	1.601957	1.1743121	0.5096305	0.9310896	0.9268927
IGFBP7	4	995;5182;19514;60017	GAGGCGGAAGGGTAAAGCCG;GCTGC	2	1.6018019	1.4463189	0.5097937	0.9297791	0.9268927
C15orf62	4	2865;5183;29409;44564	GCTCAGCTGCTTGAAGAAGG;GCTTG	2	1.6016468	1.2333349	0.5099506	0.9284618	0.9268927
MAP3K12	4	449;5194;47954;49604	CCTGCACAAGATTATCCACA;TCACCA	2	1.5999428	1.6133689	0.5118113	0.9302457	0.9268927
RASGEF1C	4	1665;5208;19248;57749	GCCGGCTTACCAGTTCCACG;CACAG	2	1.5977797	1.3384622	0.5142178	0.9330137	0.9268927
EFNA3	4	633;5214;42160;66162	CCCGCACTACAACAGCTCGG;GTCGT	2	1.5968547	1.5388627	0.5153831	0.9335241	0.9268927
DDX27	4	909;5215;15425;32719	ATACACTCAAAGTAAAGGAT;TGATGAC	2	1.5967006	1.4626381	0.5155494	0.9322263	0.9268927
DOK5	4	4391;5216;21505;26502	TTAATGACATAGTGAAGCAA;GAGGAT	2	1.5965465	1.1520557	0.5157095	0.9309217	0.9268927
CEBPB	4	582;5221;14719;34010	ATGGATTTAAAGGCAGGCGG;CCTCTT	2	1.5957768	1.5561157	0.5165307	0.930813	0.9268927
LYPD5	4	172;5230;18067;25855	CGCACCACCGAGTAGTCTGG;GCTGC	2	1.5943934	1.8165269	0.5180369	0.9319369	0.9268927
PPM1B	4	61;5232;22073;22754	ACTTTCCTCTGCTTCATCGG;CCAGCA	2	1.5940863	2.040503	0.5183602	0.9309326	0.9268927
PRDM1	4	596;5241;36877;43084	GGATGGGGTAAACGACCCGA;CTTGA	2	1.592706	1.5495417	0.5199402	0.9321849	0.9268927
RBFOX3	4	3505;5243;39172;71065	GGACTACTCCGGCCAGACCC;GCAGC	2	1.5923996	1.1907606	0.5202822	0.931217	0.9268927
SERPINE1	4	3523;5254;13248;47152	CTCCTTGTACAGATGCCGGA;GGACC	2	1.5907168	1.1889716	0.5221398	0.9329605	0.9268927
TAS2R38	4	1168;5259;34028;74250	GCTTGCCAAGCAGATCAGGA;AGGAG	2	1.5899532	1.4071158	0.5230307	0.9329737	0.9268927
CST9L	4	5140;5270;16348;62828	GTGGACAGCAAACTCCACTG;ACTACT	2	1.5882759	1.1206988	0.5250078	0.9349212	0.9268927
TAC3	4	2375;5278;43115;54877	AGCTAGGCTGAAGGCCAGGA;GTAGA	2	1.5870585	1.2623419	0.5263529	0.9357386	0.9268927
RSPH6A	4	942;5285;39108;55640	GAGACTCCAGGACAGACAAG;GGACT	2	1.5859949	1.4498331	0.5275213	0.9362396	0.9268927
ZNF253	4	4869;5288;35368;70993	TTTCACTCGTACCTACCTGG;TGCACA	2	1.5855395	1.1285588	0.5280297	0.9355693	0.9268927
KAT2A	4	3503;5289;32198;41157	GCGCTGCCTGCCTTACCCAA;GCTGC	2	1.5853878	1.1873603	0.5281929	0.9342909	0.9268927
FBXO28	4	691;5299;12629;50591	ATGTTCTCGATGGCTACGAT;CGAAAT	2	1.5838722	1.5138448	0.5298769	0.9357023	0.9268927
KIR2DL3	4	1977;5302;46782;54348	TGTCTCTTCTGATTTCACCA;ACTTCCT	2	1.5834182	1.2967746	0.5303581	0.9349886	0.9268927
PITX3	4	4672;5306;10967;13914	GTTCGCTGAAAAAGAAGCAG;AGTTG	2	1.5828132	1.1343286	0.5310494	0.934647	0.9268927
MZT2A	4	2519;5310;16241;71878	GGACCTGCTGAAGCTGAACG;GCTGT	2	1.5822087	1.2484333	0.5317085	0.9342498	0.9268927
KIAA0408	4	4455;5318;63546;68146	GTTGTCTCAAAGGGAAGTGA;CTATGT	2	1.5810013	1.1417343	0.5332282	0.9353638	0.9268927
TP53TG5	4	3927;5325;15926;44784	GAAGAGGCCCAAGAACAGCA;TATGC	2	1.5799464	1.163721	0.5343987	0.9358624	0.9268927
RGL2	4	2822;5326;16690;23068	TCTCTCTCACCTCTGCCACG;CCTCAC	2	1.5797958	1.2253033	0.5345785	0.9346273	0.9268927

TRIM49D1	4	672;5328;32020;46785	AAATCAGAGAAACCTGAACG;GCTCC	2	1.5794947	1.5175431	0.5349538	0.9337375	0.9268927
GIP	4	1509;5329;33163;62920	GGACTAGGAGAGAAGAAAGA;ACCTT	2	1.5793442	1.3492167	0.5351139	0.9324756	0.9268927
ZNF354A	4	2258;5334;21868;40007	GAGAAAAAGCAGGATAAAAA;TGCTG	2	1.5785923	1.2680255	0.5360047	0.9324893	0.9268927
RPS23	4	2095;5335;25764;57787	GACCCTTACACACTTCCTAA;TCTGGT	2	1.578442	1.2827559	0.5361773	0.9312553	0.9268927
PNMA6A	5	2873;4184;22361;28843;	GCTAGGCAAAGTGTTTCGAG;AGGCA	2	1.5784189	1.181251	0.5361773	0.9297261	0.9268927
NPY1R	4	4880;5338;16501;30306	AGATGATTATGATCAAGGCC;ATGTGT	2	1.5779913	1.1243973	0.5366721	0.9290585	0.9268927
MDN1	4	5125;5339;30055;30794	AGAACCTGCCCAACTCACTG;TATTCC	2	1.5778411	1.1159756	0.5368529	0.9278506	0.9268927
FOXF2	4	1208;5348;30795;45722	TCCAGCCCTGGTAGGCGCCG;GGAC	2	1.5764909	1.3934271	0.5384299	0.9290555	0.9268927
ZNF772	4	1781;5349;28841;29899	GCACAGTTGTTCAGAAGAAA;CTGCT	2	1.576341	1.3141537	0.5385983	0.92783	0.9268927
SDC4	4	4591;5351;10736;27141	TCAGGGATATGGTTATCTAG;GTAGGG	2	1.5760413	1.1339872	0.538932	0.9268927	0.9268927
FBXO24	4	1243;5367;9076;30473	TCTTGCTGAGACATCGGCGG;GTAGT	2	1.5736485	1.3861142	0.5417615	0.9302441	0.9302441
DAB2	4	4097;5393;19568;22507	GTATCTGAAAGAGAACAGAA;GCTGT	2	1.5697767	1.1510006	0.5463665	0.9366282	0.93057
SSMEM1	4	537;5400;33633;36787	GCAAAGAGACTTCCTGTAAG;CCAAA	2	1.5687378	1.559675	0.5476139	0.9372451	0.93057
TMEM31	4	3832;5407;20208;31726	ACCTTCTTGAAGTCCTTCCG;AGGTTA	2	1.5677003	1.1620417	0.5488478	0.937837	0.93057
TMEM37	4	2079;5409;40763;59664	GCAGATCGTCTGGTTGGTGG;GATTC	2	1.5674042	1.2787582	0.5491939	0.9369124	0.93057
ANGPTL1	4	5273;5410;23638;51666	GATGGACCTTGAAAACCTGA;GGGAG	2	1.5672561	1.1058886	0.5493686	0.9356987	0.93057
SCGB2A2	4	4295;5411;33300;66853	AAATCACATTCTCCAATAAG;CCTCAC	2	1.5671081	1.1412476	0.5495546	0.9345083	0.93057
IL20	4	2089;5413;12726;37295	TTGACATCAGAATCTTAAGG;GATAAA	2	1.5668122	1.2775099	0.5499465	0.9336713	0.93057
ARPC1B	4	1425;5419;13092;23950	CTCCTGCTCGAAATAACAGA;CATCTG	2	1.5659252	1.3541971	0.5509725	0.9339117	0.93057
AKAP17A	4	1840;5438;52739;60637	GCTCTTGGAAATACGCAGCG;GCGTAT	2	1.5631234	1.3009953	0.5544122	0.9382361	0.93057
ZCCHC18	4	3704;5439;15294;42443	GCAAGAAATCTGCTACACTT;TTAACA	2	1.5629762	1.1658811	0.5545817	0.9370212	0.93057
DUS4L	4	249;5449;12297;17078	AATGGAATAGACATTAACTG;AATAGA	2	1.5615062	1.7204214	0.5563187	0.9384545	0.93057
MAP4K4	4	491;5453;14910;55024	GGAGAACAAGGAACGTTCCG;GACCT	2	1.560919	1.574808	0.5570318	0.9381588	0.93057
CD1A	4	4296;5462;40944;41419	TTTGAGATACAGGTGACAGG;CCTGG	2	1.5595995	1.1374519	0.5585869	0.9392798	0.93057
GATSL1	6	986;3528;52869;54871;56	ATACGTGGACAGCATGAACA;AGTATA	2	1.5594023	1.3447387	0.5589361	0.9383729	0.93057
ZNF286A	4	3179;5466;44990;51157	AATTGGTTAGAGAATCAGCA;GGCCAT	2	1.5590138	1.1922897	0.5593156	0.9375194	0.93057
FUT10	4	375;5475;13711;44404	CGAGCTGATGACTTACATCG;TCTGAC	2	1.5576978	1.6307049	0.561063	0.938958	0.93057
PTPN12	4	3871;5477;8376;61232	TTTGTGCCATAGATTATACG;GTAAATC	2	1.5574056	1.1550542	0.5614216	0.9380715	0.93057
CASP5	4	4476;5484;10639;73208	GGCATCCTAGAGGGAATCTG;CATGT	2	1.5563841	1.1285993	0.5627095	0.9387382	0.93057
BTBD9	4	2038;5485;42310;67700	TTAAAGTGCGATCTGAGAGC;GCTGA	2	1.5562383	1.2771326	0.5628779	0.937538	0.93057
CPNE1	4	5321;5491;7473;17966	TTTCTGGAGTTCTTCCGCCA;GGAAAT	2	1.5553639	1.0984254	0.5639632	0.9378663	0.93057
GDAP1L1	4	1649;5492;16970;29505	AGGTTGAGCCGCATGAACCA;GGCCG	2	1.5552183	1.3191356	0.5641357	0.9366782	0.93057
CYP26B1	4	158;5505;26312;63664	TTACCTGCCCAAGATCCAGC;ACTGCT	2	1.5533279	1.8143075	0.5664694	0.9390764	0.93057
ARIH2	4	3323;5513;23911;44129	GGACATAGAGGACTATTACG;ATATCT	2	1.552167	1.1805596	0.5679029	0.9399772	0.93057
CCL19	4	1470;5518;9625;51850	AGTTCCTCACGATGTACCCA;CATCCT	2	1.5514424	1.3406047	0.5687958	0.9399818	0.93057
RBMXL1	4	5271;5526;39698;60431	AGAAGTAGAGGTCCTCCAAG;GGAGG	2	1.5502845	1.0974664	0.5702854	0.9409709	0.93057
ACRV1	4	5414;5530;7490;45829	CCTGAAGGCTGTTCTCCTGA;CCTTC	2	1.5497063	1.092708	0.5711648	0.9409517	0.93057
CBLN3	4	719;5533;15701;26835	GCAAATGCCACTCGCCCAGG;GCCAG	2	1.5492729	1.4881662	0.5717179	0.9403957	0.93057
EID2	4	2327;5534;23651;36782	GGCCTCCTCAGGCTGTGCGG;GGCA	2	1.5491284	1.2473782	0.5718831	0.9392046	0.93057
SMIM2	4	1667;5536;29804;33923	GATCCAAGCGAGGACTATGT;CGTGTT	2	1.5488397	1.3137494	0.5722324	0.938319	0.93057
EVI2B	4	1642;5547;49301;53831	CTAATTCTCAAAACACAACA;TGTTTG	2	1.5472537	1.3160146	0.5742231	0.9401233	0.93057
CARM1	4	1672;5553;63707;68749	GGTGAAGAGTAACAACCTGA;ACCCA	2	1.5463901	1.3119188	0.5752677	0.9403758	0.93057
BTNL9	4	2400;5561;12138;52653	AGCATCATCCCCTCTGACAA;GCTCAA	2	1.5452402	1.2393842	0.5767085	0.9412738	0.93057
UGT1A10	4	1106;5562;12434;45843	CTATTAATGAGTTCATCCAG;GTTCTCT	2	1.5450966	1.3959822	0.5768603	0.9400686	0.93057
DUS2	4	5014;5564;27150;71823	AATGTCCGCTCCATAATCCA;CCATCT	2	1.5448094	1.1031758	0.5772054	0.9391816	0.93057
ITGA8	4	1635;5568;66773;71577	AGCGGCGCCTGGCTTCCCCG;GTTAA	2	1.5442355	1.3153707	0.5778727	0.9388209	0.93057
LOC391003	4	1212;5569;33074;35276	CATGAGTAAGAGACAGACAG;TTGCCT	2	1.544092	1.3765454	0.5780453	0.9376587	0.93057
RIPK3	4	1617;5571;41446;43263	GTTCCTCGATGGACACCAAG;GCAGC	2	1.5438053	1.317399	0.578407	0.9368065	0.93057
PHC3	4	5161;5574;31964;68970	GGAGAGCTGATGACACAAGA;AGACC	2	1.5433754	1.0975599	0.578958	0.9362628	0.93057
PABPC1	4	880;5582;23239;30942	TGGTCGAGCTCAGAAAAAGG;CATAC	2	1.5422302	1.4421875	0.5805962	0.9374765	0.93057
ZNF559-ZN	4	1340;5590;43916;63756	CAGGAGGAAGAATGGCTGCA;TTTCT	2	1.5410868	1.3543775	0.5820152	0.9383328	0.93057
MORC1	4	2840;5594;16986;56229	GTATTGTTTCCAGAGATGCA;GCTGGC	2	1.5405158	1.2044458	0.5827449	0.9380771	0.93057
SOWAHC	4	2435;5595;48300;55766	GCCGCTGCTCTCCTCCTCCG;CCAGC	2	1.5403731	1.2341223	0.5829434	0.9369684	0.93057
TRAP1	4	4155;5604;38586;52439	CCTCATCCAGACCAAGGCCA;CTACA	2	1.5390902	1.1331403	0.5845484	0.9381203	0.93057
PPARA	4	1286;5605;14332;28760	CATCGAATGTAGAATCTGCG;TCTGGC	2	1.5389478	1.3617605	0.5847168	0.9369666	0.93057
RCAN2	4	4448;5609;25762;73506	CGAGGTCTTTACCAATCAGG;GTTCAC	2	1.5383784	1.1206994	0.5854746	0.9367594	0.93057
RNF114	4	5152;5614;11696;27271	AAGCCGGTACAGGTGCCCTG;ATTTG	2	1.5376674	1.0950006	0.5863998	0.9368202	0.93057
GFI1	4	4179;5617;19913;32524	GTACTCATGGAGCGGCCTGG;GCGCC	2	1.5372411	1.1311868	0.5869445	0.936274	0.93057
ZNF181	4	4825;5618;10382;12587	GCTTATCAGATGTCGTGTAA;GAGAAG	2	1.537099	1.1058994	0.5871222	0.9351449	0.93057
SHISA2	4	45;5620;21637;52177	GCGGACAAAGACGGCCCCGA;GCCA	2	1.536815	2.0777864	0.5874913	0.9343234	0.93057
ABCD3	4	5104;5621;22395;45542	TGCTCTGCCTGCTCCACAAG;GGCCT	2	1.536673	1.0960865	0.58767	0.9332024	0.93057
ITCH	4	2299;5622;17425;22608	GAGCCTCCAGATATAGTAAG;ATTTCA	2	1.5365311	1.2434562	0.5878405	0.9320715	0.93057
SNRNP25	4	373;5623;42558;57097	CATTGCCTGGCCGTATTCTA;GTCCGA	2	1.5363892	1.6211943	0.5880339	0.9309802	0.93057
MKS1	4	1651;5629;9496;56212	CCTCTTCGTCTTCCTCTGGG;CTGAA	2	1.5355383	1.3090502	0.5891409	0.9313366	0.93057
EPHX3	4	3609;5632;13956;73730	CATCCCGAGGTGCATCCGAG;GCAAG	2	1.5351133	1.1567204	0.5898707	0.9310963	0.93057
PIWIL2	4	3475;5635;13077;32813	GCATCCGAAGGACAGATGGA;AAGCA	2	1.5346884	1.1634971	0.5904185	0.93057	0.93057
GCDH	4	3050;5647;33497;48720	CCTATGGCAGCGAGGAACAG;GCTGC	2	1.5329917	1.1870999	0.5925495	0.9325369	0.9314612
TJP1	4	1047;5648;7735;42902	GCTAATGCCTCTGAGAGAGA;ACTGA	2	1.5328504	1.4012412	0.592748	0.9314612	0.9314612
CLEC12B	4	3632;5662;53356;68539	GATAACTTATCCCAGCAACT;ATAACCT	2	1.5308762	1.1534333	0.5954133	0.9342592	0.9336828
GNAI2	4	2899;5674;24025;35929	GGCTATCAGGATCATCCACG;GGACC	2	1.5291883	1.1948523	0.5976565	0.9363877	0.9336828
HPS6	4	1806;5676;16253;59619	GTAGGAGAGACCAAGCCGTG;GTGAC	2	1.5289074	1.2876334	0.5980173	0.9355648	0.9336828
SMARCC1	4	2390;5679;60370;72410	CACCAATTCCAGAAAAACCA;TATTCT	2	1.5284862	1.2318238	0.5985713	0.9350463	0.9336828
TTPA	4	1362;5680;28656;63862	CCATGGAGTCCTGAGATCCA;ATAAGA	2	1.5283458	1.3446663	0.5987761	0.9339846	0.9336828
SIAE	4	1459;5685;24869;62097	TTTCAGCTCACTCTGATACG;GCTGGA	2	1.5276445	1.3302389	0.5998281	0.9342455	0.9336828
MOGS	4	1697;5688;33635;49530	TTTGCCACCAACCAGTCCAG;TCAGG	2	1.527	1.2993361	0.6003509	0.9336828	0.9336828
SCAMP4	4	5032;5694;14527;24737	GGCTTCACAGGGATGAACTT;CGGCA	2	1.5263838	1.0933532	0.6014414	0.9340031	0.9340031

C21orf91	4	4694;5699;14714;26175	GCTTAGATCGAGGACAACCC;GTGTG	2	1.5256844	1.1049488	0.6023593	0.9340549	0.9340549
GIPC1	4	2373;5707;37749;44246	GCTCACCATCACGGACAACG;CTGCT	2	1.5245667	1.2312609	0.6037688	0.9348678	0.9348678
TNPO2	4	138;5719;23046;26533	CTGCAGTCCCAAGATCCGGT;GCACA	2	1.5228934	1.8283044	0.6060599	0.9370413	0.9368663
MRAS	4	3243;5724;16176;44949	GGAGCAATACATGCGCACGG;GCGCA	2	1.5221973	1.1701366	0.6069591	0.9370596	0.9368663
UPF3B	4	2059;5730;14573;21264	AAGAAAAGAGAGAAGAAAGG;CATCA	2	1.5213628	1.2576572	0.6079954	0.9372893	0.9368663
PQLC2	4	3584;5736;39472;43552	GGCCCAGCAGCACTCAGCAG;GCATC	2	1.5205294	1.1507088	0.6091378	0.9376816	0.9368663
CRYBB2	4	5136;5737;44815;54607	GTTTCCTTCAGGTTGGGGCA;GCCAC	2	1.5203906	1.0868877	0.6094954	0.9368663	0.9368663
BEND2	4	5027;5748;45358;73560	CATTGCTTCACAGTAGCCCA;GATATG	2	1.5188653	1.0897631	0.6114913	0.938568	0.9378001
GPRASP2	4	3091;5750;11476;11815	GGAGGCTCAAGGAATCACAG;GCAAT	2	1.5185884	1.1773716	0.611879	0.9378001	0.9378001
TMEM178B	4	3585;5769;56213;57953	TATTGTTGTAAATGAAGCCG;GGAGGA	2	1.5159624	1.1483739	0.615325	0.9417147	0.9417147
ALDOB	4	1402;5789;41303;43428	GATGCTGGCGTAGCGAGCCA;GGATT	2	1.5132085	1.3311676	0.6189954	0.9459612	0.9430439
DRD2	4	1135;5792;45470;68042	TTCCCGTCTGACCCGTTGAA;CCTGAT	2	1.5127963	1.3744685	0.619747	0.9457411	0.9430439
CYP4F12	4	3870;5794;62699;73457	CTGTGAAGTCATGCACCAGG;GAAGG	2	1.5125216	1.1326591	0.6201025	0.9449181	0.9430439
NPFFR1	4	5013;5796;33684;50615	ACACATGATGAGCAGCGCCA;GAGCA	2	1.5122471	1.0869286	0.6204819	0.9441339	0.9430439
HADHA	4	1822;5797;11726;31109	GGATCCTGAAGGCAGAAAAG;TATTAA	2	1.5121098	1.2774623	0.6206586	0.9430439	0.9430439
CNIH3	4	2709;5815;18535;34316	ACATAATGCAGAAGAGGCTA;AATGCA	2	1.509644	1.1980779	0.6239799	0.9467281	0.945647
HAGHL	4	63;5816;16892;35069	GCGGGTACCCGAGAACAGGG;CTACA	2	1.5095072	1.9912256	0.6241628	0.945647	0.945647
TYSND1	4	362;5822;19694;70239	GCAGTGGGCCCCAACGGCCG;GGGC	2	1.5086873	1.613747	0.6253385	0.9460708	0.9459756
TMEM91	4	311;5827;47759;55306	GCGCTCACGGAGGGAAGAGG;CATTC	2	1.5080047	1.645933	0.6262626	0.9461135	0.9459756
TMEM189-U	5	423;4578;9324;15148;333	CCAACACCGAGTATGACGAG;ACCAA	2	1.5075522	1.5326768	0.6270672	0.9459756	0.9459756
SPATA25	4	5658;5841;28876;37055	CTGGCTCTACAGGACTACAG;GGACT	2	1.506097	1.0636297	0.6289695	0.9474918	0.9474918
NDUFA5	4	4435;5850;11976;15997	TGCACACAGCCAATCCCACA;ACAAA	2	1.5048732	1.1044618	0.6308104	0.9489114	0.9487344
ZNF17	4	885;5866;33403;62413	TCACCAGAAGTGATGAAGGG;GGCCA	2	1.5027027	1.4212376	0.6339497	0.9522773	0.9487344
GNA14	4	5173;5868;34655;63503	GGAGTGTTACGACAGGAGGA;TCTGA	2	1.5024319	1.0766963	0.634325	0.9514875	0.9487344
TIAF1	4	5511;5873;48263;73684	CATTCTTAAGAACCTGGACC;GGTCCA	2	1.5017552	1.0657893	0.6352813	0.9515703	0.9487344
FAM25A	4	1061;5875;14092;19167	GGAAGAAGTGGTGAAGGAGG;GGAG	2	1.5014847	1.3827979	0.6356649	0.9507962	0.9487344
NDUFB11	4	3310;5876;68787;72057	CCCCAGAACCGACCACACCG;GCAAC	2	1.5013495	1.1558834	0.6358562	0.9497371	0.9487344
TUT1	4	4630;5886;18248;51458	GTTTGTCAGTGGCTTTCCCA;TTTGGA	2	1.4999988	1.0944916	0.6377304	0.9511911	0.9487344
CNTN2	4	4240;5890;14286;18898	ACTGGAATCCTATCTGTGCG;GTTCCC	2	1.4994592	1.109713	0.6385433	0.9510602	0.9487344
TMEM171	4	5832;5893;21581;27858	GACTCCCACACACAACAAGA;GCATG	2	1.4990548	1.0551755	0.6390859	0.9505278	0.9487344
LGR5	4	2420;5894;10872;28056	CTTACATTGTAATAAGAGAA;GCCCAG	2	1.49892	1.2146023	0.6392782	0.9494765	0.9487344
ZC3H6	4	5725;5897;35741;60240	TCTTGCAGTATAATAAACCA;TGACAG	2	1.4985159	1.0579155	0.6400734	0.9493224	0.9487344
UBAC2	4	5455;5908;13359;28858	TGAGAGTGCTTTCAAGTGGA;CTTGG	2	1.4970361	1.0651204	0.642141	0.9510532	0.9487344
PPP1R42	4	2850;5916;7786;10445	CCAGTTTCTTTAATGACCTG;GTTTCT	2	1.4959618	1.1814962	0.6435745	0.9518412	0.9487344
APOA5	4	1906;5917;61003;72260	CTACTTCAGCCAGACCAGCG;GGCCG	2	1.4958276	1.2602854	0.6437533	0.9507741	0.9487344
RAB8B	4	2152;5920;25830;46657	CCTCTGAGAAGCGGAACAGG;CCTCC	2	1.4954252	1.2359301	0.6442938	0.9502434	0.9487344
PPP2R3C	4	1894;5935;62125;71484	CTTACCCTATAATAAAACCG;AATTCTA	2	1.4934167	1.260344	0.6472356	0.9532508	0.9487344
TGFBR3	4	4208;5940;24990;46528	AGGAGAGTCTGGAATGAAGA;ATGAC	2	1.4927484	1.1077068	0.648193	0.9533312	0.9487344
ZNF429	4	2341;5941;43488;71028	TCTGGGTAAGTGTCAAACAT;TCATGT	2	1.4926148	1.217945	0.6483666	0.9522602	0.9487344
NR4A1	4	1947;5943;21318;52257	CATGGACCTGGCCAGCCCCG;TATGG	2	1.4923478	1.254291	0.6487699	0.9515292	0.9487344
GHRH	4	5229;5945;11558;28747	CTTCACCAACAGCTACCGGA;CCATCT	2	1.4920808	1.0697077	0.6491171	0.950718	0.9487344
PAK4	4	2955;5947;22747;36776	GCGCAGGTCCATCTTCTTGA;AGCAC	2	1.4918139	1.1725188	0.6495152	0.9499835	0.9487344
DSG4	4	791;5948;31792;68240	TGGTTGTGAGAGGCTCAGAT;AGTAC	2	1.4916805	1.4392806	0.6498863	0.9492116	0.9487344
TNS4	4	3068;5951;30162;31482	CCACTGCGAAGGGAGCCGAA;GGAG	2	1.4912804	1.1651302	0.6504581	0.9487344	0.9487344
LAS1L	4	2189;5959;27348;27466	GTGACGACCATAAGTTGCAG;GGCCA	2	1.4902146	1.2299554	0.6520485	0.9497424	0.9490906
31-Aug	4	2451;5967;27032;35888	CTTTCTCGTGTACTGCCCGG;GCACC	2	1.4891504	1.2072331	0.6535204	0.9505752	0.9490906
FOXE1	4	974;5970;22811;32109	CTTCCTCAAGATCCCGCGCG;AGTAG	2	1.4887518	1.3942403	0.6540922	0.9500981	0.9490906
LSAMP	4	4080;5984;9837;26558	ATACAGCCTCCGAATCCAGA;AAAGAT	2	1.4868942	1.1103054	0.6567179	0.9526018	0.9490906
ILKAP	4	4171;5985;26971;70539	GGGAGGTCATCAAAGAGCAG;CATCC	2	1.4867617	1.1062893	0.6569196	0.9515872	0.9490906
SENP8	4	3200;5986;14377;58026	GCAGTCATGAAACTGACTGT;GTAGAC	2	1.4866293	1.1548591	0.6571285	0.9505859	0.9490906
MOB3C	4	3201;5992;8992;60353	TGAGCTGTACAAGAAGGCAC;CCAGT	2	1.4858349	1.1544032	0.6582793	0.9509479	0.9490906
STUB1	4	3495;5994;30691;35396	GCCATCGCCAATCTGCAGCG;TCTTCA	2	1.4855704	1.1378748	0.6586608	0.9501991	0.9490906
BCL7A	4	1114;5997;20720;68396	TGATATCAAGAGGGTCATGG;CGGAG	2	1.4851737	1.3645266	0.6592886	0.9498074	0.9490906
VASP	4	2342;5999;43430;44765	ATCATCATAAAGCATCACAG;CCAGAT	2	1.4849093	1.2140085	0.6596899	0.9490906	0.9490906
CCDC175	4	2585;6004;11523;68639	GTTTCGCCTCCAGAATTGCA;GGAGC	2	1.4842489	1.1944333	0.66093	0.9495811	0.9491675
MOAP1	4	448;6013;41367;49885	GGCTCCTTAGACCCAGAGCA;GCAAT	2	1.4830617	1.5554037	0.6626951	0.9508234	0.9491675
RPL14	4	2063;6014;17833;19919	ATTGATGTCTGCCTTCTGCC;TTAGTG	2	1.4829299	1.2380551	0.6628801	0.9497984	0.9491675
FZD1	4	1733;6020;19723;32087	GAGCTGGGAACTTTGTGCCG;AGATG	2	1.4821397	1.2725569	0.6640152	0.9501356	0.9491675
XPO6	4	1076;6026;35056;37765	GCTTCCCCTACAGCAGCAGA;CCTCC	2	1.4813503	1.369815	0.6652252	0.9505789	0.9491675
CA2	4	1955;6034;11703;32492	TGATCATAGGAAACAGACAG;ACCATT	2	1.4802992	1.2474479	0.6667949	0.9515343	0.9491675
PENK	4	1554;6038;13925;40103	GCTTGCGTAATGGAATGTGA;TGTCGC	2	1.4797742	1.2934515	0.6676098	0.9514116	0.9491675
PSG8	4	1630;6040;16517;47177	GCAACCTGTGAGACATAGGG;TCAGG	2	1.4795118	1.2836294	0.6679778	0.9506531	0.9491675
SRPK2	4	2796;6058;10869;11207	GGATGGCGAATACTGCCCAG;TGAGG	2	1.4771549	1.1757571	0.6717585	0.9547469	0.9491675
SEL1L3	4	1060;6062;33320;33423	TCTCTTCTCTAGCTTCCGGG;GCAATG	2	1.4766322	1.3705676	0.6725153	0.9545378	0.9491675
SOX9	4	195;6065;54406;58015	GCTGGTACTTGTAATCCGGG;GCAGC	2	1.4762405	1.7303985	0.6730703	0.9540433	0.9491675
TDRD3	4	3004;6068;9059;37367	TGCCTGGTATCATTTCGAGG;GCTTCA	2	1.4758489	1.1614103	0.6736785	0.9536253	0.9491675
OXT	4	1705;6073;14764;29491	GCAGTTCTGGATGTAGCAGG;GGCCG	2	1.4751968	1.2723726	0.6746785	0.9537623	0.9491675
TMEM86B	4	1882;6083;23727;69631	GCATGGACGCTGGCAAAGCG;ACAGC	2	1.4738943	1.2518556	0.67664	0.9552565	0.9491675
WFDC11	4	2675;6085;16183;32769	TGGGTATCCAGAGCTTCATG;TGTCTG	2	1.4736341	1.1825079	0.6770839	0.9546069	0.9491675
OPN1SW	4	2367;6088;30412;58457	TCAAAAATATCTCTTCAGTG;TGCCCAT	2	1.473244	1.2060953	0.6776462	0.9541259	0.9491675
INSM2	4	2014;6089;39502;66472	GGAGCCTCCTCCAAGAAGGG;CAGCA	2	1.473114	1.2379276	0.6778697	0.9531697	0.9491675
RILPL2	4	1251;6091;18332;51288	ACTGCAGCTGCGTCACCCGG;GGAGC	2	1.472854	1.3343949	0.6782439	0.9524277	0.9491675
TECR	4	1619;6092;13652;39198	GGTCTTCCTAACAGAGTACG;GGGAG	2	1.4727241	1.2816078	0.6784508	0.9514529	0.9491675
FAM181B	4	3239;6097;12322;39602	GGGGAGCCCCCGAAGCCGAA;TTTCC	2	1.4720748	1.1453069	0.6794789	0.9516309	0.9491675
GGACT	4	5819;6103;32312;62885	GGTGCCGTACACGAAGACTA;GTGTA	2	1.4712964	1.0416579	0.6806213	0.9519683	0.9491675
RPL31	4	616;6113;51818;67341	TAGATGGCTCCCGCAAAGAA;AAGGG	2	1.4700011	1.4811979	0.6827772	0.9537206	0.9491675

AUTS2	4	77;6115;33882;50429	CGTCCCGGCCCAGACCCCCG;AATG	2	1.4697423	1.9278902	0.683165	0.9530016	0.9491675
CBX3	4	2109;6117;24372;38496	GGCCTCCAACAAAACTACAT;ACACA	2	1.4694836	1.226956	0.6835839	0.9523279	0.9491675
AIG1	4	350;6118;38300;53580	GGATAGAGCAGTAAGACAGC;CTTTC	2	1.4693542	1.6012956	0.6837897	0.9513596	0.9491675
FEZF2	4	209;6121;11604;52877	GCGCATCATGGCCAAGACGT;AGGG	2	1.4689664	1.7118281	0.6843947	0.9509484	0.9491675
BPIFB2	4	5267;6123;13992;23529	GCACACTGACCATGGAATAG;GCACC	2	1.468708	1.0568055	0.6847803	0.950234	0.9491675
ZNF800	4	5998;6127;29338;34214	CTACAAAAGCGAATGTAAGG;CTGCC	2	1.4681914	1.0352217	0.6856171	0.9501466	0.9491675
ENPP4	4	4847;6128;58248;60595	GGAGTTGTAATTCATAAAAT;TGGAATT	2	1.4680623	1.0705984	0.6858094	0.9491675	0.9491675
PPAPDC3	4	253;6138;25970;30803	GGATGGTGCCTCCGATCCAG;TCGAT	2	1.4667728	1.6696291	0.6878198	0.9507039	0.9501552
KCTD8	4	4059;6146;35905;63805	ACTACTTCAGGGAAGGGCGA;GCAGA	2	1.4657428	1.1006566	0.6894248	0.9516766	0.9501552
C18orf21	4	5353;6155;15419;16351	AACAGAACAGTGAAACATCA;GCCTG	2	1.4645859	1.052035	0.6911806	0.9528547	0.9501552
UPK1B	4	3582;6161;24018;58440	CTACAATGCCTAGAACAGAC;ATAGAT	2	1.4638156	1.1224548	0.692614	0.9535859	0.9501552
SUGCT	4	3670;6171;49414;55505	GTGATGATACACGAACTTGG;CATCA	2	1.4625337	1.1173504	0.6945537	0.9550114	0.9501552
WDR53	4	359;6173;17336;66955	GAACTCCATTAGGACACACG;GCCTG	2	1.4622776	1.5923229	0.6949706	0.9543419	0.9501552
MMAB	4	5218;6176;11247;70895	ACGGGGATACAGGAGCCTGG;GGTG	2	1.4618936	1.0549682	0.695534	0.9538752	0.9501552
FMO3	4	3728;6183;44281;70995	GTAGTACCTGTTCTGCTGTG;ACATTC	2	1.4609985	1.1137101	0.696876	0.9544761	0.9501552
MRPL36	4	893;6184;27708;41695	GCTCTATCTCAGTCGTCACA;TGACG	2	1.4608707	1.3984381	0.6970953	0.9535397	0.9501552
ZNF623	4	3424;6185;16905;25496	ATTCGGACCTAGTTAGGCAT;GCTCTC	2	1.4607429	1.1292685	0.6972783	0.9525561	0.9501552
RFPL4A	4	2949;6198;10296;45527	AGTGGATATGACGTTCGATG;GTGCC	2	1.459084	1.1565408	0.699849	0.9548327	0.9501552
ZNF438	4	2644;6214;22704;29034	AGCAGCTTGGAGTTGATGGA;GTTTG	2	1.4570476	1.1764654	0.7032824	0.958279	0.9501552
TMEM215	4	1328;6230;19831;22696	GGCCTGATGACATTAACCCG;TGAAC	2	1.455017	1.3131894	0.7064903	0.9614095	0.9501552
ADM5	4	5544;6232;17443;61560	AGTATATGATCTCCGCCAGG;CCAGG	2	1.4547636	1.0413079	0.7068999	0.9607288	0.9501552
ADIG	4	541;6233;13997;15236	ACAACAGCAAACCCACAGGG;CTACG	2	1.4546369	1.5010483	0.7071016	0.9597677	0.9501552
XAGE1D	6	3338;4043;19285;19468;2	AGAACCAGCAGCTGAAAGTC;TCTGT	2	1.4536702	1.0622816	0.7087159	0.9558161	0.9501552
XAGE1C	6	3338;4043;19285;19468;2	AGAACCAGCAGCTGAAAGTC;TCTGT	2	1.4536702	1.0622816	0.7087159	0.9558161	0.9501552
XAGE1B	6	3338;4043;19285;19468;2	AGAACCAGCAGCTGAAAGTC;TCTGT	2	1.4536702	1.0622816	0.7087159	0.9558161	0.9501552
XAGE1A	6	3338;4043;19285;19468;2	AGAACCAGCAGCTGAAAGTC;TCTGT	2	1.4536702	1.0622816	0.7087159	0.9558161	0.9501552
XAGE1E	6	3338;4043;19285;19468;2	AGAACCAGCAGCTGAAAGTC;TCTGT	2	1.4536702	1.0622816	0.7087159	0.9558161	0.9501552
PFN2	4	328;6241;44667;63910	AATGCTCTGAAAGACGCCCC;CAAAT	2	1.4536243	1.607335	0.708719	0.9546011	0.9501552
ZNF454	4	5847;6242;24824;45201	ACCAAGACAGAAGCCTCCAG;CTTCA	2	1.4534978	1.0319811	0.7089165	0.9536508	0.9501552
SLC35B1	4	3149;6245;18708;55317	CTTGAAGAAGAAGTACCCGT;GTTGT	2	1.4531185	1.1411278	0.7095163	0.9532433	0.9501552
AP5Z1	4	4776;6252;14290;47860	GATGAGGAAGAGCCTCTGCA;GTTTA	2	1.4522343	1.0652268	0.7109228	0.9539193	0.9501552
SH2D2A	4	861;6253;13246;57661	TCACCTGTAAGTCAGCACGA;GTAGG	2	1.4521081	1.4016985	0.7111099	0.9529594	0.9501552
CBWD3	4	190;6255;14152;63736	GCAACATAGTAAAAGAGTAG;GAGTG	2	1.4518557	1.7238029	0.7115298	0.9511082	0.9501552
CBWD5	4	190;6255;14152;42335	GCAACATAGTAAAAGAGTAG;GAGTG	2	1.4518557	1.7238029	0.7115298	0.9511082	0.9501552
OR13J1	4	5823;6260;28640;41552	GGAGCCGCTCAACAGAACAG;GATGT	2	1.4512251	1.0315108	0.7125143	0.95122	0.9501552
SEC14L4	4	1196;6268;37665;56825	GATCACAGATCCTTACCCTT;ATCCGA	2	1.4502174	1.3323519	0.714271	0.9523614	0.9501552
FAM129B	4	2868;6270;11710;55590	GGTGCTCTACGAAAACAAAG;CTACAT	2	1.4499657	1.1572943	0.7146785	0.951703	0.9501552
POLR3B	4	1409;6280;54221;54431	GCAATGCCTTACCTATCGGC;CGTGG	2	1.4487084	1.2978983	0.7166234	0.9530911	0.9501552
HLTF	4	1138;6281;39611;73289	TCTCAAACTTCCAAATAAAA;TTAGGAT	2	1.4485828	1.3418152	0.7168323	0.9521698	0.9501552
TEKT3	4	2237;6289;16259;52562	CGTACTGCACCAGATCACAG;ATAAG	2	1.4475788	1.2043589	0.7184488	0.953118	0.9501552
BCAT2	4	1015;6293;12309;38911	GTCGGCCAGGAGGGAGACCG;GGTC	2	1.4470773	1.364812	0.7192419	0.952973	0.9501552
CRTC3	4	1897;6294;43117;50101	GAAGGTCTGGGGACAAGCCA;GCAG	2	1.446952	1.2367948	0.7194425	0.9520443	0.9501552
TEX36	4	2762;6298;15774;67495	CCTTCTCCGTGCATGACAAT;TCCTGG	2	1.446451	1.1627539	0.7202222	0.9518831	0.9501552
IL7R	4	6101;6300;27835;72335	CCTGCAGAGAAAGCTCCAAC;ATTCT	2	1.4462006	1.0215066	0.7206057	0.9511996	0.9501552
NPBWR2	4	6030;6302;24167;50197	GGCAGGAGCACATAGAGGAA;CGTCA	2	1.4459503	1.0232493	0.7209904	0.9505191	0.9501552
NGFR	4	141;6305;40871;72781	GGTAGTAGCCGTAGGCGCAG;ACACG	2	1.445575	1.7850015	0.7216141	0.9501552	0.9501552
FBXW11	4	4014;6312;62073;70655	GCTCATCATACTGCAGACAG;TGCCG	2	1.4447002	1.0921411	0.723033	0.9508379	0.9504021
FATE1	4	5506;6315;28082;70887	GAGCTTGGATCTCGGTCCCG;CAACT	2	1.4443255	1.0372244	0.7236016	0.9504021	0.9504021
RNASE12	4	376;6332;24274;67561	GAACGGGAACGTCATTCTGA;GCTTC	2	1.4422064	1.5723897	0.7271806	0.9539164	0.9514458
VWA2	4	2436;6337;40418;48120	GGAAAACAGGAAAACACAGA;TGTGT	2	1.4415844	1.1846478	0.728192	0.954058	0.9514458
TMED7-TIC	4	3809;6338;14944;48460	TGTCGATTAGAAGATCCTGA;GCCGC	2	1.44146	1.1000174	0.7283875	0.9519519	0.9514458
TMED7	4	3809;6338;14944;48460	TGTCGATTAGAAGATCCTGA;GCCGC	2	1.44146	1.1000174	0.7283875	0.9519519	0.9514458
PLEK	4	1422;6341;11128;17456	GATCACTACGACCAAACAGC;TTGCC	2	1.4410871	1.292209	0.728982	0.9515513	0.9514458
ROR1	4	407;6345;12240;33047	GGAGCGGCGGGCGCGTCCCG;GATT	2	1.4405902	1.5546501	0.7298022	0.9514458	0.9514458
MPV17L2	4	3207;6358;18205;19615	TGTTAGTGACGAGCAGCGCG;GCTGC	2	1.4389776	1.1306226	0.7324301	0.9536944	0.9521868
TKTL2	4	990;6360;25850;68964	GTCAACAAACGGCAATCGGG;CCTGT	2	1.4387299	1.3658327	0.7328781	0.9531026	0.9521868
PHLDB3	4	5709;6361;19669;57836	GGAGGCATCCGAAGTCCTGG;TGTTC	2	1.438606	1.0284171	0.7330756	0.9521868	0.9521868
HPCAL4	4	15;6379;7586;31450	GCTGAAACTCCTCCAGGTTG;AGAGC	2	1.4363805	2.265868	0.7370018	0.9561105	0.9561105
SMARCA2	4	6165;6384;14846;27859	GCTCTTGCAGAATTTCCACG;ACTACA	2	1.4357635	1.0146302	0.7380122	0.9562465	0.956214
SIPA1L3	4	2784;6388;50448;73664	TTCTGGGCCCAGAATCCCAA;CTAAG	2	1.4352703	1.1556401	0.7388927	0.956214	0.956214
FUT3	4	6145;6399;20930;59254	TGTCCGTAGCAGGATCAGGA;GGTTG	2	1.4339158	1.0142218	0.741165	0.9579807	0.9579807
MMP24	4	4823;6407;14171;34998	AAGTGGGTGAGCTAGACACG;GGGA	2	1.4329323	1.0538874	0.7428074	0.9589299	0.9589299
PLP1	4	1998;6420;60658;72217	TCCATAGATGACATACTGGA;GCTGAG	2	1.4313371	1.2186278	0.7455871	0.961343	0.9609872
SLC7A7	4	724;6422;21029;48198	GCTGGCCCCAGATTTCTTAA;GGTGA	2	1.431092	1.427615	0.7462211	0.9609872	0.9609872
SFR1	4	4252;6440;16282;52641	ATTTAATTCCTCTTACAATG;AAGTCCG	2	1.4288899	1.0739256	0.7499841	0.9646568	0.9615486
NR2C2	4	4099;6441;12780;18555	TAATAGGCTGAAACAAGCCA;GGAAG	2	1.4287678	1.0804087	0.7501837	0.9637397	0.9615486
ULBP3	4	5585;6442;21921;36950	CTCCTGGTCTACAATGGCAG;TACGG	2	1.4286457	1.0270354	0.7503989	0.9628448	0.9615486
FERMT2	4	841;6448;54003;60073	TTAAGAGAGAAAGTTCTTCG;GATGC	2	1.4279134	1.3945283	0.7516723	0.9633082	0.9615486
CACNA1G	4	1853;6450;40757;42462	CTTGGTCTCTGAGAACTGCG;CTATTA	2	1.4276695	1.2318558	0.7520673	0.9626462	0.9615486
UBQLNL	4	3901;6454;9066;40589	GGACTTGATGACCAAGTAGA;TGGAG	2	1.427182	1.088542	0.7528948	0.9625386	0.9615486
KCNIP1	4	5603;6458;11551;54008	TCTCAATAAAATCGACAGAG;AGATGA	2	1.4266947	1.0255309	0.753716	0.9624233	0.9615486
C6orf120	4	1189;6459;13218;47896	CCTGTCTCCGGGAAGCTGCG;GCAG	2	1.426573	1.3217423	0.7539415	0.9615486	0.9615486
ITGA10	4	1136;6467;24725;59878	GCTGGAATGAAGCATCCACA;TTTCT	2	1.4255997	1.3306878	0.7556432	0.9625564	0.961783
HIC2	4	1985;6473;32251;36258	CCATGATGATGACGTCACAC;CGGCT	2	1.4248706	1.2166955	0.7568719	0.9629599	0.961783
RASD2	4	3074;6489;61276;69830	GGATGTTGTATACCTTACGG;GTGTGT	2	1.42293	1.1305853	0.760454	0.9663531	0.961783
TSPAN16	4	1048;6494;21655;36167	TTTAGTCGCTCCATACCACC;GGATGC	2	1.4223246	1.3457799	0.7615278	0.9665545	0.961783

SCAND1	4	762;6500;10308;10444	GCTCCTTCCAGTTTCTCCGG;GCCTT(	2	1.4215989	1.412095	0.7627908	0.9669953	0.961783
PPP6R2	4	2737;6507;26081;26938	GCTTTGGTACATACTTGAAG;GTTAGG	2	1.4207532	1.151653	0.7643012	0.9677483	0.961783
RNF215	4	5665;6514;17099;25872	TCTCATCCTGAACCACAACG;CAGTCT	2	1.4199085	1.020333	0.7657232	0.9683877	0.961783
DNAJC28	4	5607;6518;53838;72466	CCATCACTTGTTCAGCAGCA;TTATAG.	2	1.4194263	1.0217794	0.7665788	0.96831	0.961783
ZNF80	4	5710;6519;31898;74645	ACATCAGCAGATTCACACTG;GGAGA/	2	1.4193057	1.0187384	0.7667991	0.9674312	0.961783
GATB	4	1501;6521;25371;71344	GCTTTCGCCCGGGTTGACGG;GAACC	2	1.4190648	1.2701601	0.7671973	0.9667784	0.961783
PCDHB14	4	5745;6522;51753;64373	AAGACTCTGGAGACAATGGG;GTGGC	2	1.4189443	1.0175614	0.7673896	0.9658682	0.961783
RNF17	4	570;6524;24231;29934	TAGACAACGCTACTACCCAA;CCTGGT	2	1.4187035	1.4719976	0.7677815	0.965211	0.961783
CAPZA3	4	3165;6530;14824;42625	GTGAATGACCACTATCCAAA;GTTATT/	2	1.4179815	1.1226045	0.7692565	0.9659154	0.961783
ARL17B	4	2395;6532;12738;20384	GTGCCTGCCGTCCCTACAGT;TTACCT	2	1.417741	1.1760424	0.7696661	0.9641369	0.961783
ARL17A	4	2395;6532;12738;20384	GTGCCTGCCGTCCCTACAGT;TTACCT	2	1.417741	1.1760424	0.7696661	0.9641369	0.961783
FAM214A	4	4606;6537;13414;24756	GGTAACAAGACTGTGCTGGA;GTGAA	2	1.4171401	1.0539677	0.7706494	0.9642249	0.961783
ARNT	4	5382;6541;34866;64841	GATGACCCAGAGGCTGGCCA;TTGTC	2	1.4166598	1.0271712	0.7714478	0.9640815	0.961783
SNRPC	4	1715;6542;34071;59031	GCAGGAGGAGGAGCAGAGAA;GTGA(	2	1.4165397	1.2418635	0.7716671	0.9632157	0.961783
ARFGAP1	4	3474;6543;18143;21400	GAAGTACAACAGCAGAGCCG;GCTTC	2	1.4164197	1.1044161	0.7718948	0.9623623	0.961783
CRYGC	4	4537;6549;52280;73517	CCACCCGGATGGAGTTGCAG;GCAG(	2	1.4157001	1.0558842	0.7731141	0.9627459	0.961783
GABARAPL	4	4089;6559;8904;67574	ATATTTCGCTCGAATCTTCG;GGTTCC.	2	1.4145023	1.073714	0.7752212	0.9642327	0.961783
LUM	4	3444;6561;10013;30737	TAGAAAACTCCAAGATAAAA;TCATCC/	2	1.414263	1.1049466	0.7756474	0.9636278	0.961783
CCDC109B	4	4540;6563;28363;47894	CTTACCATCAGGTGGCACCA;GTCAC/	2	1.4140237	1.0549303	0.7760767	0.9630282	0.961783
MDM2	4	2749;6565;11173;17535	TTGCAATGTGATGGAAGGGG;AGACA	2	1.4137846	1.1473274	0.7764987	0.962421	0.961783
CCDC28B	4	862;6567;36055;40367	GCAGCACTCCTTCCTGACCG;TGGCA	2	1.4135455	1.382174	0.7768948	0.961783	0.961783
BYSL	4	3894;6589;63878;69430	CATTGTGGCAGCCTTCTCCA;GCTGA(	2	1.4109211	1.0807371	0.7817836	0.9667019	0.9653571
BGN	4	5757;6592;44529;61871	AGAAGGCCTTCAGCCCACTG;CGACA	2	1.410564	1.0130315	0.7823989	0.9663313	0.9653571
ARID3A	4	5185;6596;18585;70209	TCACAGCTCTACGAACTCGA;CCTCC	2	1.4100881	1.0301337	0.7832378	0.9662373	0.9653571
GHITM	4	2877;6597;18548;65327	TTTCCACCCAGCTTTCACCA;GTACTT	2	1.4099692	1.1366973	0.7834384	0.9653571	0.9653571
ENTPD4	4	6131;6612;10611;34438	ACAGAAAGTCAAAGTGCACG;TCATA/	2	1.4081878	1.0017201	0.7866526	0.9681878	0.9681878
STARD4	4	1893;6638;65816;70202	TATTCTGGAGAACTTTGAAG;TATCCAT	2	1.4051108	1.2162968	0.7923543	0.97407	0.9698697
C2orf72	4	4365;6643;61468;65166	GCACGAAGACCAGCGGCGAG;GCTG(	2	1.4045206	1.0570914	0.793453	0.9742865	0.9698697
SLA2	4	4283;6647;13223;49462	ACGTGGCCAAAGTCTCCCAT;CATGG(	2	1.4040488	1.0602144	0.7943033	0.9741978	0.9698697
PRKACB	4	2210;6651;21489;57900	CATGGCATAATACTGTTCAG;GACCGC	2	1.4035774	1.1847523	0.7951703	0.9741297	0.9698697
OASL	4	3947;6652;21838;22663	GAGAACCGTGCCATTCCCGA;CTGAG	2	1.4034596	1.0745587	0.7954	0.973282	0.9698697
KCNS2	4	6622;6654;37731;68924	GATCTCATCGAAGGAAGACG;GGTCG	2	1.403224	0.9871917	0.795822	0.9726714	0.9698697
MYO5C	4	5422;6656;54508;59518	GGACCTCTCGTTGCCCACCG;GGCAT	2	1.4029885	1.0191038	0.7962597	0.9720812	0.9698697
KRTAP8-1	4	6393;6666;40907;56674	CAGCCAACGCTATATCCCAG;ATCCTG	2	1.4018122	0.9919399	0.7983823	0.973547	0.9698697
EGLN3	4	2815;6667;46430;50207	GGACCTGGAGAAAATTGCCC;ACAGG	2	1.4016946	1.1367286	0.7985933	0.9726812	0.9698697
MT1B	5	3303;5251;31563;32841;4(	AGGAGCAGTTGGGATCCATT;GCACTT	2	1.400987	1.067465	0.7999062	0.9731578	0.9698697
SLC22A1	4	3818;6682;29885;72190	TGGTGGCCAGGCTAGCCAGG;GGGA/	2	1.3999342	1.0788247	0.8018657	0.9744191	0.9698697
MTRNR2L3	4	3577;6685;21506;24589	CACGGATAGATCAATTTCAC;TTGAAT(	2	1.3995826	1.0905959	0.8026848	0.9742933	0.9698697
WFDC2	4	934;6687;19477;44715	GTTCTGGTCAGCCTGGAGCT;GCCCC	2	1.3993483	1.3582912	0.8030902	0.9736662	0.9698697
SERPINB9	4	5088;6689;16650;58838	CTTTGCCCCTAGGAGAACCA;GTTTG/	2	1.3991141	1.0278391	0.803532	0.9730846	0.9698697
TTC39B	4	1236;6692;17193;20027	GCCCGAATAGAGTTGCTGAA;GAGCA.	2	1.398763	1.2998357	0.8041734	0.9727458	0.9698697
LNPEP	4	3597;6699;38792;49312	GCTGAATTAGTCCAATTGAC;GGGTTT	2	1.3979443	1.0887492	0.8056744	0.9734465	0.9698697
TRAF3IP3	4	1008;6701;11748;39147	AGATCCCTGCAGATGCCTGA;TCCTC(	2	1.3977106	1.3415695	0.8060902	0.9728358	0.9698697
NPC1	4	6429;6705;33366;37720	TTATACCTACCTGAAAACAC;TGATAC/	2	1.3972434	0.9887797	0.8069177	0.9727227	0.9698697
MED1	4	4163;6707;31283;74182	ACACAACTCACCACACACCA;GGGTT(	2	1.3970099	1.0617564	0.8073574	0.972143	0.9698697
OR4L1	4	3441;6708;13178;15075	CATGATTGTCCTATAGTGCA;ATGATTG	2	1.3968932	1.0964235	0.8075778	0.9713008	0.9698697
TM9SF4	4	6308;6712;11543;12648	CATCCTTTACTATCATCGGG;GTAGTCT	2	1.3964265	0.9913433	0.8083792	0.9711587	0.9698697
BHMT2	4	1683;6714;26048;52251	AAAAGAATCAGATAGACCCG;GCCTC/	2	1.3961933	1.2354946	0.8088096	0.9705715	0.9698697
TGIF2	4	3204;6716;14415;45748	GGTTCATCAATGCCCGGCGG;GTAACT	2	1.3959601	1.1092897	0.8092326	0.9699769	0.9698697
FAM3A	4	316;6721;12188;33813	AGGGGCCTCACCGTTCACCA;GGAG(	2	1.3953776	1.5861999	0.8103252	0.9701852	0.9698697
MFN1	4	4262;6724;35079;50241	TTATATGGCCAATCCCACTA;CTAGAA/	2	1.3950283	1.0565772	0.8109801	0.9698697	0.9698697
ANKRD31	4	6007;6735;52458;71835	GATGATGCCTGTTCTTAGCG;GATACA	2	1.3937491	0.9977604	0.8133075	0.9715528	0.9707118
ZNF853	4	1297;6736;48335;60475	CTACTATTGCTGCAGCAGCA;AGCAA(	2	1.3936329	1.2873512	0.8135227	0.9707118	0.9707118
PSG4	4	5799;6752;49742;52006	GATCCAGAATGTCACGCAGG;ACAGC	2	1.3917768	1.0024565	0.8171952	0.9739934	0.9710341
AP2A2	4	2716;6759;25065;62601	GGACAGCGTGAAGCAGAGCG;GTAC/	2	1.3909663	1.138242	0.8187212	0.9747121	0.9710341
TMEM56-R\'	5	1677;5323;11350;11650;3\'	ATCCACATGCCATTCTTTGG;ATATTCA	2	1.3905098	1.1898512	0.8195747	0.973533	0.9710341
TMEM56	5	1677;5323;11350;11650;3\'	ATCCACATGCCATTCTTTGG;ATATTCA	2	1.3905098	1.1898512	0.8195747	0.973533	0.9710341
BCL7B	4	6623;6763;48979;61963	CCATTTCCGCACTTTCTCGA;TCCTGT	2	1.3905035	0.9808082	0.8195747	0.9724391	0.9710341
PRPF19	4	4920;6764;19968;70275	AGACCCAGACACTCACCACG;GCTCA	2	1.3903879	1.0291964	0.8197722	0.9715818	0.9710341
NUDT16	4	3246;6766;7553;46598	AGCGGCTGCCGCTTTCCGCG;GCTG/	2	1.3901567	1.1039428	0.8202295	0.9710341	0.9710341
GALT	4	3529;6786;10692;35864	GCTAATGGAGTACAGCCGCC;CCTCA(	2	1.3878488	1.087223	0.8246287	0.9751489	0.9722296
MCPH1	4	644;6788;55718;70020	GTTCATTCATATTAGCTGCA;GCTCGT	2	1.3876184	1.4306003	0.8250383	0.9745419	0.9722296
DTL	4	3788;6789;57362;64493	AGCACAGTAGGAGGTTGCCA;ACAGC	2	1.3875032	1.0740465	0.8252503	0.9737032	0.9722296
TEX13B	4	5180;6792;46011;56117	GGTCTTGGCCTCAAACCTGA;GCTAA(	2	1.3871579	1.0188313	0.8261797	0.9737117	0.9722296
SLC8A2	4	2231;6793;32985;66682	TCTACCGCATCCAGGCCACG;ATAGG(	2	1.3870428	1.1746201	0.8263876	0.972871	0.9722296
ZMYM3	4	4599;6797;12960;13031	GCTGGCCTGGAAAAAGACCC;AAGCT	2	1.3865827	1.0389542	0.8272982	0.9728584	0.9722296
HTR3A	4	4053;6801;25754;51077	GAGCAGTTCTGGACATCGAA;GGAGG	2	1.3861228	1.0611124	0.8281568	0.9727848	0.9722296
NAT1	4	2234;6803;23025;26095	GGTTCTCAAAGGGAACAGCT;TTTGA(	2	1.385893	1.1737804	0.8286048	0.9722296	0.9722296
NAP1L5	4	2524;6814;17597;32816	TGCGGAGCCTAGCCAGGCGG;TGGC(	2	1.3846305	1.1492587	0.8309634	0.973915	0.9723019
SPERT	4	5112;6818;56923;66376	CATGGGGTCTAAAGGCACGG;GATGG	2	1.3841719	1.0195707	0.8318626	0.9738879	0.9723019
ZNF334	4	433;6824;20035;53291	GTACACCAGAAAATTCATGG;CTACAA	2	1.3834847	1.5128786	0.8331911	0.974363	0.9723019
ELK4	4	6593;6825;45670;72399	TCTGTTGGACCTCTAATGAT;CCTCAG.	2	1.3833702	0.9779403	0.8334208	0.9735535	0.9723019
TMEM184B	4	87;6826;49690;69502	CTTCACCACACAGAACTGCA;GGTACT	2	1.3832557	1.85822	0.8336308	0.9727228	0.9723019
ARX	4	2735;6832;35275;59484	GATGCTGTCGATGCAGTAGG;CATGA(	2	1.3825694	1.1327018	0.8349343	0.9731685	0.9723019
TBC1D29	4	1857;6838;50388;65498	CCAGCAAATACATATCCCAC;TTACCC(	2	1.3818837	1.2085305	0.8362919	0.9736762	0.9723019
EXOSC1	4	6810;6840;48380;72964	CGTCTGTGTAACTTGGAGGA;CGAAC(	2	1.3816553	0.9721302	0.8367212	0.9731031	0.9723019

MCMDC2	4	6408;6846;9986;24015	ACTTCTGTCAAGATAGATGA;TCAAGA	2	1.3809705	0.9811533	0.8382472	0.9738054	0.9723019
SORBS3	4	5110;6850;19381;56203	GCACATCTGCCTGATCCGCA;CCTTAC	2	1.3805144	1.0178082	0.839135	0.9737654	0.9723019
NEIL1	4	4571;6852;71923;73535	ACTTTCCCCCAAGGTCCCAG;CCCTC	2	1.3802864	1.0368719	0.8395601	0.9731893	0.9723019
BMP2	4	2362;6856;19430;30732	GCAGCAACGCTAGAAGACAG;GCTCA	2	1.3798308	1.1598028	0.8404905	0.9731995	0.9723019
USP43	4	1873;6866;24685;74371	GCTCAGGGCTTGAAGAACCA;GCTGC	2	1.3786928	1.2052152	0.8426495	0.9746308	0.9723019
LRTOMT	4	5248;6868;16323;68095	TCTGAGAGACTTCAACCAGG;CCAAC	2	1.3784655	1.0122907	0.8430996	0.9740845	0.9723019
RGS7	4	3933;6870;25043;74056	TTATGGGCAGACCAGCAACG;GACTT	2	1.3782382	1.0625907	0.8435996	0.9735969	0.9723019
SLC16A10	4	5023;6874;21993;70518	TCTTTGGTACTGGTAGCAAG;TATGGA	2	1.3777838	1.0193578	0.8445071	0.9735803	0.9723019
PODNL1	4	5437;6880;20414;61565	CTCATCTCCAAGGTGCCCCG;GCCCA	2	1.3771029	1.0057021	0.8457784	0.9739826	0.9723019
BAK1	4	4532;6881;9940;56208	GCAGGTAGCCCAGGACACAG;GGAA	2	1.3769894	1.0367219	0.8459978	0.9731739	0.9723019
P4HB	4	2846;6885;21553;54880	TTCTGCCTTCAGCTTCCCAG;ATACCA	2	1.3765359	1.122052	0.8468803	0.973129	0.9723019
CA10	4	204;6889;22355;37523	GCATACAAGGAGGTGGTCCA;ACTTG	2	1.3760827	1.6706005	0.8477774	0.973101	0.9723019
WDR35	4	1743;6890;9840;53003	AGATGTCTATGATAGCAAGA;TCACAC	2	1.3759694	1.2183117	0.8480019	0.9723019	0.9723019
RNASE2	4	1296;6898;45787;63406	GGTTTCAAACCATTGAGCCC;CATTGA	2	1.375064	1.2782253	0.8499687	0.9734999	0.9731733
GLYATL2	4	3717;6910;11609;41444	CTTGGATGAAGCAATAAGAA;CCTTTC	2	1.373708	1.0706056	0.8525996	0.9754553	0.9731733
GKN2	4	148;6912;18278;60390	GATACAATCTCATGGATACG;TGCATG	2	1.3734823	1.738495	0.8530581	0.9749235	0.9731733
ZNF878	4	6398;6913;14615;74447	GTGTGTGGAGAAATCGGCAT;TTCCC	2	1.3733695	0.9775965	0.8532982	0.9741437	0.9731733
HIST1H4F	4	4762;6919;30881;69881	CGCCGCGTCGGGCCAAGCGA;GGTA	2	1.3726928	1.025963	0.8546028	0.9745794	0.9731733
SLC26A9	4	4586;6925;12281;61987	GCTCTGTCTACCACGTAGCG;GATGA	2	1.3720168	1.0321651	0.8559157	0.9750237	0.9731733
FGF3	4	360;6930;34513;70870	GTTCATGGCCAGGTACCGCC;GTACC	2	1.371454	1.5463159	0.8570622	0.9752777	0.9731733
CBX8	4	268;6934;37327;49072	ATGACAAGCCCAGCTCACCG;ACCAT	2	1.3710041	1.6093689	0.8579832	0.9752748	0.9731733
KIAA1841	4	2849;6935;8466;52461	AATCCCTCAGACTATCAACT;TTTCCC	2	1.3708916	1.1190283	0.858187	0.9744575	0.9731733
SAMHD1	4	1292;6939;7985;13866	GTGCTGCTGAAGAACATCCG;TTACCT	2	1.3704421	1.2765501	0.8590633	0.9744047	0.9731733
OR6C70	4	1132;6948;42722;52669	CATAGGACAGAGCAGCTAGA;CTGGG	2	1.3694316	1.3033343	0.8613242	0.975921	0.9731733
KCNK2	4	713;6954;19398;23164	CCGCTCAGAACTCCAAACCG;TCAGA	2	1.3687588	1.399676	0.8626828	0.9764127	0.9731733
DIDO1	4	6050;6956;19081;57670	GGAGCCCGAGAACGATCAGG;AGCA	2	1.3685347	0.9840123	0.8631381	0.9758821	0.9731733
MEGF6	4	161;6961;40497;70341	CTACAGGCAGGTGTATACCA;CGTGG	2	1.3679747	1.7175727	0.8643013	0.9761521	0.9731733
SYN3	4	2194;6962;12186;12969	GAGTAGAGAGAGTTGACAGC;ATACC	2	1.3678627	1.168329	0.8645175	0.9753531	0.9731733
C5orf42	4	5430;6963;50441;69809	AGAAAAGTCTACTTGCAGCG;TAGGC	2	1.3677508	1.00124	0.8647358	0.9745582	0.9731733
PAX4	4	6048;6964;43820;45940	AGGATCCGTGAGATGTCACA;GGGCA	2	1.3676389	0.9836172	0.8649676	0.9737802	0.9731733
PCDHA9	4	3798;6968;51496;58146	GTGTCGCTGGGAACACTGGA;TCAAG	2	1.3671915	1.06341	0.8658471	0.9737322	0.9731733
DIO3	4	6696;6973;20401;58795	GGAAGCGCGGGAAGAGCACG;CCTC	2	1.3666325	0.9671914	0.8669541	0.97394	0.9731733
R3HDML	4	6096;6974;7476;35608	CAGAGATGTGGCGCTTCCGG;CACCA	2	1.3665208	0.9817973	0.8671932	0.9731733	0.9731733
SCN1A	4	4864;6983;23748;60434	GCAACCTGAGTCAGACCAGT;CTAAG	2	1.3655161	1.0187235	0.8692857	0.9744859	0.9737506
HNF4G	4	811;6989;15822;27051	CACTGTTGAGAGCTCACGCA;AGTAT	2	1.3648471	1.3706181	0.8706475	0.9749774	0.9737506
ING1	4	3062;6991;55041;74356	GCAGGAGCTGGGCGACACAG;GCGC	2	1.3646242	1.1021726	0.8710851	0.9744342	0.9737506
ZNF763	4	1913;6996;10948;20196	ACACAAGAAAGGAATCACAC;TGTTG	2	1.3640673	1.1936719	0.8722046	0.9746541	0.9737506
KLK8	4	595;6999;20227;25060	GACCTCGTGCGGCCAAGACG;ATCCA	2	1.3637335	1.4354113	0.872873	0.9743699	0.9737506
CTAGE1	4	3873;7002;8153;36064	TATGCTTGAAGAAGATGTAA;CTACAG	2	1.3633997	1.0579575	0.8737577	0.9743274	0.9737506
SLC5A3	4	6075;7008;19437;50073	GCTTCTATTAGATTTCCACA;AATATTG	2	1.3627328	0.9804532	0.875136	0.9748351	0.9737506
TMEM57	4	6966;7012;26716;56051	AAAGCGGAACCGGATCACCG;TTTATA	2	1.3622885	0.9590275	0.8759978	0.9747668	0.9737506
TBC1D25	4	6346;7015;16208;16431	ACGTGTTGCTGGCTGAGAAA;TCACA	2	1.3619554	0.9731657	0.8766839	0.9745033	0.9737506
TOPAZ1	4	6839;7019;28559;60338	TCAGACTCGTCAGACCCGCG;TTAAA	2	1.3615116	0.961414	0.8776142	0.9745117	0.9737506
TCP10L	4	937;7025;17737;31520	TGTCATGGAGAAGACAGCTG;GCAGT	2	1.3608465	1.3383704	0.8789625	0.9749836	0.9737506
GRHL2	4	6374;7031;18184;53657	CCACCTGTGCACTATCCCCG;GTAGT	2	1.360182	0.97159	0.8803315	0.9754775	0.9737506
JUNB	4	2908;7032;10073;52213	ACTCCTGAAACCGAGCCTGG;CTGAT	2	1.3600713	1.1097024	0.8805498	0.9746966	0.9737506
OXR1	4	425;7036;13915;45134	TCACTGGGATCACTTAGGTT;GTACCA	2	1.3596287	1.50493	0.8814697	0.9746932	0.9737506
CD8A	4	2804;7037;13561;50041	CCGAGGGGCTGGACACCCAG;ACAA	2	1.3595181	1.1163908	0.8817015	0.9739297	0.9737506
EIF2S2	4	3617;7043;42055;42375	TTTCTGAGCCTGCCCAAGCA;GCCTT	2	1.3588549	1.0681837	0.8830778	0.9744307	0.9737506
GGA2	4	6490;7056;22981;23881	AGGACCTTCAGGCTGCAAAC;TTAAC	2	1.35742	0.9673998	0.886319	0.9769863	0.9737506
ANAPC1	4	1690;7062;38643;74290	GGGACTCTACTAAAAAGCAC;ATGGG	2	1.3567588	1.2149386	0.8877057	0.9774945	0.9737506
SLC12A5	4	4337;7064;14443;19606	CCTGCTGCAGGCCATCTCGA;TCATG	2	1.3565386	1.0342385	0.8881527	0.976968	0.9737506
RNF183	4	6227;7067;24875;48915	GTATGGAACGTGTTGTTGAA;TGAAG	2	1.3562083	0.9732694	0.8887774	0.9766378	0.9737506
TMEM167A	4	1284;7068;19890;50358	CAAGGATCGAATATAAGCAC;TTATTT	2	1.3560982	1.2706559	0.8890228	0.9758919	0.9737506
HERC4	4	4316;7073;40735;73354	ACCCAAACCTAGCTGCCCAA;TTTGG	2	1.3555482	1.0346029	0.890135	0.9760982	0.9737506
PLXNA4	4	4943;7077;9193;50305	TGATGGTGAAGGTGTCCGAA;GTTCT	2	1.3551085	1.0107587	0.8910862	0.9761276	0.9737506
PCGF5	4	4835;7083;57012;67263	TTACCTGCTATATCTGTAAA;ACTTACG	2	1.3544495	1.0142183	0.892424	0.9765801	0.9737506
ODF3B	4	434;7084;9628;57935	TGGCGGCCACACCGGCCCCG;GCAC	2	1.3543398	1.497814	0.8926319	0.9757964	0.9737506
SUV420H1	4	5067;7087;21229;43404	CACACACTACCTACCTCACA;GCAAAA	2	1.3540106	1.0059888	0.8932899	0.9755058	0.9737506
FUBP1	4	6499;7088;7721;62899	TTCTAACTAGTAATTGGCAG;GCTGAG	2	1.3539008	0.9654253	0.8935259	0.9747555	0.9737506
RNF126	4	2032;7090;16256;36503	GCAGTGGCAGAAGTACCGTC;GCAAA	2	1.3536815	1.1764407	0.8940072	0.9742741	0.9737506
PRKCI	4	5927;7092;24469;50334	ACTGAGATGATACTGTACAC;TCAACA	2	1.3534622	0.9797699	0.894449	0.9737506	0.9737506
KLHDC7B	4	3988;7103;12561;13654	ACACACTCTGAGGACAGACA;GGTAG	2	1.3522574	1.0470913	0.8969479	0.9754655	0.9754655
SLC25A30	4	6545;7110;17196;36000	ATGCCTGGCGTAACATCGCG;GTGTG	2	1.3514918	0.9631288	0.8988211	0.976497	0.9755423
AMPD2	4	5397;7113;12847;21635	GGTGGGGCAGAAGCTCTGCA;AGATT	2	1.3511639	0.99396	0.8995633	0.9762989	0.9755423
ZNF311	4	3759;7114;31694;66015	GCACCGGATAATCCACACAG;TCCAG	2	1.3510547	1.0572246	0.8997899	0.9755423	0.9755423
KCNIP3	4	5824;7124;49987;58774	GATGAACAGCAGCACCACCA;GGAGC	2	1.3499631	0.980852	0.9020415	0.9769803	0.9769803
GRHL3	4	6439;7136;25139;31377	AGATATCCTGAAAACCTCCC;GCCACC	2	1.3486556	0.9642437	0.9048055	0.9789699	0.9779568
PCDHB16	4	2507;7137;48955;62776	CTAGATCGAGAGGAGCTATG;GTTGG	2	1.3485467	1.1325309	0.9050384	0.9782196	0.9779568
ANKRD44	4	3449;7140;65770;69055	TGCGGCTCTGAACGGCCACG;TAAAA	2	1.3482203	1.071656	0.9057213	0.9779568	0.9779568
PHKA1	4	1604;7148;18424;67935	GCAGGTTCAAGAATATAAAG;GAGGA	2	1.3473505	1.2208085	0.9075903	0.9789739	0.9788878
FBXO31	4	6768;7152;58076;68003	CGAGCCGGACACAGACCCCG;GCTC	2	1.346916	0.9557009	0.9084375	0.9788878	0.9788878
ATXN7	4	1636;7160;12739;21525	TCACCTGGACAGATTCTGAA;CGAGC	2	1.3460479	1.2161531	0.9104656	0.9800731	0.9800731
PALM2	4	3716;7171;44846;49212	GTACGCCATGGAAATTAATG;CATTCA	2	1.344856	1.0562288	0.9129729	0.9817712	0.9809984
ACSS2	4	1239;7174;31065;46902	CGCTCCGTGGAGGAGCCGCG;CTTC	2	1.3445313	1.27222	0.9136538	0.9815039	0.9809984
FAM120B	4	5872;7176;10046;58220	GCTGTTGACTAAAACCAGAG;AAACC	2	1.3443149	0.9766998	0.9141122	0.9809984	0.9809984

AES	4	4877;7188;24221;59187	CCTGCCCTACCTCTCCCAAG;GATGC	2	1.343018	1.0070162	0.9169178	0.9830103	0.9824921
PLEKHG3	4	6153;7190;13446;65467	GAGGAGGAAGAAGAAATGGG;GCTC	2	1.3428021	0.9684585	0.9173648	0.9824921	0.9824921
PSMF1	4	3540;7199;9682;47100	TTATTGTTGTTCCACCCAGC;CATTGG	2	1.3418313	1.0636392	0.919475	0.9837544	0.9837544
PRSS22	4	1594;7209;19289;29903	CCTTCCTTCCAGGAATACAC;GGGCA	2	1.3407544	1.2187794	0.9218305	0.9852763	0.9844921
TMEFF1	4	1605;7210;22106;63545	GCAGAAGGCGAGCGGAGGCG;CGCC	2	1.3406467	1.2173303	0.922029	0.9844921	0.9844921
OR10K2	4	2297;7222;45996;52925	AGCAAATCTCAGAGCAAGAG;AGTCT	2	1.3393568	1.1450401	0.9249781	0.9866433	0.9866433
STEAP3	4	5137;7227;38875;46204	GGCCCAAGTGACTTTCCAAG;GAAGC	2	1.33882	0.9960696	0.9261652	0.9869127	0.9869127
MAGIX	4	6663;7233;14102;24533	ATCAACGGAGAGTCAACGCA;GCGAA	2	1.3381765	0.9537201	0.9275405	0.9873818	0.9873294
MESP1	4	4477;7243;10864;65931	GCCCCCTCCGTAGGTAGGCG;CACC	2	1.3371052	1.0189206	0.9298118	0.9888029	0.9873294
MAP2K3	4	4769;7244;23498;44909	CCTGGACATCAACATGCGCA;CTACG	2	1.3369982	1.007862	0.9300498	0.988061	0.9873294
ANKFY1	4	2134;7245;8946;65685	GCAGCCAGACTCATCCAGCG;TGACA	2	1.3368911	1.1583253	0.9302962	0.9873294	0.9873294
PDCD7	4	1100;7251;9450;29367	GCAGCCCCGAGCCTCCGCGG;GCCT	2	1.3362494	1.2926867	0.9316787	0.9878039	0.9876743
TP53INP2	4	674;7256;24884;24964	CCAGCTCTCGTCCATCAAGG;CCTCC	2	1.3357151	1.3950256	0.9328845	0.9880904	0.9876743
NR0B1	4	121;7262;56029;63980	GCAGCGGTACAGGAGTGCCA;GCAG	2	1.3350744	1.7627933	0.9342785	0.9885753	0.9876743
IGDCC3	4	2385;7263;28618;65337	CCAGCACTATAGGCTGCCCG;CAGTA	2	1.3349677	1.1354742	0.9347369	0.9880703	0.9876743
ING5	4	6415;7268;29626;74648	GGCTGCAGAGTACATCTCCA;GATCT	2	1.3344344	0.9577149	0.9359199	0.9883314	0.9876743
MMP9	4	3723;7271;12615;15952	GTACCGCTATGGTTACACTC;ATCCTA	2	1.3341146	1.0505137	0.9366392	0.9881029	0.9876743
HGH1	4	726;7278;26905;45504	ACTGAGGTTGGCTAGCGCGG;CCTG	2	1.3333689	1.378172	0.9382089	0.988771	0.9876743
FBXL14	4	3235;7282;39213;55429	GGCTCAGGATCTGCACCCGG;GTAG	2	1.3329432	1.0759729	0.9391382	0.9887636	0.9876743
OR5M9	4	3380;7285;48796;68034	TTTCATTGCCGTTGTCCACG;ATAGAC	2	1.3326241	1.0676155	0.9398045	0.9884796	0.9876743
ETHE1	4	6163;7297;41922;46248	TTGATCAGCTGGGCATCCCG;GCTGA	2	1.3313492	0.9624745	0.9426922	0.9905304	0.9876743
BMP7	4	4342;7302;35326;53777	TACAGGTCCAGCATGAACAT;GGACCT	2	1.3308187	1.0211746	0.9438658	0.9907777	0.9876743
UGT2B11	4	6192;7305;43652;46227	ATATGTCATATAATTCCCAC;TTGTGTA	2	1.3305006	0.9613065	0.9445821	0.9905448	0.9876743
CAPN7	4	4755;7307;8405;24941	TTTACCATATACTTCCCACA;TTATTAA	2	1.3302886	1.0050151	0.9450498	0.9900522	0.9876743
KPTN	4	3827;7309;10324;39381	TCAACAAGTCACCCCCCAAG;TCTGT	2	1.3300767	1.0434674	0.9455187	0.9895616	0.9876743
PTPRT	4	3845;7321;15574;22543	GCGACTTCAGAAGTCACGGA;CATCA	2	1.3288065	1.0419789	0.9485675	0.9917696	0.9876743
C6orf211	4	5249;7324;12523;18155	TGTGATCTGTCTCTCTCAGG;AACCTC	2	1.3284893	0.9872706	0.9492681	0.9915204	0.9876743
EEF2K	4	6189;7328;37030;44408	TGTGGTGTCTTTCAGCCCTT;AGAAG	2	1.3280666	0.9601663	0.9502214	0.9915353	0.9876743
CCL21	4	2606;7329;16480;50765	TGTCCACTCACAGGATAGCT;GTAGCT	2	1.327961	1.1147221	0.9504365	0.9907808	0.9876743
ZDHHC18	4	5634;7330;60411;71298	GTACCAGCAGATCAGCCCCG;GCCG	2	1.3278554	0.9752055	0.950686	0.9900635	0.9876743
ZNF107	4	5779;7331;38482;48077	AGGAGAAAATTCATACTGGA;GGTTAG	2	1.3277498	0.9710041	0.9509168	0.9893282	0.9876743
NOL3	4	1418;7334;16734;42740	GCTTGGGACTGGCAGCACGT;ACGCT	2	1.3274331	1.2359581	0.9516413	0.9891075	0.9876743
C19orf80	4	673;7335;18660;56475	GGTCCTGTACACACCGTTGA;GCCCC	2	1.3273276	1.3911455	0.9519002	0.9884037	0.9876743
LILRB1	4	5807;7336;61703;64365	ATTTGTTCTGTATAAGGACG;TCCCTC	2	1.3272221	0.9699554	0.952133	0.9876743	0.9876743
MEX3B	4	6625;7348;14862;71974	TGTTCTTATTCCGGGAGGCG;CCACC	2	1.3259572	0.9484886	0.9549646	0.9896395	0.9888879
CSPG5	4	6728;7350;45006;67825	TCTCTCCCTCAGTATCAGGG;GGACA	2	1.3257466	0.9460199	0.9554282	0.9891492	0.9888879
MID1IP1	4	1255;7353;7541;25034	CGTGAACAACATGGACCAGA;GATGT	2	1.3254308	1.2600256	0.9561122	0.9888879	0.9888879
POLR1E	4	3451;7366;42041;48017	CTACCTGGTGAACTGACCTG;GTAAC	2	1.3240643	1.0594704	0.9592193	0.9911307	0.9906323
WWC2	4	6628;7368;13671;19324	TCTTGGATCTTCTATCTGCG;AACCCT	2	1.3238543	0.9473676	0.9597151	0.9906737	0.9906323
KDELC2	4	3011;7374;42352;65979	CTAAAGATCTCCGGTAAACA;GGTCC	2	1.3232247	1.0846563	0.9613939	0.9914375	0.9906323
BPIFB3	4	7266;7378;9648;17612	CCAGCACACTGTCCACCACG;TGTGG	2	1.3228052	0.9330222	0.9623575	0.991463	0.9906323
SPANXB1	4	5546;7382;17001;21376	TTGGCCTCGTTGGATTCACA;GGACG	2	1.3223861	0.9750597	0.9632702	0.991436	0.9906323
THOC7	4	279;7384;26728;51019	GAGATGGTGCTGGAGATGAT;ACTCT	2	1.3221766	1.5763168	0.963764	0.9909784	0.9906323
RNMTL1	4	3924;7389;42212;62320	GTAACGCCACAAGGAATAAT;TGAGCC	2	1.3216532	1.0347128	0.964948	0.9912306	0.9906323
PGLYRP1	4	7098;7393;16663;29013	GCTGCAGCCCCATAGTGCCC;GATAC	2	1.3212347	0.9356954	0.9659781	0.9913245	0.9906323
AQP2	4	5124;7397;20804;56821	TCTCCGAGCCGCCTTCTACG;CGTGA	2	1.3208165	0.9874963	0.9669501	0.9913585	0.9906323
RABL3	4	808;7398;24687;61858	GTCATTGGAAGCTCTCAACA;ATGGA	2	1.320712	1.3493288	0.9671798	0.9906323	0.9906323
C7orf55	4	3910;7411;20742;36547	GCTCCCGTACCCGATGTGCA;CACAA	2	1.3193549	1.0342109	0.9702993	0.9928644	0.9915643
CRAMP1L	4	2531;7425;12454;43584	GCCCAGGCGTGGTTGTTCCG;AAGA	2	1.3178963	1.1153535	0.9739397	0.9956247	0.9915643
PRSS46	4	3508;7426;35333;65270	GTAAGGCTTTGAAGATCCCC;GAAGC	2	1.3177923	1.0532986	0.9741819	0.9949092	0.9915643
ATP5E	4	5867;7428;53006;59632	TTGCACAGATCTGGGAGTAT;CCGCTT	2	1.3175842	0.9634721	0.9746393	0.9944146	0.9915643
ABTB1	4	3448;7429;17162;36907	CCAACATGCTGGACACCAAA;GCTCT	2	1.3174802	1.0563398	0.9748846	0.9937048	0.9915643
CXorf65	4	5465;7430;12942;24140	TTCATCTTCATCAAACATGG;CGTTTG	2	1.3173762	0.9749868	0.9751434	0.9930101	0.9915643
SFXN5	4	5151;7432;8360;42204	CAGTGACAAAGAGTGTGCGA;GGATA	2	1.3171683	0.9847838	0.9756122	0.9925304	0.9915643
GRIPAP1	4	5298;7443;9315;40388	GTTGACCCAGGAATTACAGG;GGTGT	2	1.3160257	0.979481	0.9782515	0.9942576	0.9915643
SAPCD1	4	7105;7445;31131;56049	TTGGGCTGATACTCACCCAA;GTTTG	2	1.3158182	0.9328405	0.97871	0.993767	0.9915643
AKTIP	4	3619;7452;40142;41451	TGAAGAGAAGACATTAACAG;GATGG	2	1.3150923	1.0462008	0.9803877	0.9945143	0.9915643
IGFBP4	4	6381;7464;51399;57841	CCAGCAGCAGGGCGGCCACG;GCCA	2	1.3138498	0.9482523	0.9832474	0.996458	0.9915643
ZSCAN23	4	4210;7466;68880;73927	CTGCATCTCTGCAGTCTGAA;ACTTG	2	1.3136429	1.0180693	0.9837204	0.9959815	0.9915643
KRTAP10-4	5	4692;5904;18902;37319;4	CGCAGGTCACTGGGCAGCAG;TGGT	2	1.311489	0.963559	0.984473	0.9948358	0.9915643
KRTAP10-1	5	3255;5904;15714;72014;7	TCACAGGTCACTCGGCAGCA;TGGTG	2	1.311489	1.0253109	0.984473	0.9948358	0.9915643
GSTT2	5	468;6811;18011;24724;28	GTGATTTCATCTTGACCGAA;CCCACC	2	1.2046546	1.3598017	0.9863867	0.9958168	0.9915643
WFDC10B	5	2444;7134;7892;25020;29	TCTGCATCCTCATCTTGTCA;AGGACA	2	1.1706249	1.0072979	0.9871829	0.9956688	0.9915643
TBC1D3B	6	4834;6109;14857;24126;3	GGAATGCCCATGAACATCCG;CCTCC	2	1.1466993	0.8513931	0.987738	0.995278	0.9915643
CBWD1	6	190;6255;40229;49052;65	GCAACATAGTAAAAGAGTAG;GAGTG	2	1.1298873	1.4758793	0.988079	0.9937252	0.9915643
CBWD6	6	190;6255;9526;10491;141	GCAACATAGTAAAAGAGTAG;GAGTG	2	1.1298873	1.4758793	0.988079	0.9937252	0.9915643
TMSB15B	6	5937;6501;8693;35638;61	ACATGACTTACTTTCCTTTG;CGGAAG	2	1.1026376	0.802013	0.9886424	0.9933457	0.9915643
OBP2A	6	2843;6540;15353;33495;5	GACCTGGTACGTGAAGGCCA;GTGAA	2	1.0984364	0.9120129	0.9887474	0.9915643	0.9915643
OBP2B	6	2843;6540;15353;33495;5	GACCTGGTACGTGAAGGCCA;GTGAA	2	1.0984364	0.9120129	0.9887474	0.9915643	0.9915643
MRPL2	8	4968;6246;15501;28909;3	GTGAAGATGAGGAAGTCTGG;CCACA	2	0.9357001	0.7094653	0.9932942	0.995179	0.995179
DESI1	8	1860;6938;27819;29368;5	GGAATATTTGAAATTTCCCC;AGGGGA	2	0.8709162	0.8241132	0.9953129	0.9962563	0.9962563
SCAF1	8	3263;7446;8523;20574;22	GCAGCAAGAAGAAGAAGAAG;TCGAT	2	0.829151	0.7107475	0.9969407	0.9969407	0.9969407
