# Supplementary material for: CRISPR Screen Reveals that EHEC’s T3SS and Shiga Toxin Rely on Shared Host Factors for Infection
Source: mBio. 2018 Jun 19;9(3):e01003-18. doi: 10.1128/mBio.01003-18 (PMC6016243; doi:10.1128/mBio.01003-18)
Supplement: TABLE S3 [file mbo003183919st3.pdf]

Table S3. Sequence of sgRNAs and plasmids used to construct HT-29 Cas9 and HeLa Cas9 CRISPR mutants.

Gene ID	Construct ID	sgRNA sequence
**A4GALT**	**9873**	**ACTGGTACGCGGCCGTGCAG**
**UGCG**	**3574**	**CCTTACGTAGCAGACAGACA**
**SPTLC2**	**1793**	**ACAACTATCTTGGATTTGCA**
**B4GALT5**	**7856**	**GAGAGAAAAGAAGAAGAGCG**
**ARF1**	**5085**	**GCTGATGTTCTTGTACTCCA**
**LAPTM4A**	**1045**	**GCACCGGGACGATCATCCTG**
**TM9SF2**	**7382**	**TGGATAATATGCCTGTAACG**
**DUSP6**	**7645**	**CATCGAGTCGGCCATCAACG**
**ZNF217**	**8929**	**GCGCAGACAGACTCTCCACA**
**MLLT3**	**7327**	**GGAATAAGTCATAATCAAAG**

	Source
pLenti-A4GALT	this work
pLenti-UGCG	this work
pLenti-BGALT5	this work
pLenti-ARF1	this work
pLenti-LAPTM4A	this work
pLenti-TM9SF2	this work
pLenti-DUSP6	this work
pLenti-ZNF217	this work
pLenti-MLLT3	this work
