# Supplementary material for: CRISPR Screen Reveals that EHEC’s T3SS and Shiga Toxin Rely on Shared Host Factors for Infection
Source: mBio. 2018 Jun 19;9(3):e01003-18. doi: 10.1128/mBio.01003-18 (PMC6016243; doi:10.1128/mBio.01003-18)
Supplement: TABLE S4 [file mbo003183919st4.pdf]

Table S4. Antibodies used for western blot and immunofluorescence.

Antibodies	Source	Identifier	Dilution	Western Blot	Immunofluorescence
TM9SF2	LS Bio	LS-B12359	1:500	X	
LAPTM4A	Sigma	AV47057	1:1000	X	
SPTLC2	Invitrogen	PA5-21142	1:250	X	
UGCG	Novus Biologicals	H00007357-M03	1:500	X	
ARF1	Santa Cruz Biotech.	sc-53168	1:1000	X	
B4GALT5	Abcam	ab56558	1:500	X	
A4GALT	Abcam	ab98998	1:500	X	X
B-ACTIN	Santa Cruz Biotech.	sc69879	1:1000	X	
TGN46	Abcam	ab50595			X
GM130	Abcam	ab52649	1:1000		X
58K	Abcam	ab27043			X
TM9SF2	Dr. Yusuke Maeda		1:500		X
